# Genome-wide landscape establishes novel association signals for metabolic traits in the Arab population

**DOI:** 10.1007/s00439-020-02222-7

**Published:** 2020-09-09

**Authors:** Prashantha Hebbar, Jehad Ahmed Abubaker, Mohamed Abu-Farha, Osama Alsmadi, Naser Elkum, Fadi Alkayal, Sumi Elsa John, Arshad Channanath, Rasheeba Iqbal, Janne Pitkaniemi, Jaakko Tuomilehto, Robert Sladek, Fahd Al-Mulla, Thangavel Alphonse Thanaraj

**Affiliations:** 1 Dasman Diabetes Institute, P.O. Box 1180, 15462 Dasman, Kuwait; 2 Faculty of Medicine, University of Helsinki, Helsinki, Finland; 3 King Hussein Cancer Center, Amman, Jordan; 4 Sidra Medical and Research Center, Doha, Qatar; 5 Department of Public Health, University of Helsinki, Helsinki, Finland; 6 Department of Public Health Solutions, National Institute for Health and Welfare, Helsinki, Finland; 7 McGill University and Genome Quebec Innovation Centre, Montreal, Canada

## Abstract

While the Arabian population has a high prevalence of metabolic disorders, it has not been included in global studies that identify genetic risk loci for metabolic traits. Determining the transferability of such largely Euro-centric established risk loci is essential to transfer the research tools/resources, and drug targets generated by global studies to a broad range of ethnic populations. Further, consideration of populations such as Arabs, that are characterized by consanguinity and a high level of inbreeding, can lead to identification of novel risk loci. We imputed published GWAS data from two Kuwaiti Arab cohorts (*n* = 1434 and 1298) to the 1000 Genomes Project haplotypes and performed meta-analysis for associations with 13 metabolic traits. We compared the observed association signals with those established for metabolic traits. Our study highlighted 70 variants from 9 different genes, some of which have established links to metabolic disorders. By relaxing the genome-wide significance threshold, we identified ‘novel’ risk variants from 11 genes for metabolic traits. Many novel risk variant association signals were observed at or borderline to genome-wide significance. Furthermore, 349 previously established variants from 187 genes were validated in our study. Pleiotropic effect of risk variants on multiple metabolic traits were observed. Fine-mapping illuminated rs7838666/*CSMD1* rs1864163/*CETP* and rs112861901/[*INTS10,LPL*] as candidate causal variants influencing fasting plasma glucose and high-density lipoprotein levels. Computational functional analysis identified a variety of gene regulatory signals around several variants. This study enlarges the population ancestry diversity of available GWAS and elucidates new variants in an ethnic group burdened with metabolic disorders.

**Electronic supplementary material:**

The online version of this article (10.1007/s00439-020-02222-7) contains supplementary material, which is available to authorized users.

## Introduction

The post-oil era of the Arabian Peninsula has witnessed a substantial increase in the prevalence of metabolic trait-related disorders, such as obesity, dyslipidemia, hypertension, and type 2 diabetes mellitus (T2D), in its population. Despite the high prevalence of metabolic-related disorders in the Arabian Peninsula (Abuyassin and Laher 2015; Al Rasadi et al. 2016; Al Sifri et al. 2014; Channanath et al. 2013; Klautzer et al. 2014; Ng et al. 2014; Tailakh et al. 2014), there is a lack of convincingly identified genetic determinants for metabolic traits in people from this region. The global genome-wide association studies (GWAS) performed for metabolic diseases and traits overrepresent people of European ancestry (Mills and Rahal 2019). Only a few GWAS from the Arabian Peninsula are reported in the literature, including those on unrelated individuals from Saudi Arabia (Ram et al. 2017; Wakil et al. 2016) and Lebanon (Ghassibe-Sabbagh et al. 2014), on an extended family from the United Arab Emirates (Al Safar et al. 2013), and our own studies from Kuwait (Hebbar et al. 2017a, b, 2018, 2020). Although these studies identified few novel risk variants, they achieved virtually no success in replicating risk variants that had been discovered in non-Arab populations. While the inability to reproduce established genetic risk variants in these studies may possibly be attributed to different genetic architectures in terms of gene–environment interactions (Fahed et al. 2012) and a different pattern of metabolic disorders (such as dyslipidemia (Al Rasadi et al. 2016)) in Arab populations, it is more likely to be caused by technical factors, such as insufficient genome coverage provided by single nucleotide polymorphism (SNP) arrays that are used to genotype the discovery cohorts (Hebbar et al. 2019).

Imputation and meta-analysis have become standard practices in GWAS delineating genetic risk loci associated with complex disorders (Marchini et al. 2007; Pei et al. 2010). The imputation of genotypes for genetic variants that are untyped in the array increases the information provided by each microarray by accurately evaluating the evidence for association at genetic markers that are not directly genotyped (Li et al. 2009). Current publicly available imputation reference panels, many of which are based on haplotypes identified by the 1000 Genomes (1000G) Project (1000 Genomes Project Consortium et al. 2015), accurately predict genotypes both for common variants (minor allele frequency [MAF] ≥ 5%) and low-frequency variants (0.5% ≤ MAF < 5%) across diverse populations (Mitt et al. 2017; Vergara et al. 2018). For example, Ghassibe-Sabbagh et al. (Ghassibe-Sabbagh et al. 2014) derived a marker set of > 5 million SNPs (either directly genotyped or imputed using the 1000G Project reference panels), showing an association with two established T2D loci (rs7766070/*CDKAL1* and rs34872471/*TCF7L2*) at genome-wide significance in a Lebanese population sample. Meta-analysis improves the capability to detect associations (Zeggini and Ioannidis 2009) by combining summary results from independent GWAS (usually with imputed genotypes) and has helped to markedly enlarge the catalog of GWA-identified risk loci from single studies for disorders such as T2D (Mahajan et al. 2018; Saxena et al. 2012).

Since the advent of GWAS, many risk loci for metabolic traits have been globally identified, concentrating mainly on the European population (Bustamante et al. 2011; Need and Goldstein 2009). Although there has been an increase in the number of GWAS from Asia (mostly East Asia) and Africa, the Eurocentricity remains prominent (Popejoy and Fullerton 2016). Studies have shown that (i) while a large number of Eurocentric risk loci associations replicate in one or the other non-European population, novel risk loci are often discovered in ethnic populations; and (ii) Eurocentric associations can have different effect sizes in ethnic populations (Carlson et al. 2013). There is a lack of studies that evaluate the transferability of established risk loci in Arab populations. Furthermore, Arab populations are characterized by a high level of inbreeding due to the practice of consanguineous marriages, often between first cousins. The consideration of such ethnic populations can lead to the identification of novel risk loci.

In the present study, we imputed two genome-wide genotype datasets from two independent cohorts of Arab ethnicity (from Kuwait) using the 1000G Phase 3 haplotype reference panel. We also performed statistical tests for associations with well imputed common variants (≥ 5% frequency) of 13 quantitative metabolic traits. We combined summary statistics from the imputed datasets using meta-analysis and compared the resulting associations with those reported in global GWAS (as listed in the GWAS Catalog, which compiles the results of published GWAS as a curated resource of SNP-trait associations (Welter et al. 2014)) or in other genetic studies published in the literature. Our study identified 70 risk variants from nine genes associated with metabolic traits at genome-wide significant p values. Many other risk variants were observed at p values borderline to genome-wide significance or with suggestive evidence of association. Of the identified variants, 349 were established variants reported in global GWAS or in the literature. The established markers seen replicating in our study cohort were characterized in comparison with those that are not seen in our study cohort for effect size and projection of the required sample size. Identified variants were characterized by computational functional analysis. We performed fine-mapping analysis to identify candidate causal variants.

## Results

### Characteristics of the study cohorts

Table 1 presents the demographic and clinical characteristics of the study participants. The quality control (QC) procedures resulted in retaining 1434 samples and 118,793 SNPs using the MetaboChip and 1298 samples and 674,131 SNPs using the OmniExpress BeadChip. The two cohorts were genotyped in different times but at the same site. A total of 34,800 SNPs was genotyped in both the BeadChips. Only in the case of 15 SNPs, the proportional test p values for differences in allele frequencies were significant; however, the significance vanished when Bonferroni correction was applied. We performed principal component analysis by merging the SNPs with data from 1000G Project populations and performing linkage disequilibrium (LD) pruning to estimate the heterogeneity of each study cohort. The LD-pruned dataset included 43,537 SNPs for the cohort genotyped using the MetaboChip and 66,645 SNPs for the cohort genotyped using the OmniExpress BeadChip. Scatterplots presenting the first three principal components derived from a merged dataset of the samples genotyped on each of the two BeadChips and representative populations from the 1000G Project are presented in Supplementary Figure S1 (A–F). These scatterplots depict similar positioning for the individuals from both of the study cohorts.

**Table 1:** Demographic and clinical characteristics of the participants in the two GWAS cohorts from Kuwait

Trait	Participants genotyped using OmniExpress (*N* = 1298) (mean ± SD)	Participants genotyped using MetaboChip (*N* = 1434) (mean ± SD)	All participants (*N* = 2732) (mean ± SD)	*p* value^$^
Sex, male:female	658:640 (50.69%)	808:626	1466:1266	0.825
Age, years ± SD	47.15 ± 13.58	45.73 ± 11.20	46.41 ± 12.40	0.003
Weight, Kg ± SD	88.68 ± 21.09	87.48 ± 18.25	88.05 ± 19.65	0.112
Height, cm ± SD	164.94 ± 13.25	166.02 ± 11.76	165.92 ± 9.27	0.009
BMI, Kg/m^2^ ± SD	32.54 ± 8.52	31.54 ± 6.30	32.00 ± 6.75	0.001
WC, cm ± SD	102.29 ± 16.29	101.56 ± 13.28	101.91 ± 14.78	0.201
LDL, mmol/dl ± SD	3.08 ± 0.98	3.34 ± 1.01	3.22 ± 1.00	3.78E−15
HDL, mmol/dl ± SD	1.13 ± 0.37	1.13 ± 0.33	1.13 ± 0.35	0.459
TC, mmol /dl ± SD	4.95 ± 1.10	5.22 ± 1.06	5.09 ± 1.09	6.72E−11
TG, mmol /dl ± SD	1.73 ± 1.21	1.58 ± 1.04	1.65 ± 1.12	0.0028
HbA1c, mmol/L ± SD	7.11 ± 2.08	6.23 ± 1.67	6.55 ± 1.87	< 2.2E−16
FPG, mmol/L ± SD	7.34 ± 3.55	6.20 ± 2.63	6.74 ± 3.14	< 2.2E−16
SBP, mmHg ± SD	127.72 ± 17.69	129.65 ± 17.38	128.75 ± 17.55	0.004
DBP, mmHg ± SD	77.94 ± 10.65	78.59 ± 11.17	78.29 ± 10.93	0.825
Obesity (BMI ≥ 30 kg/m^2^) (yes:no)	778:520 (59.9%)	808:626 (56.35%)	1586:1146 (58.05%)	0.057
Diabetic (yes:no)	601:697 (46.3%)	471:963 (32.8%)	1072:1660 (39.23%)	6.31E−13
Hypertensive (yes:no)	424:874 (32.66%)	375:1059 (26.15%)	799:1933 (29.20%)	1.85E−04
Lipid lowering medication (yes:no)	151:1147 (11.63%)	103:1331	254:2478	6.32E−05
Glucose lowering medication (yes:no)	303:995 (23.34%)	117:1317	320:2412	4.34E−28
Anti-hypertensive medication (yes:no)	155:1143 (11.94%)	138:1296	293:2439	0.051

^$^Significance of difference between the two study cohorts. Student’s *T* test was used for quantitative traits and Chi-square test was used for binary traits

### Flow of data through different analyses tools of the study pipeline

**Fig. 1:**
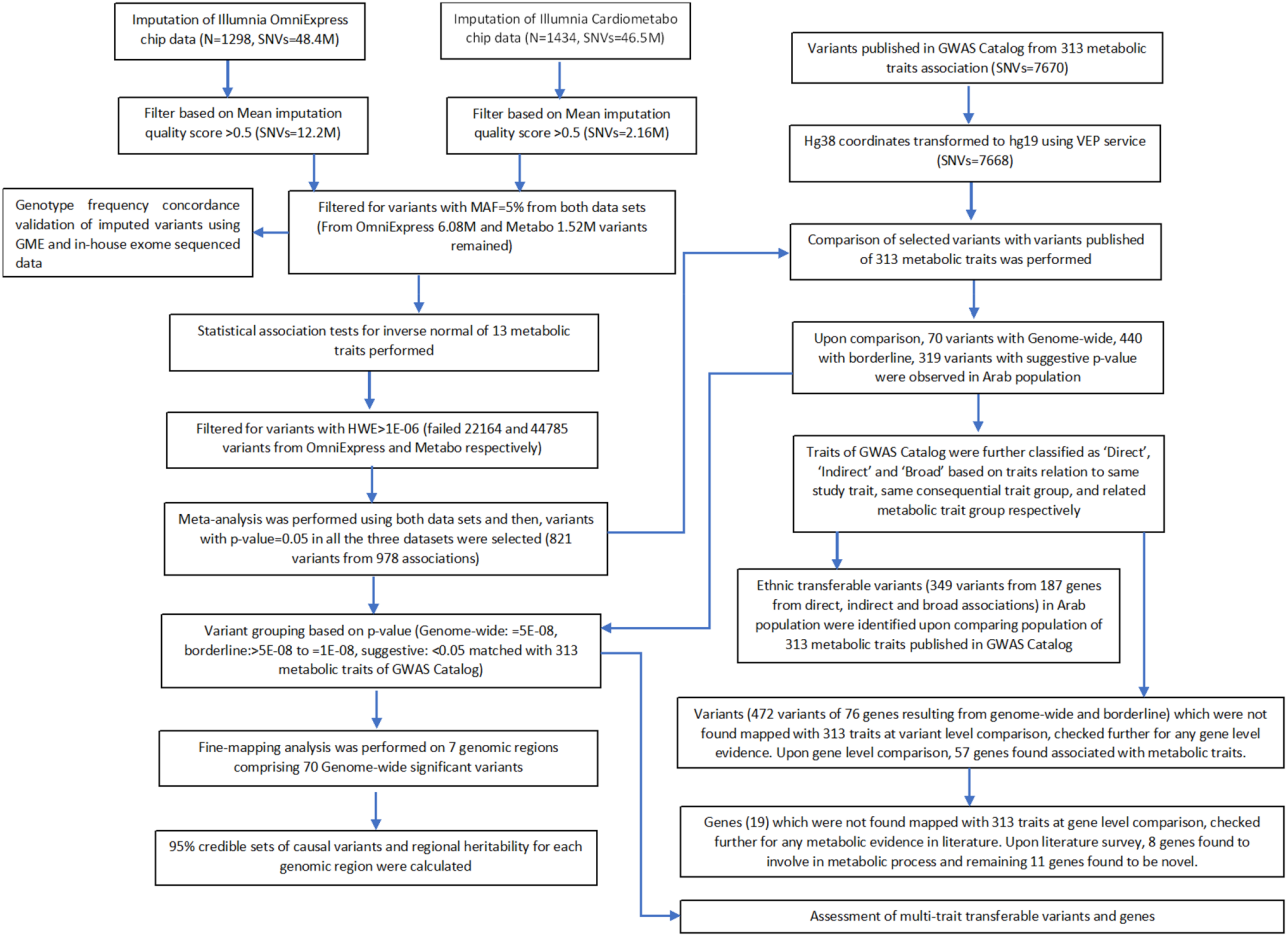
Flow of data from the two genotyping platforms to the steps of imputation, meta-analysis and to the characterization of the resultant association signals. Characterization of the signals was carried out by way of comparing to association signals published in GWAS Catalog for the 313 search terms relating to metabolic traits

Figure 1 presents the flow of data from the two genotyping platforms through the imputation, meta-analysis, and QC stages to the final choice and characterization of association signals.

### Imputation and quality score

Our imputation strategy used 1000G Project Phase 3 data (1000 Genomes Project Consortium et al. 2015). Utilizing the Michigan Imputation Server (MIS), we imputed 48,424,667 markers for the 1298 samples genotyped with the OmniExpress BeadChip and 46,597,973 markers for the 1434 samples genotyped with the MetaboChip (Supplementary Table S1). Supplementary Figure S2A presents a plot of the imputation mean quality score (Rsq) for the imputed markers at various MAF values. As expected, the Rsq increased as the MAF increased, and at every MAF value, the Rsq was higher with the OmniExpress BeadChip platform, which has a higher array SNP content than the MetaboChip platform.

### Final dataset of imputed markers

Supplementary Figure S2B shows the proportion of imputed variants falling into different MAF ranges for each of the two platforms before and after filtering the variants at Rsq > 0.50. We observed that a large proportion of variants with an MAF < 5% had poor Rsq values, whereas those with an MAF ≥ 5%, constituting a smaller proportion of the total variants, were confidently imputed with an average Rsq > 0.75 on both platforms. Using a threshold of ≥ 0.5 for the imputation mean quality score, 12.2 million and 2.16 million SNPs were imputed for the OmniExpress BeadChip and MetaboChip, respectively. To create our final dataset, we filtered these for common markers (MAF ≥ 5%), resulting in an imputed OmniExpress dataset of 6.08 million markers and an imputed MetaboChip dataset of 1.52 million markers (mean Rsq = 0.822 and 0.759, respectively) (Supplementary Table S1).

### Concordance of imputed genotypes

In an attempt to examine the quality of imputation using 1000G in our study, we validated the genotypes imputed in our study by way of comparing the genotype frequencies at the imputed markers with those genotyped in populations from the region as reported in either our in-house Kuwaiti Arab Exome Variant database (John et al. 2018) or in the Greater Middle East (GME) Variome Project (Scott et al. 2016) (which presents GME genetic variations). Our in-house exome variant database, derived from 291 samples, contained 5864 imputed markers (with a frequency ≥ 5% and Rsq ≥ 0.5), of which only five (0.09%) showed significantly different genotype frequencies (Fisher’s exact test, *p* adjusted ≤ 0.05). All five variants were either multiallelic or indels. Similarly, the GME database contained 8550 imputed markers (with a frequency ≥ 5% and imputation quality of Rsq ≥ 0.5). Of them, only 62 imputed variants (0.73%) showed significantly different genotype frequencies (Fisher’s exact test, *p* adjusted ≤ 0.05). Interestingly, these included 33 variants located in the major histocompatibility complex region, which is known to exhibit poor imputation quality (Matzaraki et al. 2017). The remaining 29 variants were either multiallelic or indels. It is also possible that the higher fraction of inconsistency observed with GME can be attributed to population differences within the Greater Middle Eastern region. These observations indicate that the quality of imputation using 1000G is good and is in agreement with genotyped data from Kuwait and the region. We removed all variants that showed significant differences in genotype distribution from further analyses.

### Summary of associations observed between metabolic traits and imputed variants

Statistical analyses of the associations between the variants (genotyped and imputed) and the 13 metabolic traits identified 978 unique associations involving 821 unique variants from 251 gene loci (Supplementary Dataset S1). Variants from 237 of the 798 association signals reported in Supplementary Dataset S1 exhibited higher effect allele frequencies in Arabs as compared in Europeans and the difference was statistically significant as per proportional test for comparison; of these 237 signals, 172 (73%) were not seen replicated in global GWA studies. When the association signals were not at a level of genome-wide significance or at least borderline to genome-wide significance, they only involved the established variants (annotated in the GWAS Catalog for traits relating to metabolic processes). Of these 821 variants, (i) 70 variants (from nine genes) formed 72 associations at genome-wide significance (p value < 5.0 × 10^−08^), (ii) 440 variants (from 76 genes) formed 455 associations of borderline to genome-wide significance (*p* value < 1.0 × 10^−06^ and > 5.0 × 10^−08^), and (iii) 319 variants (from 181 genes) formed 451 established associations (reported in the GWAS Catalog for the 313 traits relating to the 13 study-specific metabolic traits) at p values of suggestive evidence of association (> 1 × 10^−06^ and ≤ 0.05). LD pruning at *r*^2^ = 0.10 suggested nine LD-independent variants (two variants of genome-wide, two variants of borderline, and five variants of suggestive associations) among 821 variants. Table 2 presents the overall summary statistics of the extent of associations observed between variants and the tested metabolic traits, along with comparisons with the established 7668 SNP associations from the GWAS Catalog (see “Methods” for the protocols used to search the GWAS Catalog with search terms relating to metabolic traits and disorders, as listed in Supplementary Table S2).

**Table 2:** Summary statistics on observed associations between variants (genotyped and imputed) and the tested 13 metabolic traits. Data from comparing these associations with the 7668 established SNP associations from GWAS Catalog (as accessed in February 2019) is also included

Trait	Variants associating with the tested metabolic traits in meta-analysis with *p* values ≤ 0.05^#^	Number of variants retained from the previous column after removing those with *p* values > E−06 and not seen as established marker in GWAS Catalog^@^	Number of variants associating at genome-wide significance (*p* value ≤ 5E−08)^&^	Number of variants associating at borderline *p* values (> 5E−08 to < 1E−06)	Number of unique variants associating at suggestive *p* values and also found associated in GWAS Catalog with any metabolic-traits (against any search term used)	Number of unique variants found associated in GWAS Catalog with “Indirect^%^” metabolic traits (*p* value: S = at suggestive p value; B = at borderline *p* value; *G* = at genome-wide *p* value	Number of unique variants found associated in GWAS Catalog with the same trait (“direct” associations)
BMI	7664	58	0	16	42	13 (S)	13 (S)
Weight	16,706	38	0	0	38	22 (S)	2 (S)
WC	7725	65	0	23	42	30(S)	4 (S)
Height	5440	71	0	47	24	15 (S, B)	13 (S, B)
SBP	*7949*	74	3	61	10	0	1 (S)
DBP	*6703*	39	2	14	23	1 (S)	0
FPG	*7246*	13	2	14	25	6	1 (S)
HbA1c	*1807*	5	0	2	3	2 (S)	1
HDL	6838	153	63	57	33	14 (S, B, G)	13 (S, B, G)
LDL	6192	79	1	30	48	21 (S)	8 (S)
TG	8592	236	1	160	75	42 (S, B)	28 (B, S)
TC	6345	60	0	17	43	21 (S, B)	12 (S)
non-HDL	5984	59	0	14	45	27 (B, S)	–
Unique variants		821	70	440	319	163	83
Unique associations		978	72	455	451	214	96

^**#**^Considered are only those variants that are seen common between the two imputed data sets and are with MAF > 0.05 and HWE > 1E−05 and imputation mean score ≥ 0.5

^**@**^The following filter was applied on the entries from the previous column: While considering the associations resulting at suggestive *p* values, only those associations that involve established markers (annotated as associated with any traits relating to metabolic processes in GWAS Catalog) were considered

^**$**^“Broad” indicates any metabolic trait used as a search term to probe GWAS Catalog

^**%**^“Specific”: indicates traits within classes of anthropometry & obesity, blood pressure and hypertension, lipid profile, Glycemia and diabetes, lipid profiles, and cardiometabolic phenotypes (see Supplementary Table S2)

^&^The gene loci found associated at genome-wide significance were: *INTS10-LPL* (rs10635970-LDL), *LOC105377613-LOC105377614* (rs11132637, rs35387314, rs6846011, rs34453299, rs561984720, rs9790417, rs11942078, rs35668405, rs35602882, rs35266160, rs11721957, rs35466621, rs35699925, rs34329457, rs13147669, rs76018028, rs35192598, rs55944922, rs13141596, rs13120111, rs13119288, rs36021514, rs34728227, rs35127507, rs34706863-HDL), *CETP* (rs289713, rs7499892, rs11076175, rs200751500, rs12720908, rs12720922, rs17231569, rs11508026, rs7203984, rs12720926, rs5817082, rs1864163, rs34620476, rs34145065, rs708272, rs711752, rs3816117, rs1800775, rs17231506, rs36229491, rs821840, rs3764261, rs12149545 rs201825234, rs6499862, rs6499861, rs183130-HDL), *HERPUD1-CETP* (rs247617, rs247616, rs173539, rs56228609, rs56156922, rs12446515, rs7205692, rs7203286, rs11862052-HDL), *LOC105377614* (rs67294765, rs11736173-HDL), *BUD13* (rs66505542-TG), *CSMD1* (rs7838666-FPG), *RTN4* (rs2920844, rs2968781-DBP,SBP), DYRK1A-*LOC105372798* (rs2835788-SBP)

### Associations observed at genome-wide significance in meta-analysis

As mentioned above, 70 unique variants (15 established risk variants and 55 novel risk variants) from nine gene loci (seven genomic regions) were found to be associated at a genome-wide significant *p* value. Of them, 63 were associated with high-density lipoprotein (HDL), one with triglycerides (TGs), one with low-density lipoprotein (LDL), three with systolic blood pressure (SBP), and two with each of fasting plasma glucose (FPG) and diastolic blood pressure (DBP). The associated gene loci are listed in the footnote to Table 2. SNP quality information of these top variants is presented in Supplementary Table S3. These 70 variants contributed to 72 associations with the traits (Supplementary Table S4). The quantile–quantile plots depicting the expected and observed − log10 (*p* values) of all variant associations for all the 13 traits for the two cohorts and for the meta-analysis along with the values for genomic inflation (λ) are presented in Supplementary Figure S3. Manhattan plots depicting the − log_10_(*p *values) from the GWAS for all the traits in our meta-analysis are presented in Supplementary Figure S4. Regional association plots for regions of 500 Kb centered at the risk variants associating with the metabolic traits at genome-wide significance are presented in Supplementary Figure S5.

### Fine-mapping analysis

Fine-mapping analysis of the seven identified genomic regions revealed 95% credible causal variants for nine SNP-trait association signals, corresponding to eight lead SNPs. Table 3 presents a detailed list of 95% credible causal variants along with their association statistics. Among these eight lead SNPs giving credible sets, three (rs1864163-HDL, rs7838666-FPG, and rs2835788-SBP) showed a posterior inclusion probability (PIP) ≥ 0.5, another three (rs112861901 for HDL, rs66505542 for TG, and rs2920844 for SBP and DBP), showed PIP > 0.3 to < 0.5; and the remaining two lead SNPs (rs76018028-HDL and rs10635970-LDL) showed a very low PIP.

**Table 3:** The set of 95% credible causal variants (representing 8 lead SNPs) for the 72 SNP-trait association signals identified at genome-wide significance (refer to Supplementary Table S4)

Indexed SNP_effect allele, chromosomal position	Trait	Source	Effect allele frequency in Kuwait (EAF_KWT_)	Effect size^$^	*p* value	Gene, function consequence	95% credible SNPs (PIP) from fine mapping	Regional SNP heritability in 95% CI, mean ± SD	Effect allele frequency in European population (EAF_EUR_) (and adjusted *p* value as deduced from proportional test)
rs112861901_C, 8:19,750,951	HDL	OE^imputed^	0.11	0.2824	1.33E−04	INTS10, LPL; intergenic	rs112861901 (0.37), rs527775719 (0.14), rs77829308 (0.079), rs12550077 (0.064), rs148364036 (0.062), rs74549553 (0.048), rs79341217 (0.036), rs74974359 (0.034), rs10503666 (0.026), rs76085257 (0.026), rs4922113 (0.019), rs17482310 (0.018), rs79396901 (0.018), rs17410407 (0.0147)	0.008 ± 0.003	0.173 (0.0002)
		CM^imputed^	0.107	0.2434	8.54E−05				
		Meta	0.109	5.4790	4.28E−08				
rs76018028_C, 4:190,161,531	HDL	OE^imputed^	0.227	− 0.2082	3.65E−04	LOC105377613, LOC105377614; intergenic	rs76018028(0.046), rs55944922(0.045), rs36021514 (0.044), rs35192598 (0.044), rs34728227 (0.044), rs13120111 (0.043), rs34706863(0.043), rs35127507 (0.042), rs13141596 (0.042), rs13147669 (0.040), rs35699925 (0.038), rs34329457 (0.038), rs35466621 (0.037), rs13119288 (0.036), rs11721957 (0.032), rs35266160 (0.032), rs35602882 (0.025), rs6846011 (0.024), rs35668405 (0.023), rs34453299 (0.022), rs35387314 (0.022), rs561984720 (0.019), rs11132637 (0.019), rs9790417 (0.016), rs11735474 (0.0138), rs35398404 (0.0109), rs11723378 (0.011)	0.0104 ± 0.0036	0.288 (0.014)
		CM^imputed^	0.236	− 0.2605	7.69E−06				
		Meta	0.232	− 5.7040	1.17E−08				
rs1864163_A, 16:56,997,233^#^	HDL	OE^imputed^	0.25	− 0.2494	2.63E−06	CETP, intronic	rs1864163 (0.897), rs5817082 (0.046), rs11076175 (0.029)	0.025 ± 0.0056	0.270 (0.963)
		CM^genotyped^	0.286	− 0.2285	1.92E−08				
		Meta	0.268	− 6.3650	1.95E−10				
rs10635970_TAA, 8:19,745,039	LDL	OE^imputed^	0.497	− 0.1675	1.29E−04	INTS10, LPL; intergenic	rs11385353 (0.14), rs11995314 (0.08), rs7822518 (0.05), rs1441776 (0.04), rs11204080 (0.04), rs28522810 (0.035), rs1441775 (0.035), rs7828612 (0.033), rs34955499 (0.031), rs11204081 (0.03), rs966773 (0.018), rs12679834 (0.016), rs314 (0.016), rs10101204 (0.015), rs28675909 (0.015), rs12678604 (0.015), rs1031046 (0.014), rs12678603 (0.013), rs7844579 (0.013), rs10096633 (0.012), rs35550741 (0.01), rs72575589 (0.01)	0.01 ± 0.004	0.505 (0.919)
		CM^imputed^	0.504	− 0.1604	4.31E−05				
		Meta	0.5	− 5.6020	2.12E−08				
rs66505542_T, 11:116,623,213	TG	OE^imputed^	0.746	− 0.1751	3.27E−04	BUD13; intronic	rs66505542 (0.32), rs2266788 (0.08), rs3741298 (0.06), rs964184 (0.06), rs10750096 (0.031), rs1558861 (0.03), rs2072560 (0.03), rs9326246 (0.03), rs529068927 (0.027), rs2160669 (0.025), rs662799 (0.023), rs7123666 (0.02), rs2075290 (0.015), rs6589569 (0.014), rs7930786 (0.014), rs651821 (0.014), rs11216140 (0.014), rs1558860 (0.013), rs1974718 (0.012), rs7483863 (0.012), rs6589566 (0.012), rs9666150 (0.011), rs10790162 (0.011), rs9667814 (0.01), rs6589567 (0.01), rs4938313 (0.01), rs3825041 (0.01), rs6589565 (0.01)	0.017 ± 0.004	0.819 (2.25E-05)
		CM^imputed^	0.717	− 0.1832	9.41E−06				
		Meta	0.731	− 5.6920	1.26E−08				
rs7838666_C, 8:4,126,701	FPG	OE^imputed^	0.689	0.236	3.92E−05	CSMD1, intronic	rs7838666 (0.87), rs78686130 (0.043), rs7004794 (0.017), rs6999771 (0.016), rs80137543 (0.013)	0.01 ± 0.003	0.657 (0.251)
		CM^imputed^	0.685	0.254	4.47E−06				
		Meta	0.68	6.159	7.31E−10				
rs2920844_T, 2:55,341,367	DBP	OE^imputed^	0.860	0.184	2.08E−03	RTN4, upstream	rs2920844 (0.45), rs2968781 (0.45), rs56181849 (0.015), rs62138262 (0.014), rs56377199 (0.013)	0.009 ± 0.003	0.877 (0.224)
		CM^imputed^	0.846	0.272	1.50E−06				
		Meta	0.852	5.617	1.94E−08				
rs2920844_T, 2:55,341,367	SBP	OE^imputed^	0.860	0.202	5.32E−04	RTN4, upstream	rs2920844 (0.41), rs2968781 (0.41), rs56181849 (0.034), rs56377199 (0.032), rs62138262 (0.032)	0.011 ± 0.003	0.877 (0.224)
		CM^imputed^	0.846	0.257	5.37E−06				
		Meta	0.852	5.69	1.27E−08				
rs2835788_G, 21:38,906,071	SBP	OE^imputed^	0.120	0.241	1.91E−03	DYRK1A, LOC105372798, intergenic	rs2835788 (0.79), rs2835799 (0.16)	0.011 ± 0.003	0.128 (0.707)
		CM^imputed^	0.118	0.316	2.51E−06				
		Meta	0.119	5.559	2.72E−08				

^**$**^The effect size in the cases of CM and OM association studies denotes the resulting regression coefficient (beta) from the fit; and in the case of Meta-analysis denotes *Z*-score from *Z*-statistics

### SNP heritability analysis

Regional SNP heritability analysis explained an estimated variance of 2.5% in HDL for the 1-Mb region comprising rs1864163, 1.7% in TGs for the region comprising rs66505542, 1.4% in HDL for the region comprising rs76018028, and 1.1% in SBP for the region comprising rs2835788. All the remaining top signals showed ≤ 1% trait variance (Table 3).

### Association of novel variants in our meta-analysis with metabolic traits

The meta-analysis identified 472 unique novel SNP variants (from 76 genes) in our study cohort that were associated with one or more of the tested 13 metabolic traits, either at genome-wide or borderline to genome-wide significance. While none of these 472 variants were associated with any of the 313 traits relating to metabolic processes in the GWAS Catalog, polymorphisms from 57/76 genes were associated with metabolic traits (see Supplementary Dataset S1). Literature evidence supported the involvement of eight additional genes in metabolic processes (see Supplementary Dataset S1). Thus, the evidence supported a role in metabolic processes for 65/76 genes, while 11 genes (mostly uncharacterized) showed no apparent link to metabolic traits.

### Observed association signals and consistency in traits associated in GWAS Catalog

**Fig. 2:**
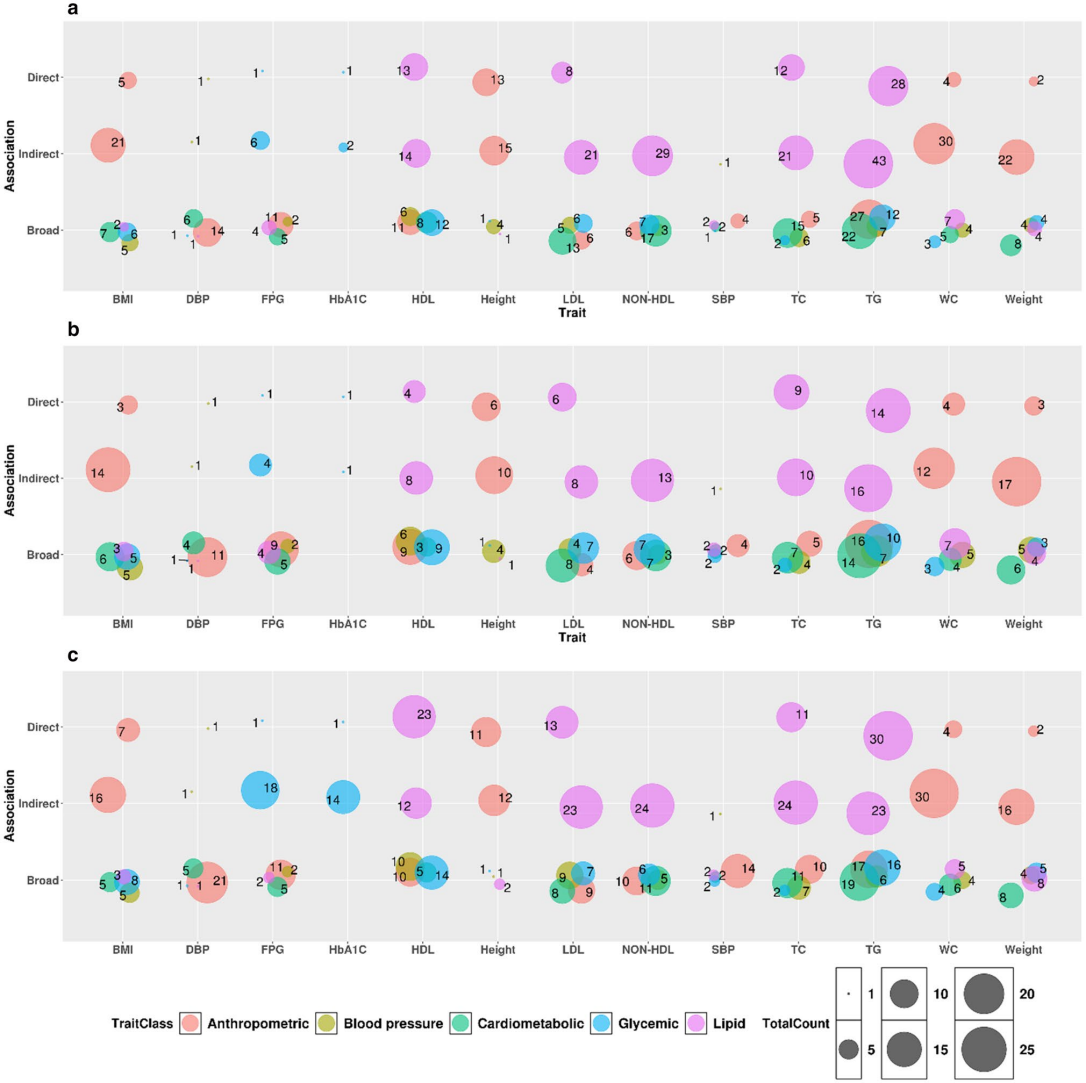
Bubble plots illustrating the distributions of observed established variants (**a**) and gene loci (**b**) associated with the study-specific metabolic traits onto types (direct, indirect or broad) of relationships with observed traits in GWAS Catalog (as accessed in February 2019). (**c**) Presents the count of global studies (publications) reporting the observed associations in GWAS Catalog for each of the ‘direct’, ‘indirect’, and ‘broad’ categories. 83 variants (from 42 genes) formed the ‘direct’ category; such variants were associated with 11 traits (namely Weight, height, WC, BMI, TC, TG, LDL, HDL, FPG, HbA1C and DBP). The count of unique variants per each of these 11 traits were: weight:2 (both at suggestive *p* values); height:13 (1 variant at borderline *p* value and 12 at suggestive *p* value); WC:4 (all at suggestive *p* values); BMI:5 (all at suggestive *p* values); TC:12 (1 variant at borderline *p* value and 11 at suggestive *p* value); TG:28 (9 at borderline p value and 19 at suggestive *p* value); LDL:8 (8 at suggestive *p* value); HDL:13 (9 variants at genome-wide significant *p* values, 2 at borderline *p* value and 2 at suggestive *p* value); DBP:1 (at suggestive *p* values); FPG:1 (at suggestive *p* value); and HbA1C:1 (at suggestive *p* value)

Trait consistency in association signals with the GWAS Catalog was demonstrated in 83 variants (from 42 genes), comprising the direct category (see “Methods”). Such variants were associated with 11 traits (namely, height, fasting plasma glucose (FPG), glycated hemoglobin (HbA1c), DBP, weight, waist circumference [WC], total cholesterol [TC], TG, LDL, body mass index [BMI], and HDL). For the traits of non-HDL and SBP, we did not find any direct match in the GWAS Catalog, but we did find indirect matches (Fig. 2). However, a vast majority of these signals were only at suggestive p values in our cohort (only nine signals were at a genome-wide significance and 12 at a borderline to genome-wide significance).

### Discovered association signals and overlap with broad classes of metabolic traits in GWAS Catalog

Interestingly, many of the variants that were found associated with the 13 study-specific metabolic traits in our cohort exhibited associations with members of the broad metabolic trait classes in the GWAS Catalog (see Fig. 2). The complete dataset of all the variant associations, with grouping and trait classes, is provided in Supplementary Dataset S2. The extent of the overlap in trait associations among the different classes of metabolic traits were as follows: (i) Among the variants that were associated with anthropometric traits in our cohort, 9 variants (7 genes) were also associated with lipid traits, 11 variants (eight genes) with cardiometabolic phenotypes, 11 variants (10 genes) with blood pressure traits, and 7 variants (6 genes with glycemic traits in the GWAS Catalog. (ii) Among the variants associated with lipid traits in our cohort, 48 variants (32 genes) were also associated with anthropometric traits, 22 variants (18 genes) with blood pressure traits, 48 variants (25 genes) with cardiometabolic phenotypes, and 31 variants (26 genes) with glycemic traits in the GWAS Catalog. (iii) Among the variants associated with glycemic traits in our cohort, 11 variants (9 genes) were associated with anthropometric traits, 2 variants (2 genes) with blood pressure traits, four variants (4 genes) with lipid traits, and 5 variants (5 genes) with cardiometabolic phenotypes in the GWAS Catalog. (iv) Among the variants associated with blood pressure traits in our cohort, 17 variants (14 genes) were associated with anthropometric traits, 2 variants (2 genes) with lipid traits, 2 variants (2 genes) with glycemic traits, and 7 variants (5 genes) with cardiometabolic phenotypes. The associated variants and genes shared with the different classes of metabolic traits are illustrated as Venn diagrams in Supplementary Figure S6 (for variants) and Supplementary Figure S7 (for genes). The associations comprising the broad category may illustrate that rather than trait inconsistency, pleiotropy and a common genetic basis exists among different classes of metabolic traits.

### Transferability of established association signals for metabolic traits to the Arab population

**Fig. 3:**
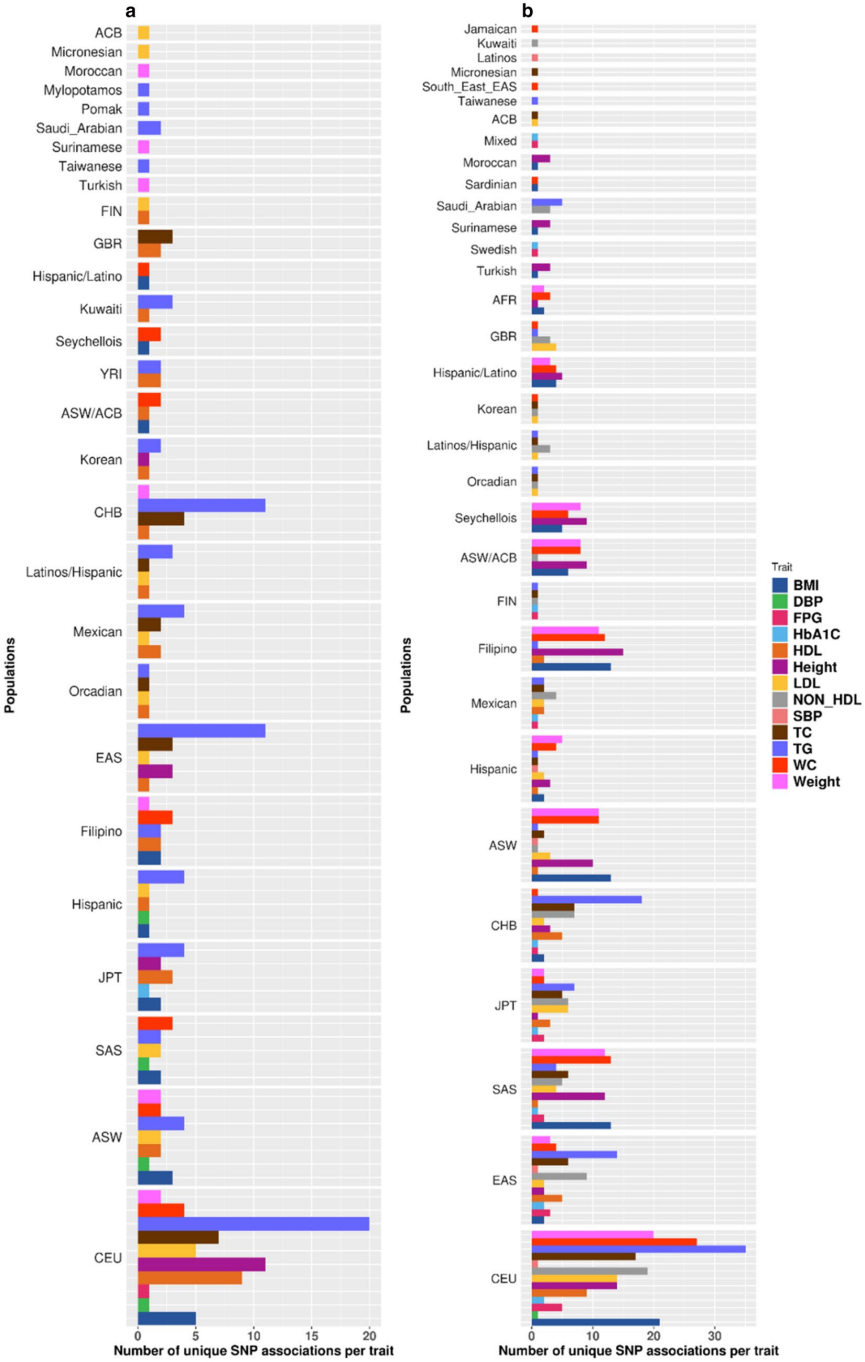
Ethnic transferability of SNP association signals for metabolic traits among populations. The figure presents the counts of transferable association signals (at the level of variants) from each global population to Arab population through ‘Direct’  (**a**) or ‘Indirect’  (**b**) relationships

We observed 349 unique SNPs (from 187 genes) established in global populations as associated with metabolic traits replicated in the Arab population through direct, indirect, or broad relationships. Of these unique variants, 83 (from 42 genes) were replicated through a relationship of direct association, 164 (from 77 genes) through indirect association, and 203 (from 131 genes) through broad association. The counts of transferable association signals at such variants from each global population to the Arab population are presented in Fig. 3, and similar counts at the gene level are presented in Supplementary Figure S8. The presented association signals were generally well illustrated in various studies (see Fig. 2) and in multiple global populations. Table 4 shows populations illustrating the replicability of associations from the direct relationship class. For example, the replicability of associations from the direct class for the traits of BMI, TG, and height has been observed in many populations, with BMI associations in Northern and Western European (CEU), East Asian (EAS), South Asian (SAS), Hispanic, Filipino, and Seychellois populations; TG associations in CEU, EAS, and Han Chinese in Beijing, China (CHB), populations; and height associations in CEU and EAS populations.

**Table 4:** List of established metabolic trait variants and genes replicating in the Kuwaiti population through the class of direct relationship

Trait	Population	SNP	Gene
Anthropometric	Turkish	rs4330912	LOC107985208, LOC107985210
	Americans of African Ancestry in SW USA (ASW)	rs10783050, rs11165643, rs2867125, rs1926872, rs2274432, rs1973993, rs4330912	EEF1A1P11-LOC105378866, LOC105373352-TMEM18, LOC105378866-RN7SL831P, COLGALT2, TSEN15, LOC107985208, LOC107985210
	Filipino	rs11165643, rs2867125, rs1926872, rs2274432, rs6755502, rs4330912	EEF1A1P11-LOC105378866, LOC105373352-TMEM18, COLGALT2, TSEN15, LOC107985208, LOC107985210
	Seychellois	rs11165643, rs2274432, rs6755502	EEF1A1P11-LOC105378866, LOC105373352-TMEM18, TSEN15
	Japanese in Tokyo, Japan (JPT)	rs11165643, rs939584, rs3791679, rs798497	EEF1A1P11-LOC105378866, LOC105373352-TMEM18, AMZ1, EFEMP1, GNA12
	Moroccan	rs4330912	LOC107985208, LOC107985210
	Americans of African Ancestry in SW USA or African Caribbeans in Barbados (ASW/ACB)	rs11165643, rs2274432, rs6755502	EEF1A1P11-LOC105378866, LOC105373352-TMEM18, TSEN15
	Hispanic/Latinos	rs11165643, rs2274432	EEF1A1P11-LOC105378866, TSEN15
	Hispanic	rs11165643	EEF1A1P11-LOC105378866
	Utah Residents (CEPH) with Northern and Western European Ancestry (CEU)	rs10783050, rs11165643, rs1555543, rs2867125, rs939584, rs1182188, rs1492820, rs1812175, rs3791675, rs3791679, rs4240326, rs6854783, rs7689420, rs798489, rs798497, rs798544, rs1926872, rs2266788, rs2274432, rs6755502, rs1973993, rs4330912	EEF1A1P11-LOC105378866, LOC105373352-TMEM18, LOC105378866-RN7SL831P, AMZ1, EFEMP1, GNA12, HHIP, HSPD1P5-ANAPC10, APOA5, COLGALT2, TSEN15, LOC107985208, LOC107985210
	South Asian (SAS)	rs11165643, rs2867125, rs1926872, rs2274432, rs6755502	EEF1A1P11-LOC105378866, LOC105373352-TMEM18, COLGALT2, TSEN15
	Surinamese	rs4330912	LOC107985208, LOC107985210
	Korean	rs3791675	EFEMP1
	East Asian (EAS)	rs1415701, rs3791675, rs6845999	EFEMP1, HHIP, L3MBTL3
Blood pressure	South Asian (SAS)	rs7692387	GUCY1A3
	Hispanic	rs7692387	GUCY1A3
	Utah Residents (CEPH) with Northern and Western European Ancestry (CEU)	rs7692387	GUCY1A3
	Americans of African Ancestry in SW USA (ASW)	rs7692387	GUCY1A3
Lipid	Kuwaiti	rs1864163, rs11654954, rs2934952, rs9972882	CETP, LOC105371770-LOC105371771, PGAP3, STARD3
	British in England and Scotland (GBR)	rs15285, rs3764261, rs12740374, rs672889, rs71435601	CETP, LPL, APOB-TDRD15, CELSR2, TDRD15-LOC105374317
	Korean	rs3764261, rs651821, rs6589566	CETP, ZNF259
	Americans of African Ancestry in SW USA (ASW)	rs247617, rs3764261, rs12713956, rs12740374, rs4665972, rs6589566, rs780094, rs964184	CETP, HERPUD1, APOB, CELSR2, GCKR, SNX17, ZNF259
	Mylopotamos	rs964184	ZNF259
	Utah Residents (CEPH) with Northern and Western European Ancestry (CEU)	rs1532624, rs17231506, rs173539, rs1800775, rs183130, rs1864163, rs2489279, rs3764261, rs7499892, rs12740374, rs515135, rs629301, rs646776, rs660240, rs10401969, rs2980869, rs562338, rs599839, rs7528419, rs10889353, rs1260326, rs1558861, rs17321515, rs174546, rs174547, rs174550, rs1748195, rs2160669, rs2266788, rs2954022, rs2954029, rs2954031, rs3761445, rs662799, rs780093, rs780094, rs964184, rs995000	CETP, HERPUD1, ZNF648, APOB-TDRD15, CELSR2, CELSR2-PSRC1, APOB, LOC100287183, SUGP1, TRIB1-LOC105375746, APOA4, APOA5, DOCK7, FADS1, GCKR, PLA2G6-MAFF, RP11-136O12.2, ZNF259
	South Asian (SAS)	rs515135, rs660240, rs1558861, rs2954029	APOB-TDRD15, CELSR2, APOA4, APOA5, TRIB1-LOC105375746
	East Asian (EAS)	rs3764261, rs2738452, rs10401969, rs2980869, rs599839, rs1260326, rs174550, rs2160669, rs2954021, rs2954022, rs2954029, rs3761445, rs662799, rs780094, rs995000	CETP, LDLR, CELSR2-PSRC1, SUGP1, TRIB1-LOC105375746, DOCK7, FADS1, GCKR, PLA2G6-MAFF, ZNF259
	Pomak	rs964184	ZNF259
	Yoruba in Ibadan, Nigeria (YRI)	rs1800775, rs183130, rs662799, rs964184	CETP, ZNF259
	Han Chinese in Beijing, China (CHB)	rs3764261, rs10401969, rs2980869, rs599839, rs6871667, rs12042319, rs1260326, rs174550, rs2954022, rs2954029, rs3761445, rs651821, rs662799, rs780094, rs995000	CETP, ANKRD31-HMGCR, CELSR2-PSRC1, SUGP1, TRIB1-LOC105375746, DOCK7, FADS1, GCKR, PLA2G6-MAFF, ZNF259
	Filipino	rs1800775, rs183130, rs662799, rs964184	CETP, ZNF259
	Saudi Arab	rs1558861, rs964184	APOA4, APOA5, ZNF259
	Americans of African Ancestry in SW USA or African Caribbeans in Barbados (ASW/ACB)	rs3764261	CETP
	Hispanic	rs247617, rs12713956, rs4665972, rs6589566, rs780094, rs964184	CETP, HERPUD1, APOB, GCKR, SNX17, ZNF259
	Finnish in Finland (FIN)	rs3764261, rs646776	CETP, CELSR2-PSRC1
	Micronesian	rs7703051	ANKRD31-HMGCR
	Taiwanese	rs662799	ZNF259
	Orcadian	rs1532624, rs646776, rs780094	CETP, CELSR2-PSRC1, GCKR
	Hispanic/Latinos	rs7499892, rs660240, rs7528419, rs2954031, rs780093, rs964184	CETP, CELSR2, GCKR, RP11-136O12.2, ZNF259
	Mexican Ancestry from Los Angeles USA (MXL)	rs1532624, rs7499892, rs660240, rs3902354, rs7528419, rs1558861, rs2954031, rs780093, rs964184	CETP, CELSR2, CELSR2-PSRC1, APOA4, APOA5, GCKR, RP11-136O12.2, ZNF259
	Japanese in Tokyo, Japan (JPT)	rs3764261, rs56156922, rs9939224, rs10889353, rs1260326, rs2954029, rs651821	CETP, HERPUD1, DOCK7, GCKR, TRIB1-LOC105375746, ZNF259
Glycemic	Utah residents (CEPH) with Northern and Western European Ancestry (CEU)	rs10885409	TCF7L2
	Japanese in Tokyo, Japan (JPT)	rs12219514	HHEX-EXOC6

### Effect size and replicability of established variants

**Fig. 4:**
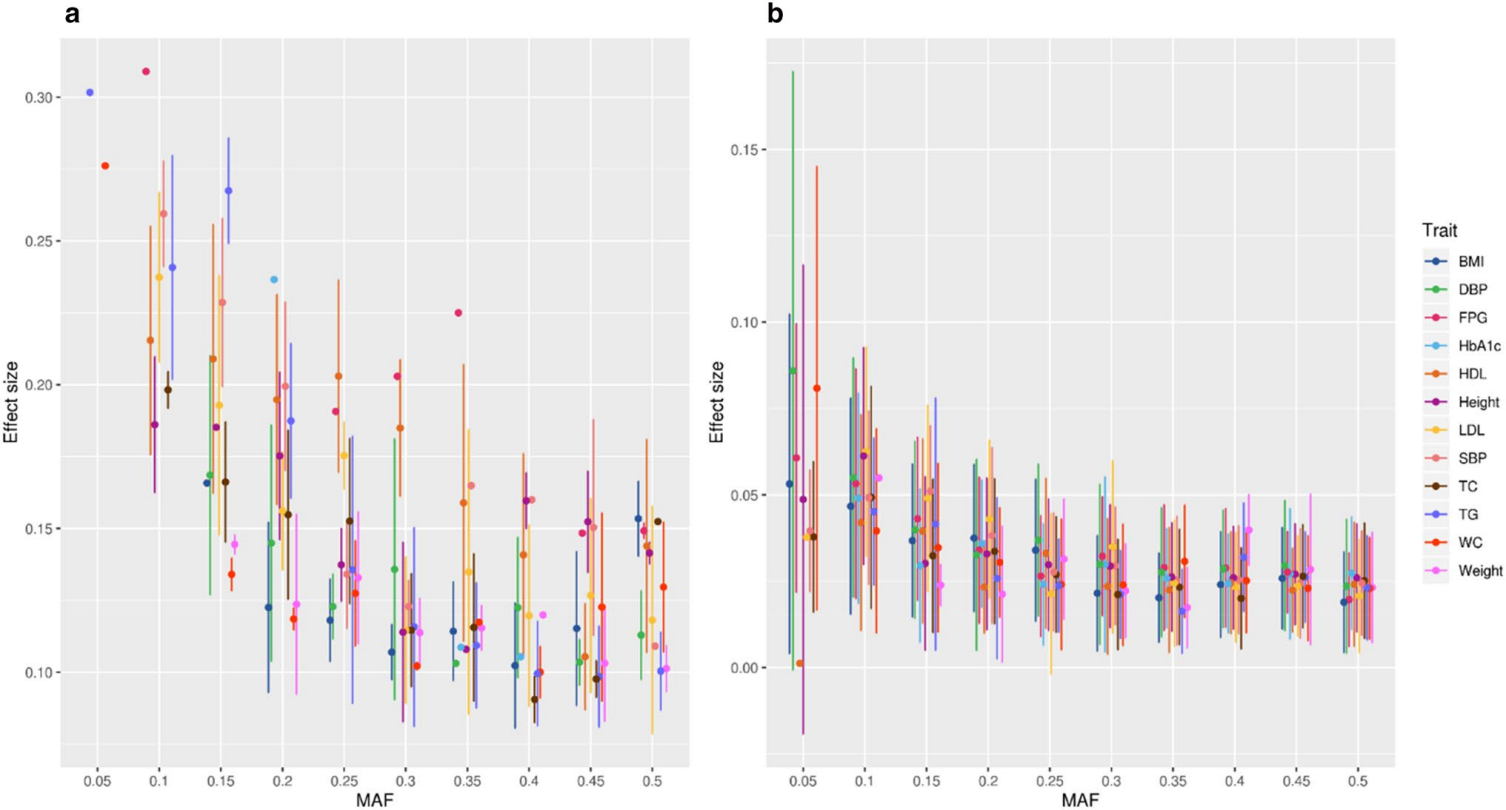
Trends in the mean of effect sizes of the established variants replicating  (**a**) in our study and those that are not replicating  (**b**). For every 5% incremental MAF of replicating variants (at borderline and suggestive p values) and non-replicating variants, we formed bins of effect size (beta) and then calculated summary statistics (mean ± standard deviation [SD]) for each bin for each of the 13 traits. Note the differences in the scaling of *Y* axis

Figure 4 presents the trend in effect sizes against increasing MAF values of the established variants that were seen replicating in our study cohort (Fig. 4a) and those that were not replicating in our study cohort (Fig. 4b). Study variants, replicating the established variants, showed a high effect size. On the other hand, non-replicating variants showed a low effect size.

### Estimation of sample size for replicating and non-replicating variants

The estimated power and sample size (see “Methods” for power calculation) for the variants of different effect sizes and MAFs for different traits are presented in Supplementary Figure S9 (the left panel presents the results of established variants that were replicated in our study, and the right panel presents the results of established variants that were not replicated in our study). The figure illustrates that most of the established variants that were seen replicating in our study would attain genome-wide significant p values (5.0 × 10^−8^) for most of the traits at 80% and even more power with a sample size of 10,000, while non-replicated markers would be less likely to replicate at genome-wide significant p values, even at a sample size of 20,000.

### List of gene loci implicated in metabolic processes by our meta-analysis

**Fig. 5:**
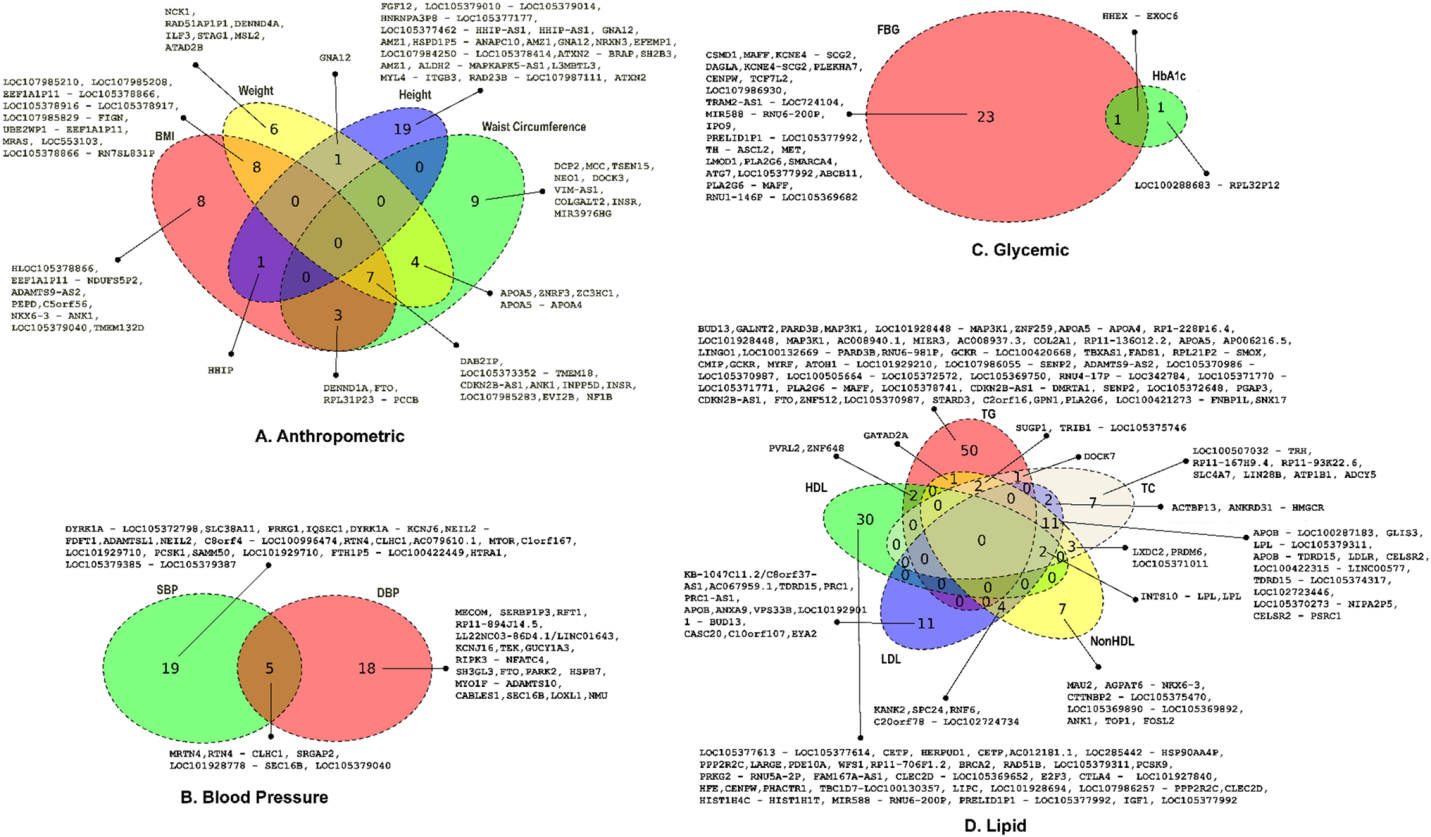
Venn diagrams showing genes associated with members of each of the four classes of tested 13 metabolic traits among the Kuwaiti population

This study established the association of 66 genes with anthropometric traits, 42 genes with blood pressure traits, 25 genes with glycemic traits, and 133 genes with lipid traits (Fig. 5) at different *p* value thresholds for the SNP-trait associations (genome-wide significance of < 5.0 × 10^−8^, borderline to genome-wide significance of < 1.0 × 10^−6^ and > 5.0 × 10^−8^, and suggestive p values of > 1.0 × 10^−6^). An association at a suggestive p value was only considered if the variant was also listed as associated with metabolic traits in the GWAS Catalog. There were 24 genes associated with multiple anthropometric traits, five with both SBP and DBP, one with both FPG and HbA1c, and 28 with multiple lipid traits. Many of these genes (9/66 associated with anthropometric traits, 25/42 associated with blood pressure traits, 20/25 associated with glycemic traits, and 101/133 associated with lipid traits) were also found associated with metabolic traits in the GWAS Catalog.

### Functional consequences of discovered variants

The distribution of the variants as per their functional consequences on gene structures as gathered (see “Methods”) is presented in Supplementary Figure S10A. Up to 52% of the variants were intronic, and up to 30% were intergenic. Up to 6% were located upstream of a gene. Approximately 3% of the variants were seen in regulatory regions, and approximately 1.5% of variants led to amino acid changes in the encoded proteins. The distribution of the variants as per their proximity to the transcription start sites (TSSs) is presented in Supplementary Figure S10B. The majority of the 821 study variants were situated close to the TSS, with 56 variants within a 1-Kb region from the TSS, and 107 variants within a 2-Kb region from the TSS.

### Functional prioritization of discovered variants

Analysis for functional prioritization of the variants led to valuable knowledge regarding the potential functional signatures around the study variants, and such signatures plausibly drive the course of downstream gene expression. As many as 752/821 variants could be ranked based on the functional niches. These 752 variants, along with their functional niches, are presented in Supplementary Dataset S3. Our study identified 33 of the top 100 ranked variants as novel risk variants. The top-ranked variants included the following: (i) the rs12740374 variant of the *CELSR2* gene (associating with non-HDL, LDL, and TC at p values borderline to genome-wide significance), which carried a high score for DNase hypersensitive sites (HSs), DNase footprint, and transcription factor binding sites (TFBS) and was ranked 1; (ii) the variant rs3749147 of *GPN1,ZNF512* (associating with TG at a suggestive *p* value), which overlapped with the promoter, located very close to the TSS, carried a high score for DNase HSs and TFBS, and was ranked 2; (iii) the variants rs7670 and rs4705745 of the *DCP2* gene (associating with WC at p values borderline to genome-wide significance), which carried high scores for DNase HSs and were ranked 3 and 62, respectively; and (iv) the variants rs62355943 and rs72758038 of the *MAP3K1* gene (associating with TG at *p* values borderline to genome-wide significance), which overlapped with the promoter and CpG shore, carried high scores for DNase HSs and TFBS, and were ranked 5 and 6, respectively.

### The discovered risk variants and their ability to regulate expression of genes

A functional assessment of the variants (and the genes harboring the variants) in terms of their ability to up- and/or downregulate genes (as deduced by examining eQTL’s from GTEx) is illustrated in Supplementary Figure S11 (A and B). Of the 821 identified study variants, 510 differentially regulated the expression of 464 genes across 49 tissues: 385 variants upregulated 291 genes, and 402 variants downregulated 294 genes (Supplementary Dataset S4). Among the 472 novel risk variants identified in the study, 283 altered the gene expression. Upon using the stringent criteria of considering only those eQTL’s with *q* value ≤ 0.05, 62 of the study variants were seen to differentially regulate the expression of 39 genes across 38 tissues; 34 variants upregulated 22 genes and 33 variants downregulated 19 genes (see sheet 2 of Supplementary Dataset S4 and Supplementary Figure S12).

## Discussion

Arabian Peninsula is at the nexus of Africa, Europe, and Asia and has been implied in early human migration route out of Africa and in early inter-continental trade routes. The post-oil rich era has seen a drastic shift from nomadic style of living to more sedentary lifestyle and rapid nutrition transition resulting in a dramatic increase in the prevalence of metabolic-related disorders. However, the global genetic studies for metabolic studies were mostly performed on European populations followed by African American, East Asian, and South Asian populations. Populations from Arabian Peninsula have not been included in these global studies and there is limited genetic association data for metabolic traits in Arab population. Close-kin marriage and large families are cultural factors in the region. The practice of consanguineous marriages and living in isolation by community expectedly leads to increased endogamy, homozygosity, and accumulation of deleterious recessive alleles in the gene pool. The Arab population has been vulnerable to a plague of recessive genetic disorders. Consanguinity and inbreeding can also play an important role in the etiology of complex disorders (Rudan et al. 2006). Given such a unique genetic profile, studying such an Arab population is expected to augment international efforts to identify genetic regulation of metabolic traits.

Although published GWAS have reported several thousand genotype associations with metabolic traits, they have mostly focused on people of European ancestry and, to an extent, East Asian ancestry, and it has remained unclear whether these findings could generalize to every other population. The frequencies of risk alleles associated with a range of traits in GWAS can differ substantially between continental populations (Adeyemo and Rotimi 2010). Thus, it is crucial to assess how well these associations can be extended to populations with different continental ancestry. An increasing number of studies aimed at generalizing these associations to various ethnic populations are being pursued globally (Adeyemo et al. 2012; Langlois et al. 2016; Liu et al. 2012; Lu and Loos 2013). Fine-mapping of the replicating risk gene loci is performed to identify the true causal variants (Liu et al. 2012; Mahajan et al. 2018; Wu et al. 2013). Determining the transferability of established risk loci and variants to different ethnic populations is essential for generalizing and reconfirming the findings from previous global collaborative studies as well as for enabling the successful transfer of the knowledge, research tools, resources, and drug targets generated by the global studies to a broad range of populations with different ethnicities.

This study performed genome-wide imputation of genotypes for variants that were untyped in the bead chips used in our previous GWAS, namely, the high-density OmniExpress BeadChip array (Hebbar et al. 2017a, 2018) and low-density Cardio-MetaboChip array (Hebbar et al. 2017b). An assessment of the imputation quality obtained in this study (based on the imputation quality score and the proportion of variants above the threshold for the quality score at every bin of MAF, incremented by 5%), suggested that the imputation quality was unprecedentedly good with common variants compared to low-frequency variants (MAF < 5%). Furthermore, it was observed that even using the low-density SNP array (Cardio-Metabo BeadChip), a mean Rsq of 0.75 could be obtained for common variants. Although the 1000G ALL panel was used as the reference panel for imputation, a high concordance was observed between the genotype frequencies of the imputed markers and their frequencies as obtained from the GME Variome Project (Scott et al. 2016) or from our in-house Arab Exome Variant database (John et al. 2018). This suggests that the imputation of both high-density and low-density SNP data using 1000G haplotypes as the reference panel successfully imputes common variants in the Arab population.

Our study identified 978 association signals (involving 821 variants from 251 genes) for the selected metabolic traits. Up to 95% of the 251 identified gene loci are supported by evidence from either annotation in the GWAS Catalog or from experimental studies reported in the literature (see Supplementary Dataset 1). We found 70 variants from nine gene loci associated with metabolic traits at a genome-wide significance; these gene loci include *BUD13*, *CETP*, *CSMD1*, *DYRK1A*, *HERPUD1*, *INTS10*, *LPL*, and *RTN4*, which are known to be involved in metabolic processes. Of them, 17/70 variants were established risk variants for metabolic traits and are listed in the GWAS Catalog. The genes harboring the remaining variants were associated with metabolic processes as seen in the GWAS Catalog or from the literature.

Our study inferred three of the observed risk variants (rs112861901/[*INTS10, LPL*] associated with HDL, rs1864163/*CETP* associated with HDL, and rs7838666/*CSMD1* associated with FPG) as causally implicated. Furthermore, the regional SNP heritability analysis demonstrated that the regions corresponding to the top signals showed low values, typically < 2%, except for the cholesteryl ester transfer protein (CETP) region (2.5%). These low values indicate that the proportion of metabolic trait variance that is attributable to these identified genetic variations is low among the study population. Although studies reporting a higher explained variance due to genetic variation in *CETP* exist [for example, Blauw et al. (2018) reported a high value of 16% for the explained variance in CETP concentration due to a set of 3 *CETP* variants], we cannot rule out the possibility of environmental interactions being responsible for larger relative contributions (Visscher et al. 2008) to the variance of traits in our study cohort.

The GWAS era has successfully associated thousands of genetic variants with risk for complex disorders and traits; for example, examination of the GWAS Catalog (accessed Feb 2019) found 7668 associated genetic variants for the 13 metabolic traits considered in this study. However, it still remains a challenge to translate these statistical associations to knowledge on how they functionally manifest to alter the biology underlying the disease risk (Edwards et al. 2013). A large part of this difficulty results from the fact that > 90% of disease-associated variants are located in non-protein coding regions of the genome (such as introns and intergenic regions) and not within the 1.5% coding part of the human genome (Hindorff et al. 2009; Maurano et al. 2012; Schaub et al. 2012). In line with these reports, we observed that up to 88% of the 821 study variants were located in introns or intergenic regions or upstream of genes as opposed to a mere 1.5% of the variants leading to amino acid changes in the encoded proteins. It has been proposed that many such variants located in non-protein coding regions might influence disease risk by way of altering the regulation of the target genes and they might form part of regulatory motifs (Gallagher and Chen-Plotkin 2018; Maurano et al. 2012; Schaub et al. 2012). The analysis of regulatory elements that we present in this study demonstrates that only around 3% of the 821 study variants were seen in regulatory motifs and that at the most 13% of the 821 study variants were seen within a 2-Kb region of TSSs. Variant prioritization based on the observed functional niches could uncover in our study a variety of regulatory signals around novel variants from genes such as *DCP2*, *MAP3K1*, *HHIP*, *KANK2*, *DAGLA*, *MAFF*, *CETP*, *LOC646576*, and *APOA5*. It is now established that GWAS hits for common diseases are enriched for eQTLs and that disease-associated risk variants are likely to regulate expression levels of target genes than would be expected by chance (Albert and Kruglyak 2015)**.** In line with these reports, we found that up to 510 (62%) of the 821 study variants were annotated in GTeX as regulating 464 genes in 49 tissues; further use of the criteria of *q* value ≤ 0.05 (a measure of significance in terms of false discovery rate) to filter the eQTL variants, reduced the number of such variants to 62 (7.6% of the 821 study variants) which differentially regulated expression of 39 genes in 38 tissues. Deciphering the functional role of the GWAS-associated risk variants is a challenge and requires comprehensive computational and experimental functional analysis studies.

It is well recognized that several genes have pleiotropic effects on multiple disorders, such as among T2D, obesity, and dyslipidemia (Chen et al. 2018); among obesity, cardiovascular disease outcomes, and cardiovascular risk factors (Rankinen et al. 2015); and among lipid metabolism and metabolic syndrome (Park et al. 2011). Our approach of using 7668 established variant associations for metabolic traits to classify the associations identified in our study aided in the identification of genetic loci with shared impacts on different metabolic processes. Some examples of such exemplary variants and genes include the following: (i) rs780093 of *GCKR* associated with TG shares associations with all five broad classes of metabolic traits (anthropometric, blood pressure, cardiometabolic, glycemic, and lipid traits); (ii) rs1260326 (*GCKR*), rs780094 (*GCKR*), and rs2925979 (*CMIP*) share associations with four broad classes of metabolic traits (anthropometric, cardiometabolic, glycemic, and lipid traits); and (iii) ten variants share associations with three broad classes of metabolic traits, including (a) rs6795735 (*ADAMTS9-AS2*) and rs6857 (*PVRL2*) with anthropometric, glycemic, and lipid classes; (b) rs15285 (*LPL*) with blood pressure, cardiometabolic, and lipid classes; (c) rs11977526 (*LOC102723446*) and rs198846 (*HIST1H1T*) with blood pressure, glycemic, and lipid classes; (d) rs2820315 (*LMOD1*) and rs564398 (*CDKN2B-AS1*) with anthropometric, cardiometabolic, and glycemic classes; (e) rs645040 (*RPL31P23-PCCB*) with anthropometric, cardiometabolic, and lipid classes; (f) rs11556924 (*ZC3HC1*) with anthropometric, blood pressure, and cardiometabolic classes; and (g) rs7248104 (*INSR*) with anthropometric, blood pressure, and lipid classes.

The replicability of GWAS-identified signals for consistency in allele frequencies and effect sizes has been assessed in different major continental populations (Haiman et al. 2012; Kraft et al. 2009; Li and Keating 2014; Marigorta and Navarro 2013; Marigorta et al. 2018; Ntzani et al. 2012). We, for the first time, evaluated the impact of effect sizes on the replicability of established risk variants in ethnic Arab populations. Our results confirm the common notion that large effect associations usually replicate well. Our estimation of the sample size for an Arab cohort required to replicate established associations at genome-wide significance revealed that even with a sample size of 10,000, the established variants that we found replicating in our study (at different *p* values) would attain genome-wide significance. Furthermore, even with a sample size of 20,000, the established variants that we found as non-replicating in our study were less likely to replicate at genome-wide significance.

Our previous publications using one or the other of the two study cohorts could identify risk variants at genome-wide significance only under genetic models based on recessive mode of inheritance. Examples include: the studies using the OmniExpress cohort identified a recessive marker from *RPS6KA1* associated with FPG (Hebbar et al. 2020) and 6 recessive markers from *RPS6KA1*, *LAD1*, *Or5v1*, *CTTNBP2-LSM8*, *PGAP3* and *RP11-191L9.4-CERK* associated with TG (Hebbar et al. 2018); and the study using the CardioMetabo cohort identified a recessive marker from *ZNF106* associated with HbA1c (Hebbar et al. 2017b). The only exception to identifying recessive markers is the study wherein we identified an additive marker from *TCN2* associated with WC using the OmniExpress cohort (Hebbar et al. 2017a). Adopting the approach used in the current study to perform meta-analysis on the summary statistics from both the cohorts led to identification of several additive markers as opposed to recessive markers at genome-wide significance—such as those from *LPL* (LDL), *CETP* (HDL), *BUD13* (TG), *CSMD1* (FPG), *RTN4* (DNP, SBP), *DYRK1A* (SBP). It is further the case that some of the additive markers that we identified at suggestive p values in our previous studies (Hebbar et al. 2017b) appeared in the current study at genome-wide significance as in the case of markers from *CETP* (HDL) and *BUD13* (TG). The ability to detect additive risk variants enabled us to explore the transferability of established markers (which are often identified through additive genetic model) to Arab population.

Ours study has certain limitations. (i) The association analysis considered only common variants (MAF ≥ 5%), and we set this criterion upon considering the small sizes of the datasets and the rapid degradation in imputation accuracy when low-frequency variants are considered. Thus, the transferability of low-frequency variants could not be examined in this study. However, we are considering “ROH-region-based multi-marker association tests in an attempt to improve analysis power” as follow-up to the current work. (ii) Because the two cohorts used in our study included both diabetic patients and healthy participants, the identified associations retained significance when the models were adjusted for the covariate of diabetes status. It is often the case that quantitative trait associations are performed on entirely non-diabetic participants or on entirely diabetic patients. In our earlier study (Hebbar et al. 2020), using one of the two cohorts mentioned here, we showed that 3/4 markers identified from the full cohort performed better in terms of retaining significance in the sub-cohort of entirely diabetic patients, and the fourth marker performed better in terms of retaining significance in the sub-cohort of participants free of diabetes. (iii) Although the Haplotype Reference Consortium (HRC) panel is more recent, we used the 1000G Project panel for imputation. There is no gain in HRC imputation in our study cohort as the HRC Release 1 (https://www.haplotype-reference-consortium.org/participating-cohorts) used by the MIS server does not contain Middle Eastern samples. The 1000G panel has been used more often in the literature, and it includes comparatively more diverse parental populations. However, the choice between HRC and 1000G panels does not matter, since our study considers only common variants. Furthermore, it has been demonstrated that in individuals of African ancestry, the 1000G panel also showed higher performance compared with the HRC panel in terms of the number of imputed variants with high accuracy (Vergara et al. 2018), and in Estonian individuals, the accuracy and sensitivity of imputation for common variants seemed to be similar between the 1000G and HRC panels (Mitt et al. 2017). Arab-specific haplotype reference panels are still under development in the region. (iv) The Cardio-Metabo BeadChip used to genotype one of the two cohorts is a fine-mapping array with much fewer markers as opposed to the OmniExpress BeadChip used to genotype the second cohort. As a result, there are much fewer genotyped and imputed SNPs resulting for the cohort using the Cardio-Metabo chip (at 1.52 million as opposed to 6.08 million obtained for the other cohort with OmniExpress BeadChip) leading to spotty coverage of the genome. (v) The study lacks formal pathway and gene-set analyses—it is our intention to perform these analyses as part of our future studies to translate the identified statistical associations to functional manifestations. (vi) While the values for genomic inflation factor (λ) were consistently close to 1.0 in the GWAS summary statistics from each of the two cohorts for each of the 13 traits, summary statistics from the meta-analysis exhibited slight inflation to the order of *λ* = 1.09 in the cases of HDL, SBP and Height. This can be a concern because of the moderate sample size of 2732. Considering that we have taken care of cryptic relatedness among samples in each of the two cohorts as well as between the cohorts, it is possible to attribute that these traits are probably associated with elevated polygenicity. (vii) The sample size was small. However, our study has succeeded in achieving its aim of finding causal variants and identifying potential novel hits specific to the Arab population.

In conclusion, this is the first presented study using an Arab population to perform genome-wide imputation and meta-analysis. We identified novel variants from seven established genes and two novel gene loci at genome-wide significance in the Kuwaiti population. We further identified 83 established SNP-metabolic trait association signals from the GWAS Catalog in their exact forms (the same variant associated with the same trait as in the GWAS Catalog) in the Kuwaiti population. We observed that of the identified association signals (for the four classes of anthropometric, blood pressure, lipid, and glycemic traits), a set of 330 SNPs (from 182 genes) were annotated in the GWAS Catalog as associated with other traits from the same class or from different classes of metabolic traits. These gene loci are indicative of a common genetic basis across the different metabolic processes. The replication of such established association signals provides further external validation and demonstrates the transethnic translatability of the risk loci associated with metabolic traits. The report conveys the following overall messages: the study (i) examined an understudied population characterized by inbreeding; (ii) demonstrated imputation from low density arrays to 1000 genomes haplotype reference panel; (iii) identified significant risk loci for metabolic traits, *albeit* previously known, in this novel population of Arabs; and (iv) indicated important metabolic pathways and genes.

## Methods

### Study participants

The study participants were selected from two independent cohorts recruited in our previous studies as part of two approved research projects at Dasman Diabetes Institute, Kuwait: (i) a cohort of 1965 samples resulting after QC steps based on genotype data (Hebbar et al. 2017b) and (ii) a cohort of 1913 samples resulting after QC steps (Hebbar et al. 2018). The participants were randomly recruited under protocols that were approved by the scientific and ethics advisory boards at Dasman Diabetes Institute. The study consists of two participant groups. The first group included a random representative sample of adults (> 18 years of age) of Arab ethnicity across the six governorates of the State of Kuwait. A stratified random sampling technique was used to select native Kuwaiti participants from the computerized register of the Public Authority of Civil Information, a government body that keeps and maintains personal information records of both Kuwaiti citizens and expatriates (including citizens of other Arab countries from the region). The second participant group comprised patients with diabetes or prediabetes seeking tertiary medical care in clinics at the Dasman Diabetes Institute, visitors to our nutrition programs and fitness center, visitors to our open day events (where various diagnostic screening services are offered), and visitors to our campaigns at primary health centers and blood banks in each of the six governorates of the State of Kuwait. As previously mentioned in our studies (Hebbar et al. 2017b, 2018), upon confirming that the participants had fasted overnight, they signed consent forms, blood samples were collected, vital signs were measured, and the nationality and ethnicity of each participant were confirmed via a rigorous questionnaire that addressed parental lineages up to three generations. Details of medications taken by the participants for lowering lipid levels, diabetes, and hypertension were collected and used in correction procedures with the association statistics. Data on illnesses (e.g., diabetes and cardiovascular complications) were also recorded. The guidelines of the Institutional Ethical Review Committee were followed for the proper collection of blood samples and the measurement of vital signs. All clinical assays for measurements of trait outcomes for both the cohorts were performed at a CAP (College of American Pathologists) accredited laboratory at Dasman Diabetes Institute.

## Genome-wide genotyping

We previously genotyped 1965 individuals of Arab ethnicity using the Illumina Human Cardio-Metabo BeadChip (MetaboChip) (Hebbar et al. 2017b) and 1913 individuals of Arab ethnicity using the Illumina Human OmniExpress BeadChip (OmniExpress) (Hebbar et al. 2017a, 2018). The protocols that were followed to perform genome-wide genotyping and QC are also described in these studies. We used PLINK 1.9 tools (Purcell et al. 2007) for data management and QC. Genotypes were converted to the National Center for Biotechnology Information’s Genome Reference Consortium Human Build 37 (hg19) to ensure consistent SNP phasing for each genotyping array. The genotyping QC procedures included the following: (i) samples with a call rate > 95% were retained; (ii) samples were checked against relatedness (both within each of the two cohorts and between the cohorts) and ancestry mismatch: one randomly selected representative of each set of related samples was retained, and samples with ancestry mismatch were removed; (iii) samples with heterozygosity > median + 3* interquartile range were excluded; and (iv) SNPs with a call rate > 98%, Hardy–Weinberg equilibrium (HWE) > 10^−6^, and MAF > 1% were retained. In a further QC step, samples that were common to both the study cohorts were removed from one of the sample sets.

### Phenotyping study participants for metabolic traits

For each participant, measurements were available for 13 quantitative metabolic traits from four classes: (i) anthropometry: height, weight, BMI, and WC; (ii) blood pressure: SBP and DBP; (iii) glycemia: FPG and HbA1c; and (iv) serum lipids: LDL, HDL, TC, TG, and non-HDL cholesterol (obtained by subtracting the HDL from the TC level).

### Imputation

Genotype imputation was performed on the MIS (Das et al. 2016) using the 1000G ALL reference panel from 1000G Project Phase 3 V5 for imputation, Eagle v2.3 for phasing, and Minimac3 as the algorithm for imputation (Loh et al. 2016). Prior to data submission to the MIS, the input data was checked against the 1000G reference SNP list using the HRC or 1000G Imputation Preparation and Checking Tool (https://www.well.ox.ac.uk/~wrayner/tools/HRC-1000G-check-bim.v4.2.5.zip). Strand designations were corrected to the forward strand, and reference/alternate (REF/ALT) allele designations were corrected using PLINK2 and the design files for OmniExpress and MetaboChip. Variants from chromosome X were excluded from imputation because the MetaboChip had very few SNPs from this chromosome. The imputation quality was quantified using the Rsq score (which estimates the squared correlation between imputed and true genotypes). Imputation was separately performed for genotypes from OmniExpress (1298 samples) and MetaboChip (1434 samples).

### Validating imputed genotypes

To evaluate the quality of imputation we evaluated whether genotype distribution at imputed variants in our study are consistent with distribution at the same variants genotyped in other studies. We examined consistency in genotype distribution at imputed variants versus genotype distribution at the same variants in our in- house Kuwaiti Arab Exome Variant database (John et al. 2018), and in genotype distribution at imputed variants versus genotype distribution at the same variants in GME Variome dataset (Scott et al. 2016). We used Fisher’s exact test to check the genotype distribution consistency for the above comparisons, and then we adjusted the derived *p* values using the Benjamini–Hochberg procedure. Adjusted *p* values < 0.05 were considered as genotype proportion inconsistency.

### Sensitivity analysis and transformations performed on the trait measurements

There was a concern that the glycemic, lipid, and blood pressure trait values that were determined in individuals who were receiving medication would not represent the naturally observed values in the population. We addressed this by applying adjustments to the measurements of the phenotypes by adding an average effect size to the phenotypes in the subjects under drug treatment. For FPG and HbA1c, we examined the in-house Knowledge-Based Health Records, an electronic health record system maintained at our institute, to calculate the average effect sizes among the native Kuwaiti T2D patients taking glucose-lowering medication who had improved their glucose levels (average reduction, − 2.59 mmol/L; *p* < 0.001). For lipid corrections, we used the recommendations of the Global Lipids Genetics Consortium and the Genetic Investigation of Anthropometric Traits Consortium (Willer et al. 2013). For antihypertensive medication, we used data reported in the literature (Tobin et al. 2005). The corrections implemented were as follows: (i) for participants taking lipid-lowering medication, we preadjusted TC to TC/0.8 and LDL to LDL/0.7, and non-HDL was determined by subtracting HDL from the preadjusted TC; (b) for participants taking antihypertensive drugs, we preadjusted SBP and DBP by adding 15 mmHg and 10 mmHg, respectively; and (c) for participants taking glucose-lowering medication, we preadjusted FPG and HbA1c values by adding 2.59 mmol/dl and 1%, respectively. Before generating the residuals, all the traits were adjusted (regular correction) for age, age^2^, and sex and for population stratification using the first four principal components (derived for each of the two study cohorts). Quantitative trait association tests require that the residual is normally distributed. Hence, we performed an inverse normal transformation on the raw residuals for the traits (except for TG, FPG, and HbA1c, for which log inverse transformation was performed, as it yielded a better Gaussian distribution). Association tests were performed with these inverse trait distributions.

### Association tests and meta-analysis

Genotype associations for the 13 quantitative metabolic traits were separately tested for each array platform using all genotyped and imputed SNPs that passed the QC threshold metrics (Rsq > 0.05 and MAF ≥ 5%). RVTESTS software (Zhan et al. 2016) was used to perform association tests using linear regression analysis for an additive genetic model. The imputed markers that were passed to the subsequent meta-analysis stage were required to meet the following QC criteria: (i) consistency in the allele and genotype frequencies of the markers between the cohorts genotyped with the two BeadChips; (ii) HWE < 10^−6^; (iii) p value for association < 0.05 in each of the two datasets; and (iv) consistency in the direction of association between the two datasets. Meta-analysis was performed using METAL software (Willer et al. 2010).

### *p* value thresholds for associations

The p value threshold was set at 5.0 × 10^−8^ for genome-wide significance, < 1.0 × 10^−6^ and > 5.0 × 10^−8^ for borderline to genome-wide significance, and > 1.0 × 10^−6^ and < 0.05 for suggestive evidence of association. Associations at suggestive p values were only considered if they were listed in the GWAS Catalog.

### Examining the NHGRI-EBI GWAS Catalog for reported association signals and variants

Reported traits for metabolic processes in the GWAS Catalog are highly diverse. The search terms relating to the 13 metabolic traits tested in our study belonged to five broad classes of curated trait search terms relating to anthropometry and obesity, blood pressure and hypertension, glycemia and diabetes, lipid profiles, and cardiometabolic phenotypes. We extracted 313 search terms (Supplementary Table S2) relating to the 13 study-specific metabolic traits from the NHGRI-EBI GWAS Catalog v1.0 (Buniello et al. 2019) (as accessed in February 2019): 99 search terms for the trait class of anthropometry and obesity, 46 for blood pressure and hypertension, 75 for glycemia and diabetes, 55 for lipid profiles, and 38 for cardiometabolic phenotypes. The GWAS Catalog contained 7668 SNP association signals for these terms. We compared the results of our meta-analysis to the known association signals from the GWAS Catalog and further removed any association that showed an inconsistent direction of effect from our subsequent analysis.

### Classifying variants associated with the tested 13 metabolic traits

Every variant that was associated with one or the other of the 13 metabolic traits (from the classes of anthropometry, glycemia, lipid, and blood pressure) in our meta-analysis was classified as either established or novel, depending on whether they appeared in the list of the 7668 known SNP association signals.

### Classifying the meta-analysis associations involving established variants

We classified meta-analysis associations involving established variants as direct, indirect, or broad, by comparing the associated metabolic traits in our study with known SNP association signals from the GWAS Catalog (as accessed in February 2019) as follows: (i) direct: the same trait was associated with the variant in the study cohort and the GWAS Catalog (e.g., a variant associated with BMI in our study cohort that was associated with BMI in the GWAS Catalog); (ii) indirect: the associated trait in the GWAS Catalog was a member of the class of traits related to the associated trait in our meta-analysis (see Supplementary Table S2 for classes of search terms relating to the groups of the 13 tested traits) (e.g., a variant associated with BMI in the study cohort that was associated with any trait described by the 99 search terms listed under the anthropometry and obesity class); or (iii) broad: the trait associated with the variant in our meta-analysis was related to any of the 313 metabolic traits (listed under the classes of anthropometry and obesity, blood pressure and hypertension, glycemia and diabetes, lipids, or cardiometabolic phenotypes) in the GWAS Catalog (e.g., a variant associated with BMI in the study cohort that was associated with adiponectin levels in the GWAS Catalog).

### Prioritizing variants based on a fine-mapping approach

In this study, we explored the identified top-associating signals (*p* value ≤ 5 × 10^−08^) using FINEMAP software to identify the plausible causal variants and regional heritability (Benner et al. 2016). FINEMAP efficiently explores sets of the most plausible causal variant configurations from a given genomic region using a shotgun stochastic search algorithm based on summary statistics from the meta-analysis and pairwise LD correlation statistics between the variants. We included variants within a ± 500 Kb region around the lead SNP with inverse-variance weighted summary statistics, computed the LD correlation matrix for both datasets independently using LDstore software (Benner et al. 2017), and then averaged them according to the following formula: meta_LD_matrix = (*n*1 × *R*1 + *n*2 × *R*2)/(*n*1 + *n*2), where *R*1 and *n*1 are the LD matrix and sample size of imputed data from OmniExpress, respectively, and *R*2 and *n*2 are the LD matrix and sample size of imputed data from MetaboChip, respectively. We created 95% credible causal variant sets by ranking the variants from the region based on their decreasing posterior probability of association and estimated regional heritability to understand the proportion of phenotypic variance of the complex trait attributable to a credible set of SNPs.

### Estimation of power for replicating and not replicating variants at genome-wide significance

We performed power calculation for replicating and not-replicating variants to estimate power required to expect them at *p* value ≤ 5E−08 using R scripts (available at https://github.com/kaustubhad/gwas-power) that were developed using formulas as per Visscher et al. (2008). The power_beta_maf function from the script was used to calculate power based on a range of values of beta and MAFs (while values for sample size (*n*) and pval were fixed).

### Functional variant prioritization

We used the Ensembl Variant Effect Predictor tool (McLaren et al. 2016) to annotate the functional consequences of variants. We assessed the proximity of the variants to the TSS using ChIPpeakAnno (Zhu et al. 2010) and TSS.human.GRCh38 data (available from the Bioconductor software suite at https://bioconductor.org/packages/). We used the tools of SuRFR (Ryan et al. 2014) (SNP Ranking by Function R package), which interacts with SAILR (SNP Annotation Information List R package) to prioritize variants based on the likelihood of their function using features such as promoter, CpG, CpG shore, DNase HSs, DNase footprints, TFBS, TSS, chromatin states (histone acetylation and methylation), conserved sequences, and enhancers. To aid in the variant prioritization, we used background variants from European populations and used a pre-trained weighting model for complex disease variants, with default parameter values (Ryan et al. 2014).

### Expression quantitative trait loci analysis

We examined genotype-tissue expression data using GTeX v8 (https://www.gtexportal.org) to assess the involvement of the discovered variants in the regulation of gene expression. Two levels of analysis were carried out: (i) all eQTL’s with *p* value ≤ 0.05 were considered; and (ii) only those eQTL’s with *p* value ≤ 0.05 AND *q* value ≤ 0.05 were considered. The *q* value is similar to the well known *p *value, except it is a measure of significance in terms of the false discovery rate rather than the false positive rate (Storey and Tibshirani 2003). Beta distribution-adjusted empirical p values from FastQTL were used to calculate q-values.

## Electronic supplementary material

Below is the link to the electronic supplementary material.

Supplementary file1 (DOCX 13940 kb)

Supplementary file2 (DOCX 47 kb)

Supplementary file3 (XLSX 236 kb)

Supplementary file4 (XLSX 320 kb)

Supplementary file5 (XLSX 95 kb)

Supplementary file6 (XLSX 552 kb)

## Data Availability

All the data on the variants and their associations with metabolic traits are provided as supplementary data sets. GWAS summary statistics is available to researchers upon writing to the corresponding authors.
