# Supplementary material for: Genome-wide landscape establishes novel association signals for metabolic traits in the Arab population
Source: Hum Genet. 2020 Sep 9;140(3):505–28. doi: 10.1007/s00439-020-02222-7 (PMC7889551; doi:10.1007/s00439-020-02222-7)

**Supplementary Figure S1. Scatterplots presenting the first three principal components (PC1 versus PC2; PC2 versus PC3; PC1 versus PC3) derived from a merged data set of representative populations from the 1000 Genomes Project and the samples genotyped on** **HumanOmniExpress BeadChip (A-C) and HumanCardio-Metabo BeadChip** **(D-F). Individuals from the study cohort are labelled as Arabs living in Kuwait (ALK) and a red oval is drawn around the cluster of Arab individuals in the scatter plots.**

| **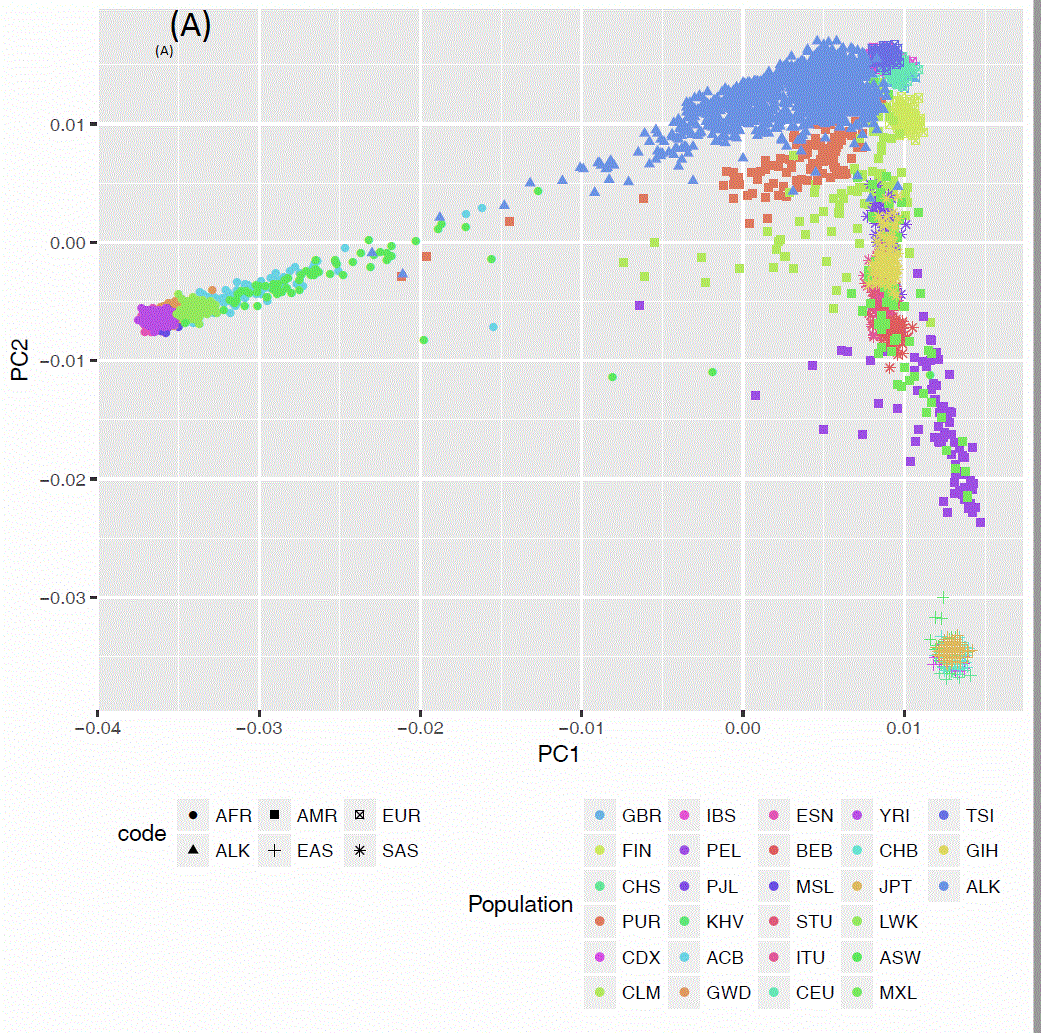** | **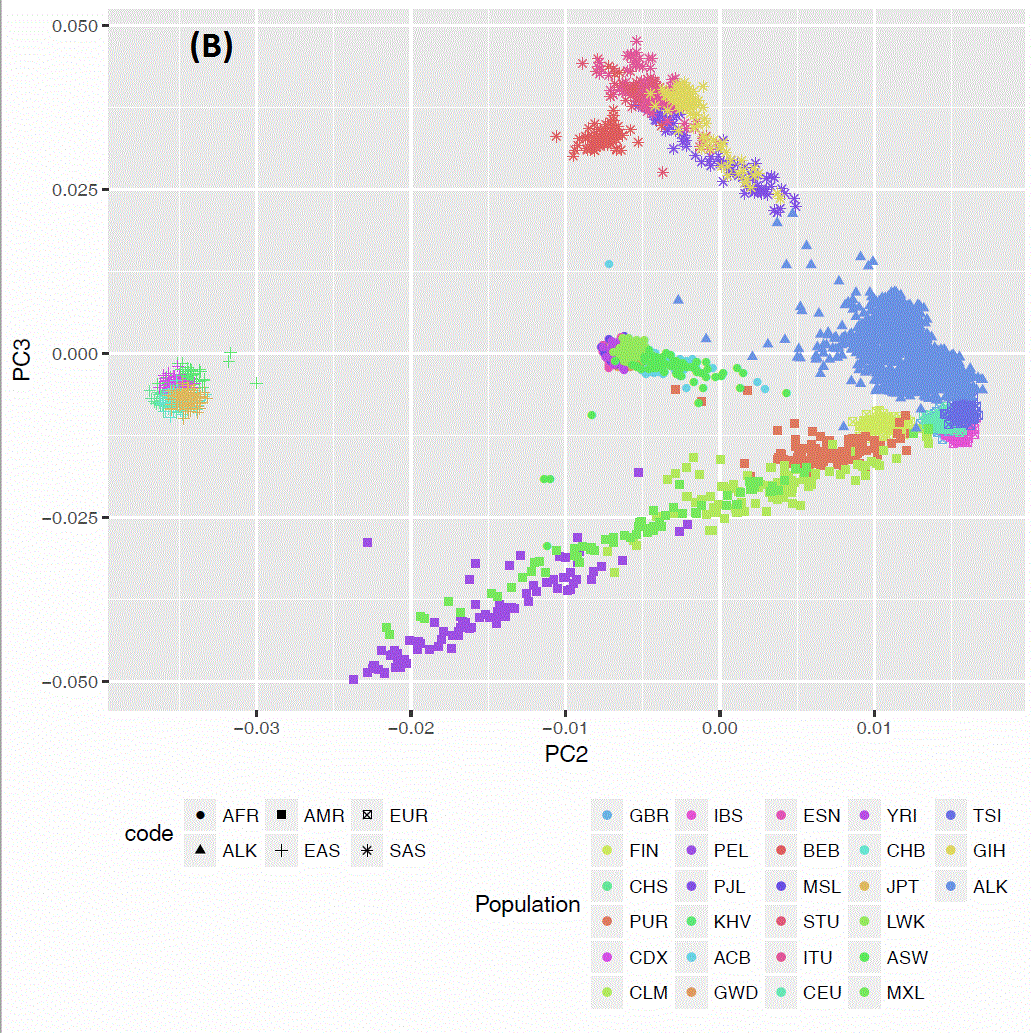** |
| --- | --- |
| **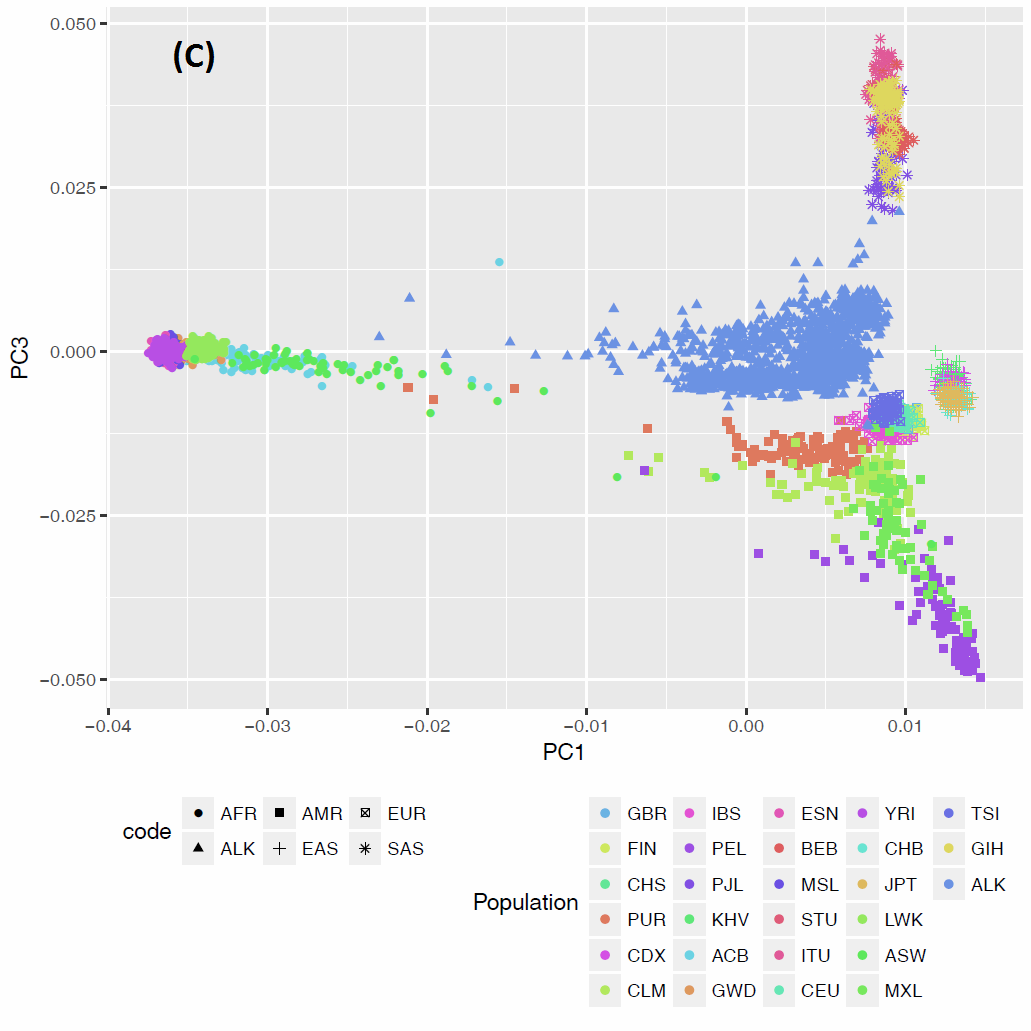** | **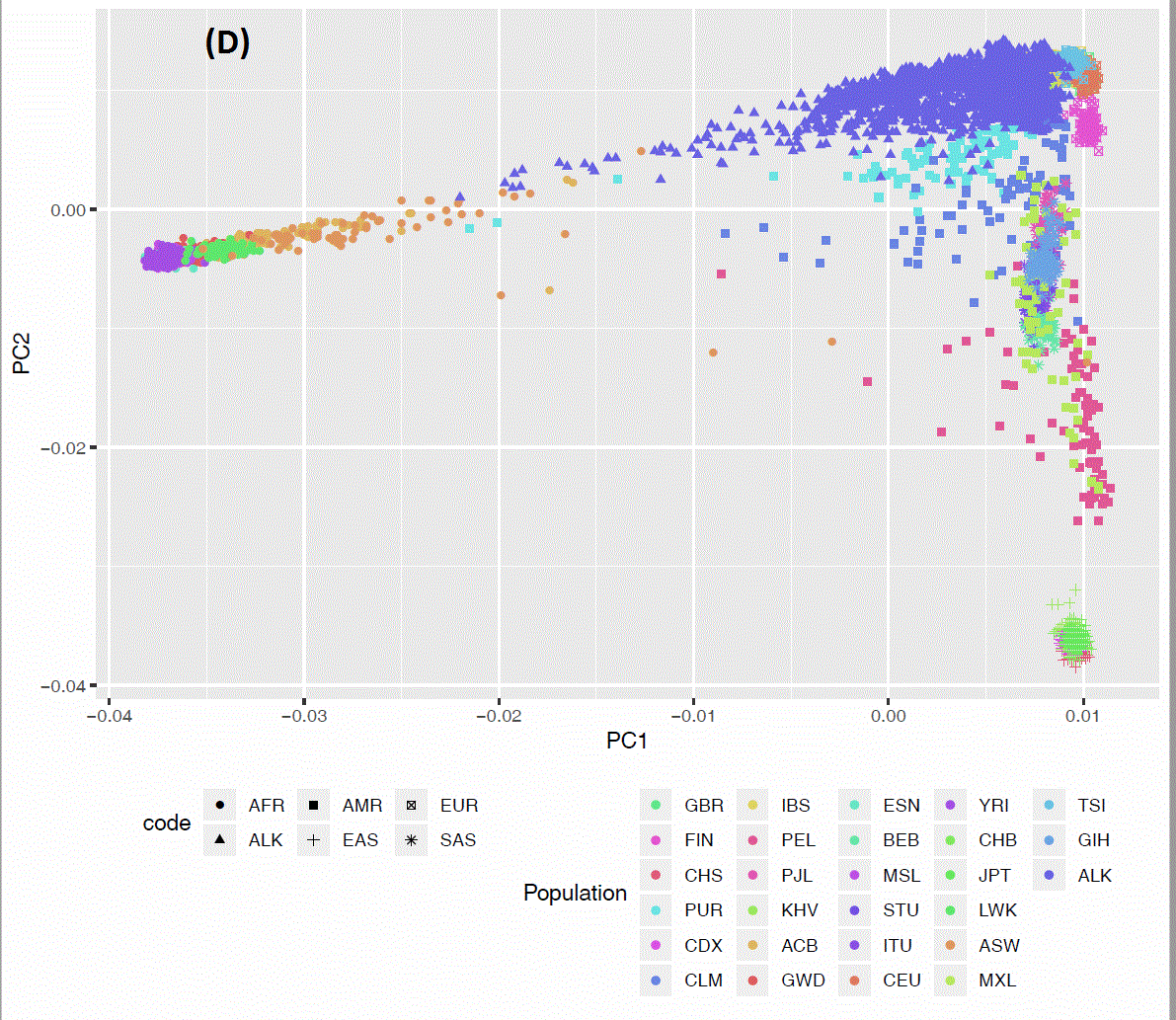** |
| **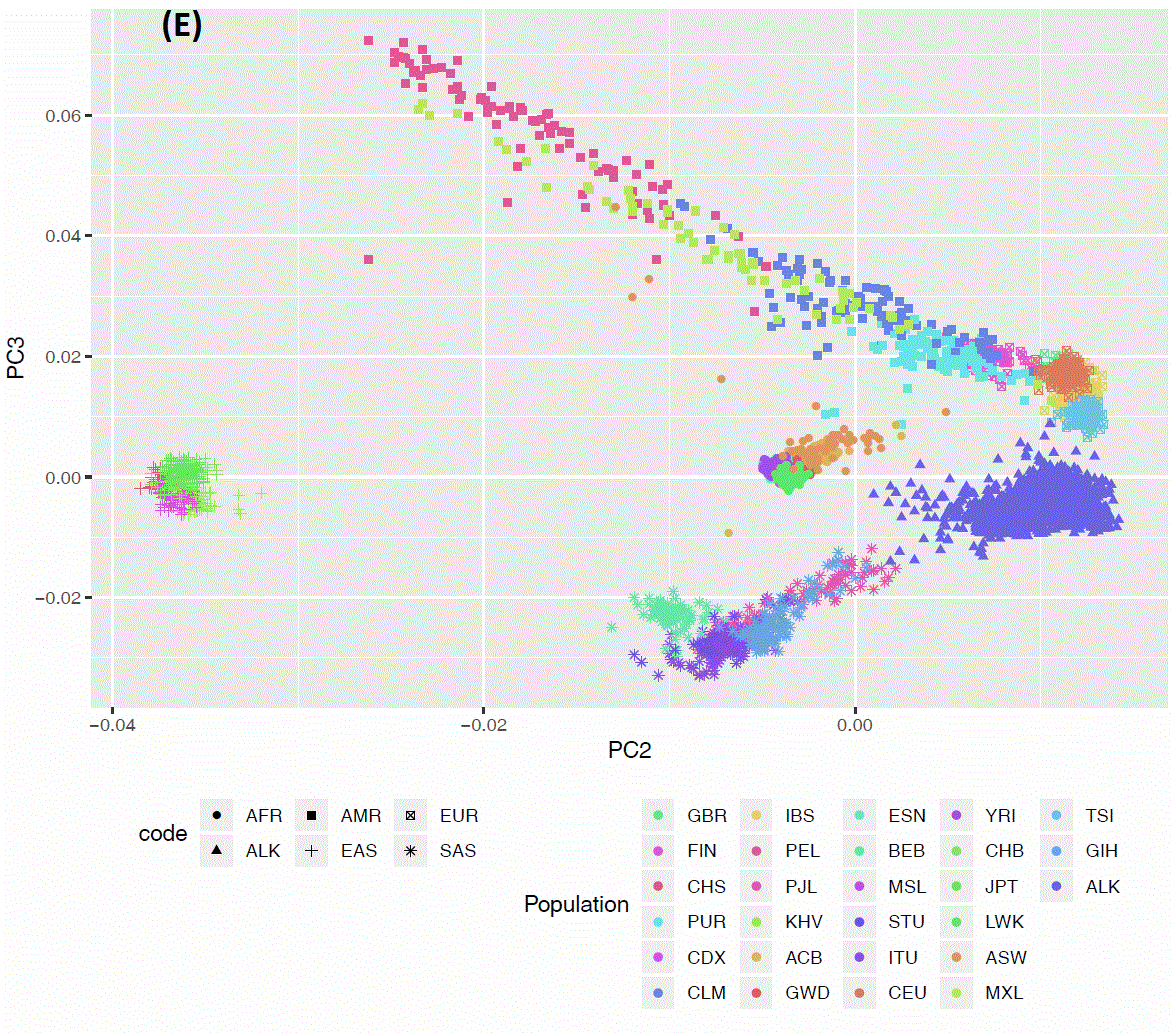** | **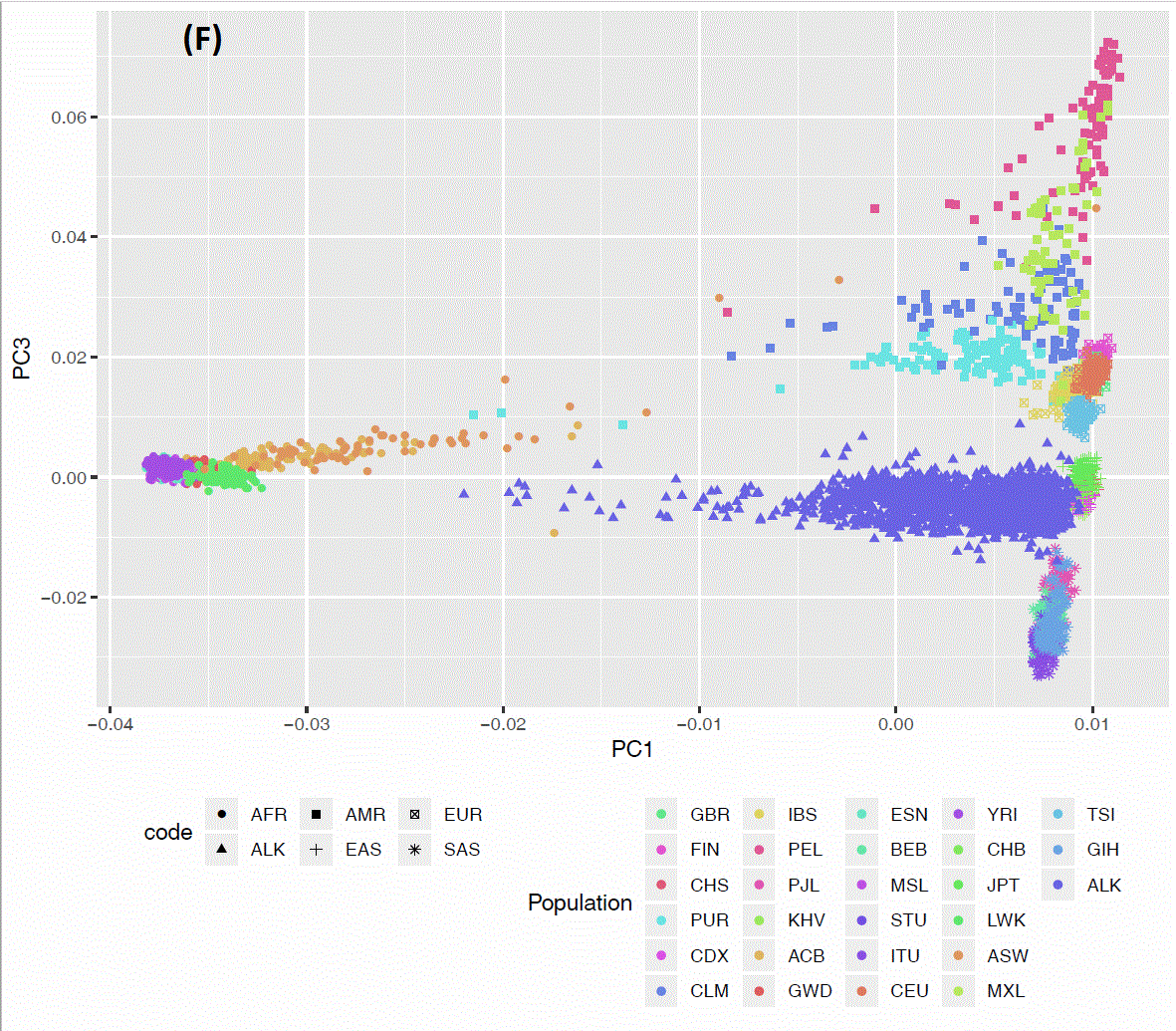** |

**Supplementary Figure S2. Plot of imputation mean quality score (Rsq) against minor allele frequency (MAF) of imputed markers among the Kuwaiti population.** Rsq represents per-sample imputation confidence score between true and imputed genotypes averaged over imputation chunks. Mean quality score increases as MAF increases; and it increases with increased array SNP coverage. The plot (**A**) depicts the mean score values against MAF of imputed markers considered in our meta-analysis; and the plot (**B**) indicates the proportion of imputed variants falling into different bins of MAF, in each of the two platforms, before (red and green lines) and after (blue and grey lines labelled as OE* and CM*). filtering the variants at Rsq>0.50. In the line drawings labelled OE* and CM* (in both the plots of (A) and (B)), only those markers with Rsq>0.50 and MAF>0.05 were considered and only such markers were considered in association tests and meta-analysis.


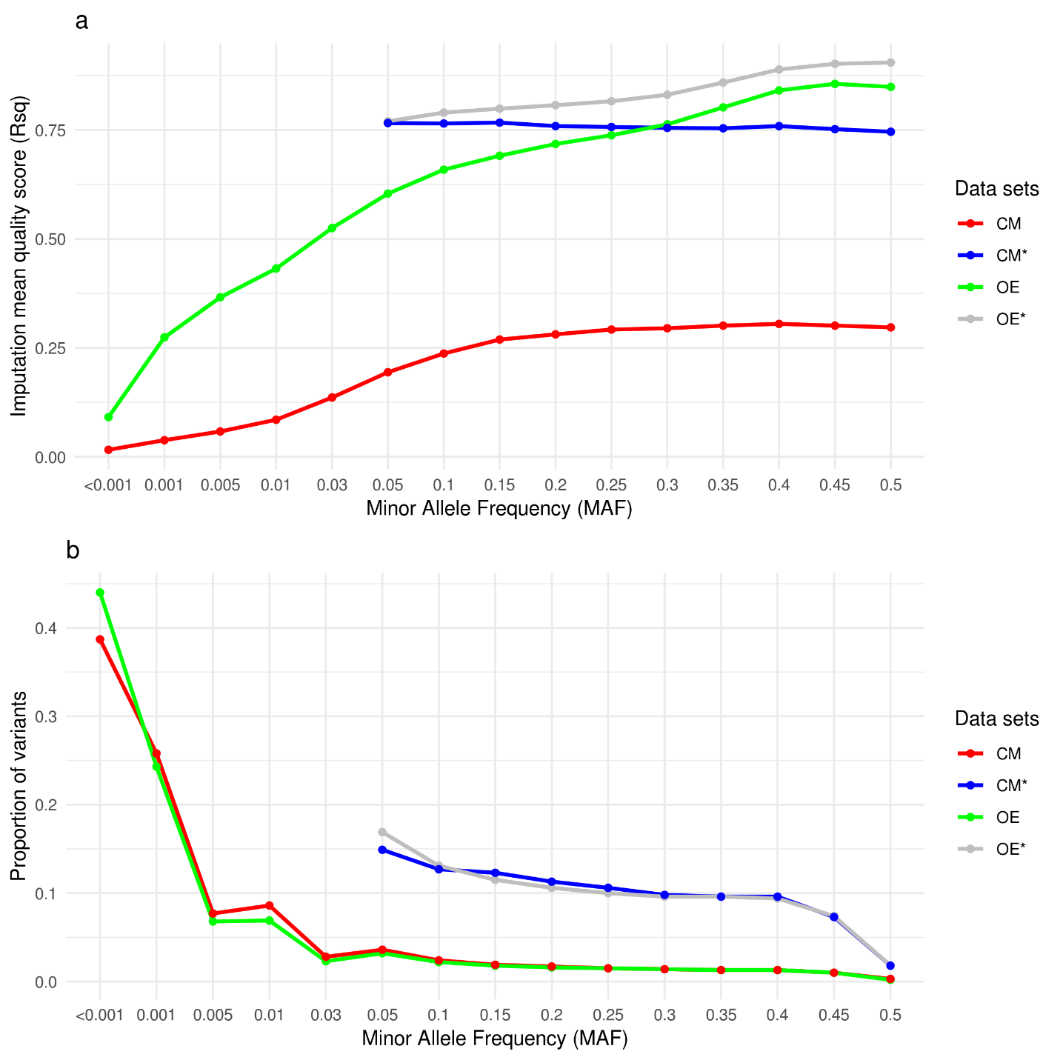


**Supplementary Figure S3. Quantile–quantile plots depicting the expected and observed −log_10_(*p*-values) for association of the variants (genotyped or imputed) with the 13 metabolic traits for the two studies and the meta-analysis. Values for genomic inflation (**λ) for each of the 13 traits in each of the two studies and the combined meta-analysis are mentioned.

| 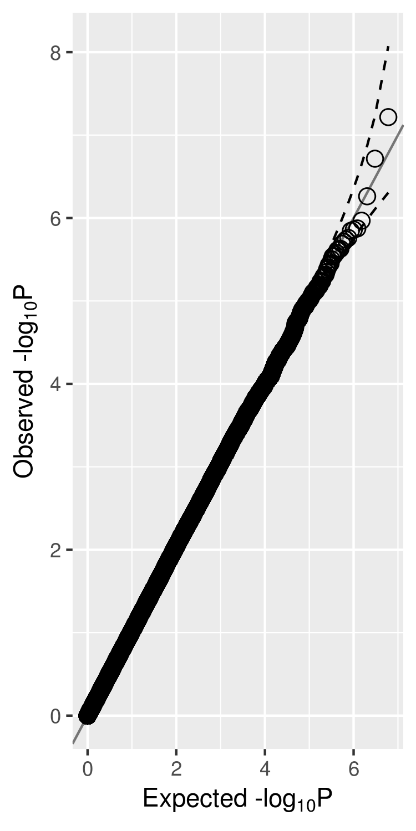 | 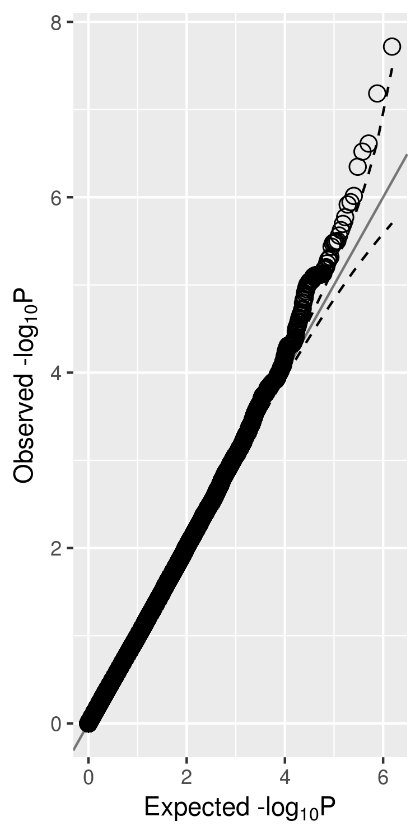 | 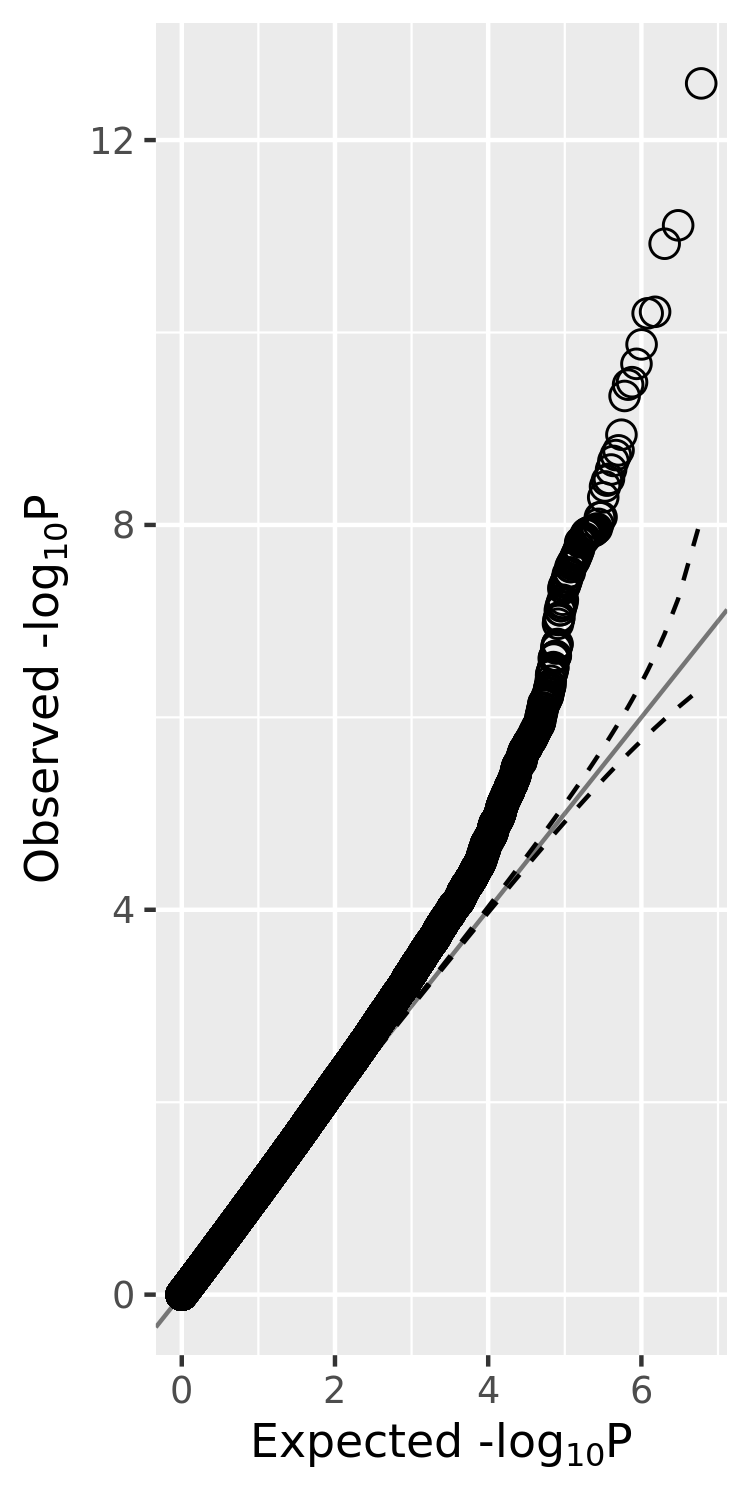 |
| --- | --- | --- |
| HDL_INV: OE, λ=1.034 | HDL_INV:CM, λ=1.013 | HDL_INV: Meta, λ=1.097 |
| 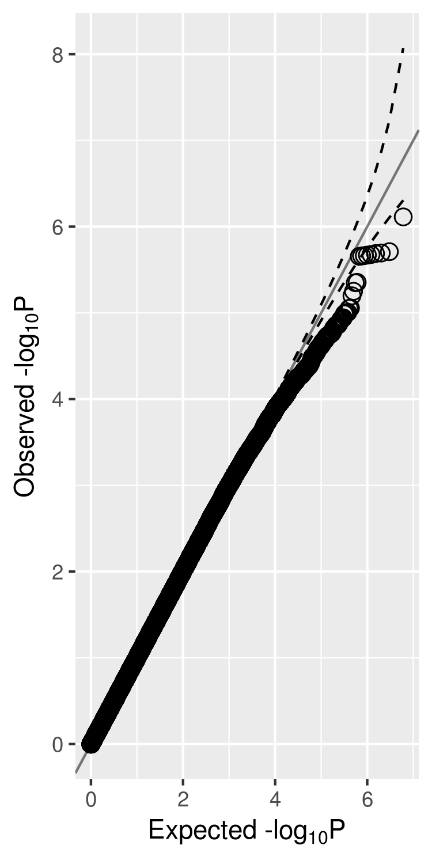 | 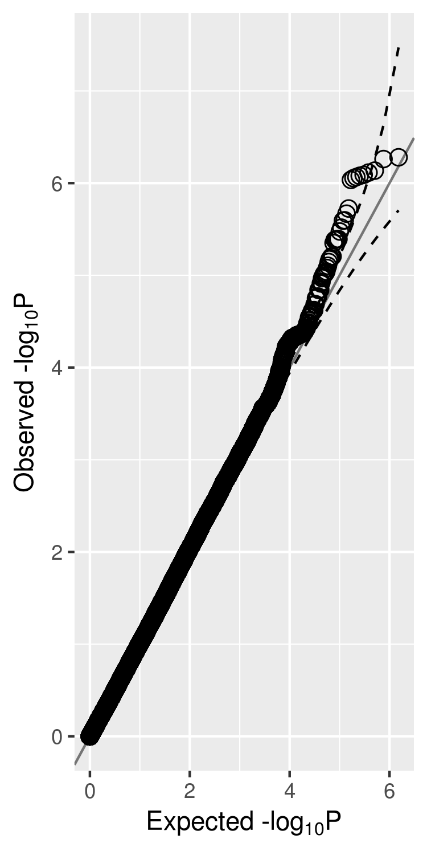 | 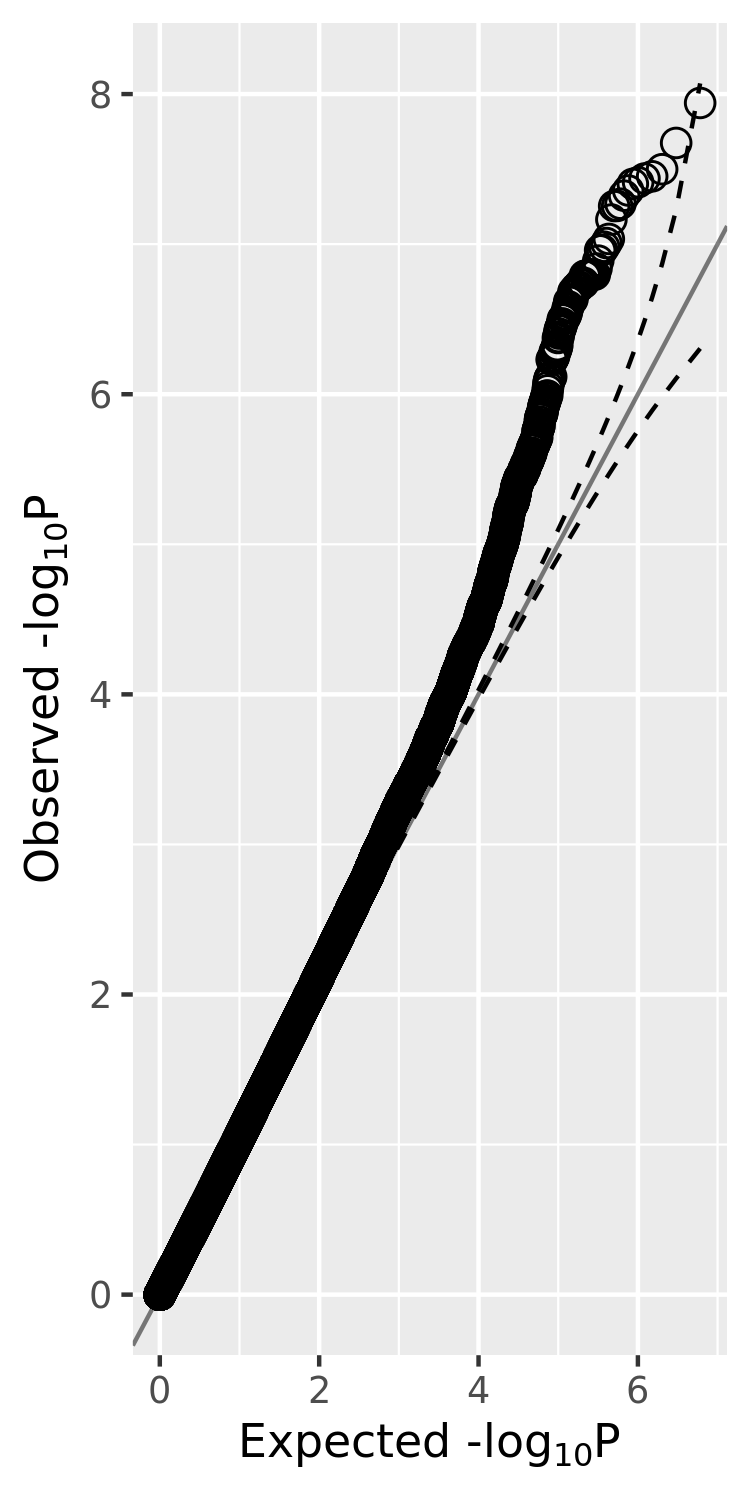 |
| LDL_INV: OE, λ=0.993 | LDL_INV:CM, λ=0.98 | LDL_INV: Meta, λ=1.055 |
| 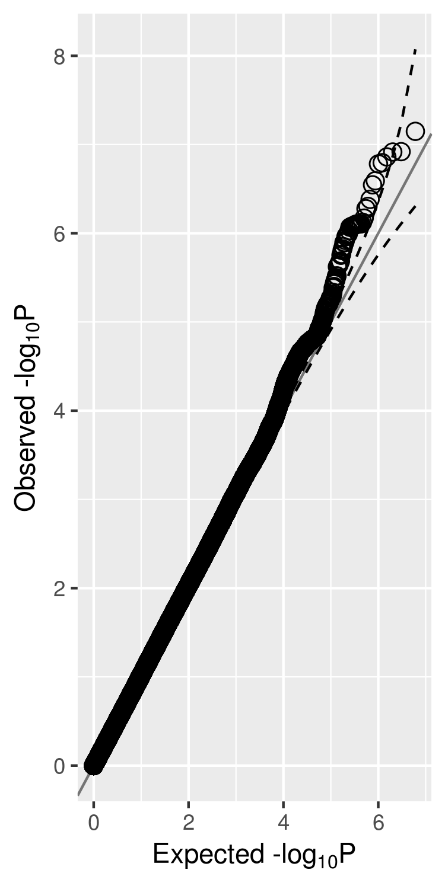 | 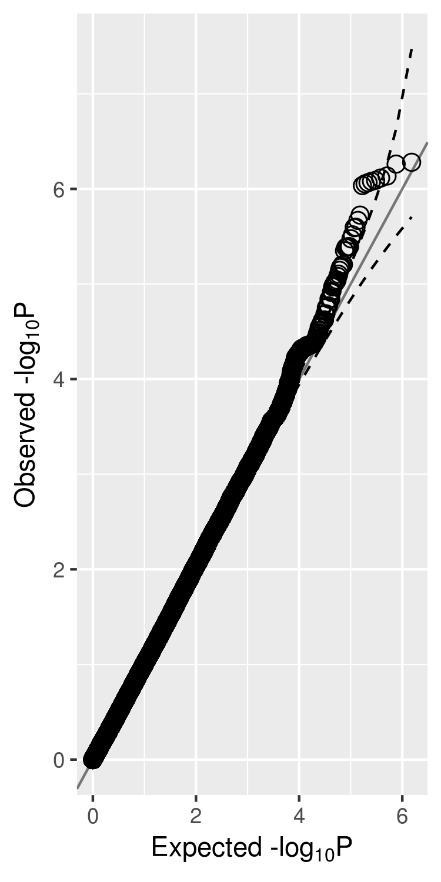 | 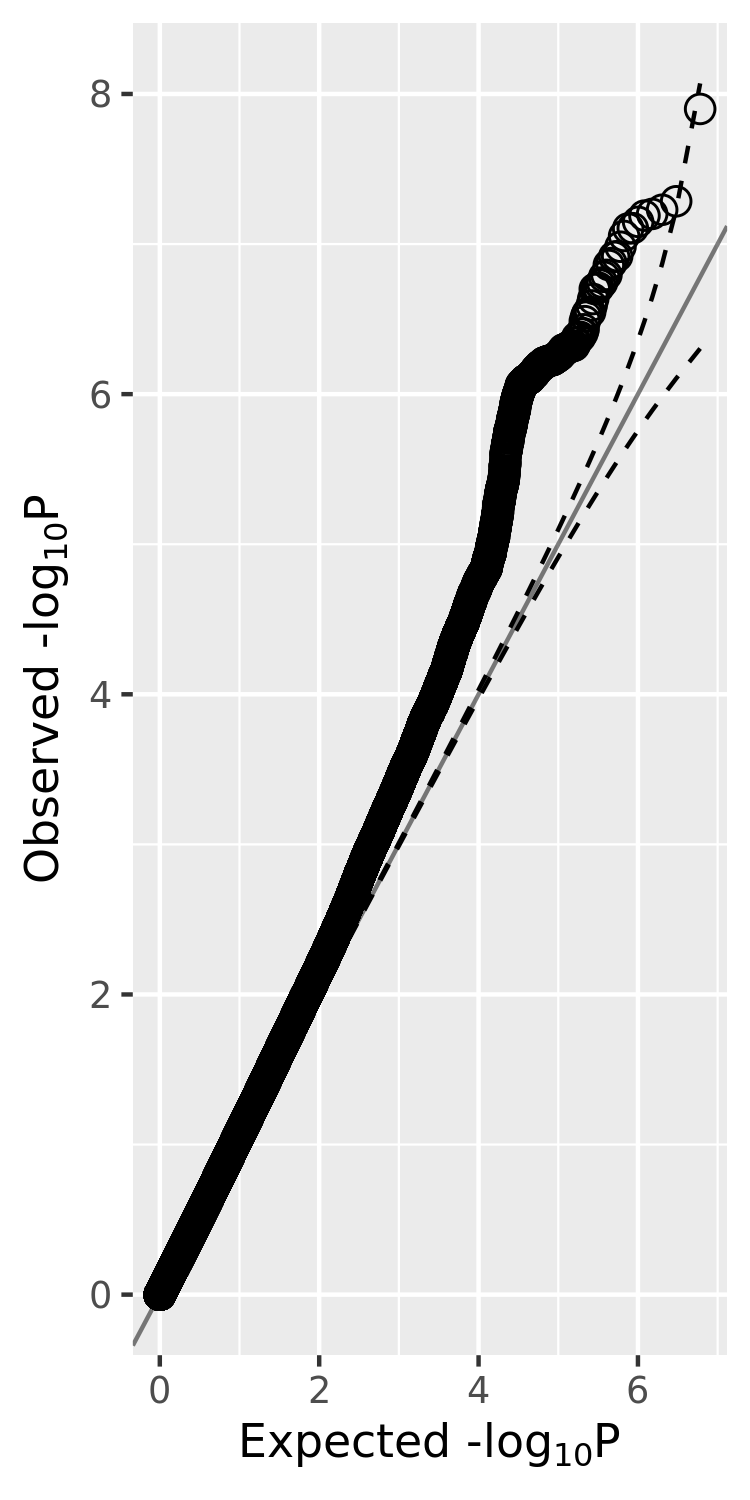 |
| TG_INV: OE, λ=1.02 | TG_INV:CM, λ=0.98 | TG_INV: Meta, λ=1.079 |
| 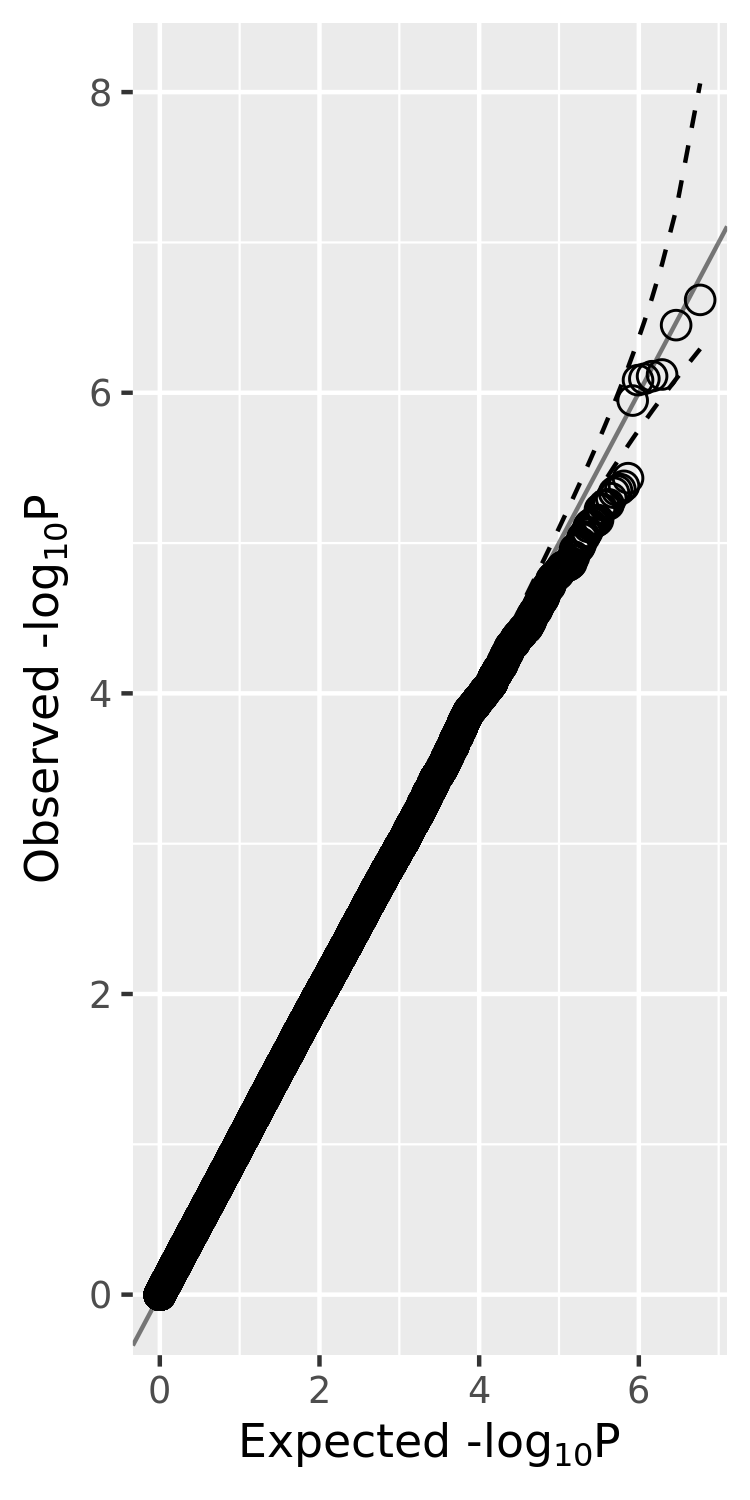 | 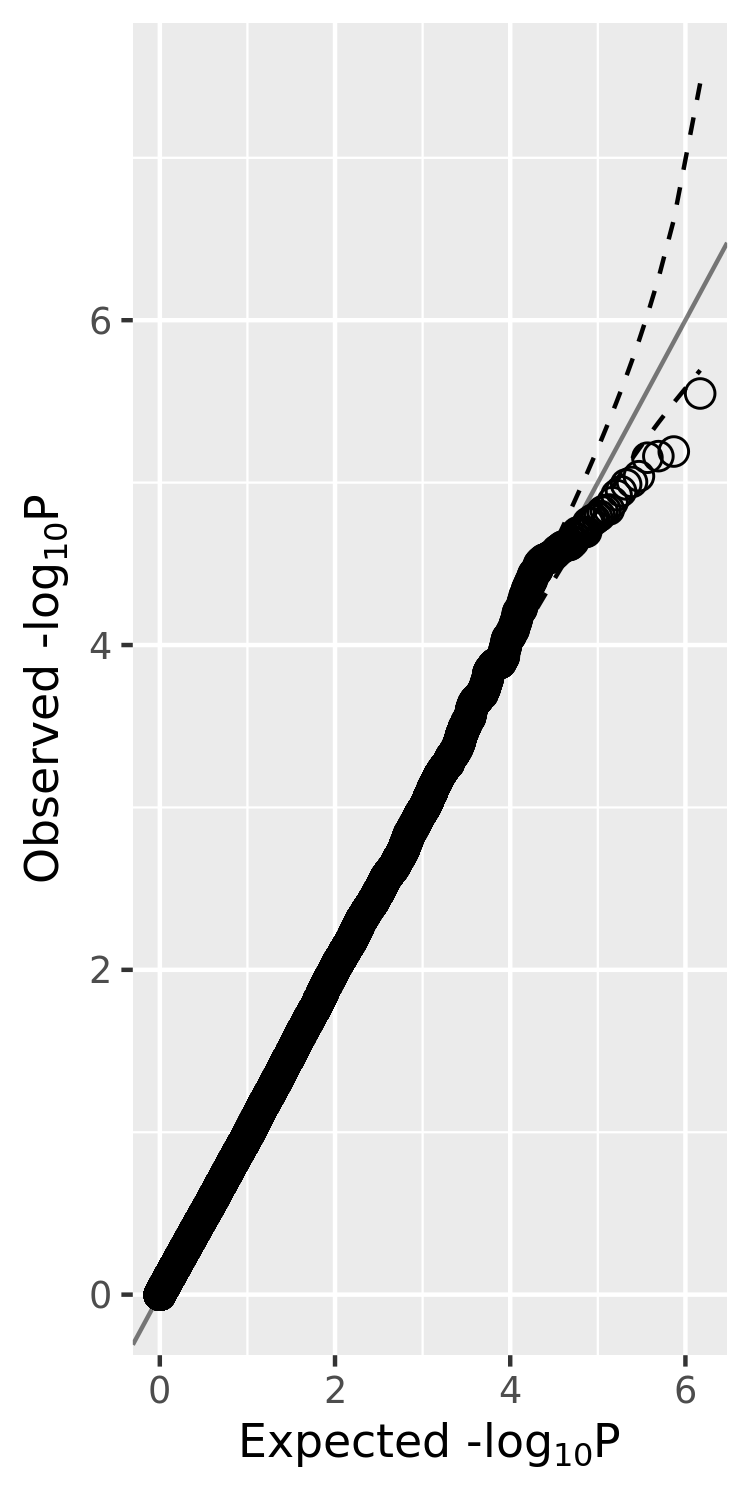 | 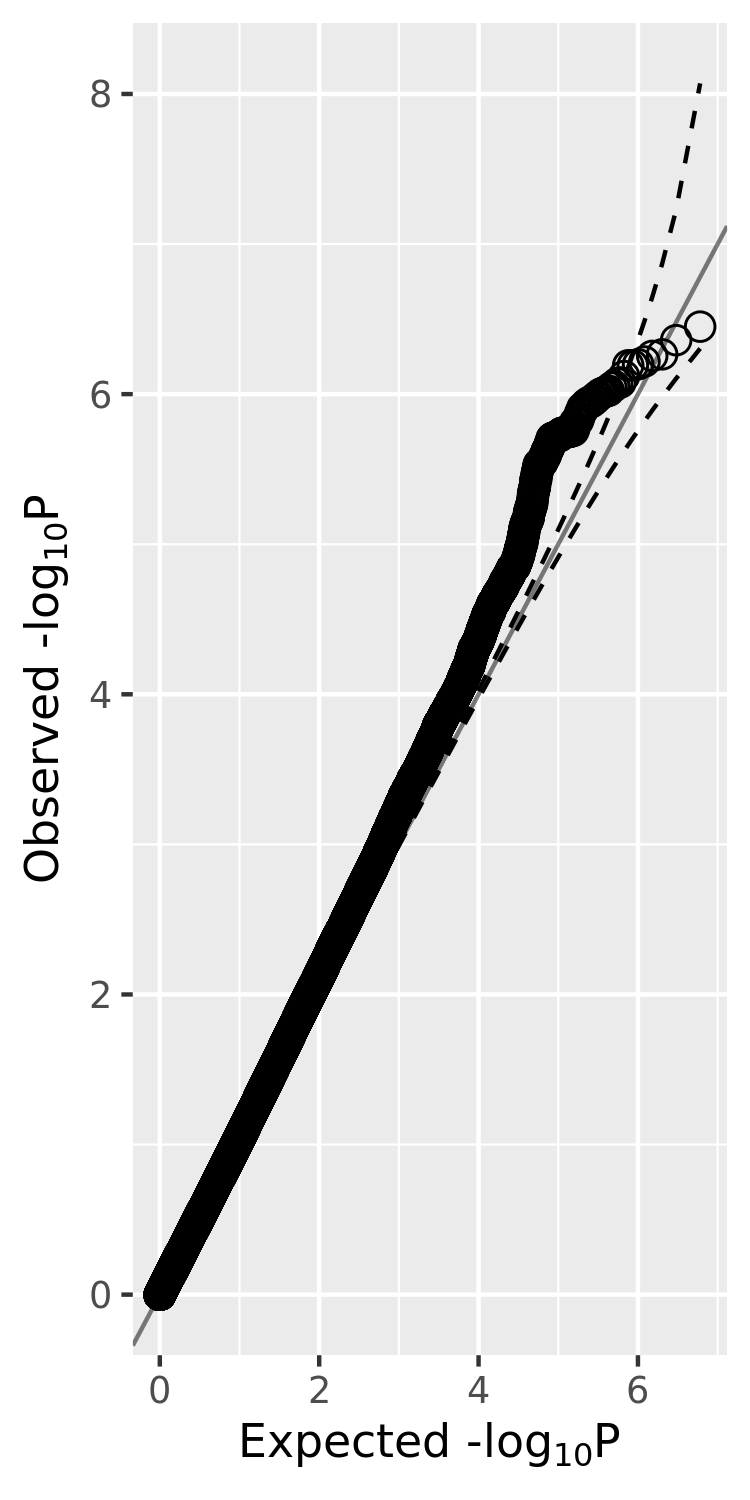 |
| TC_INV:OE, λ=1.01 | TC_INV:CM, λ=0.97 | TC_INV: Meta, λ=1.065 |
| 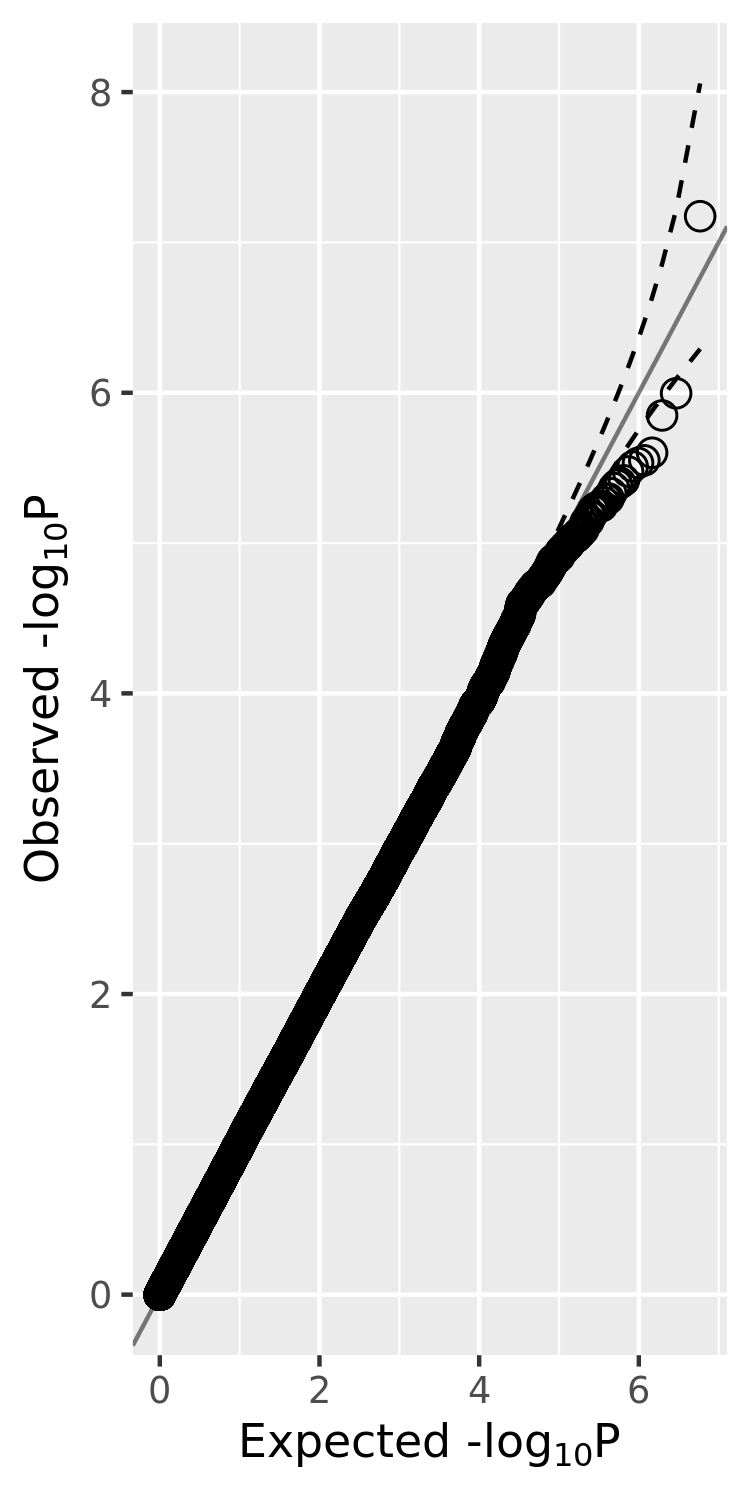 | 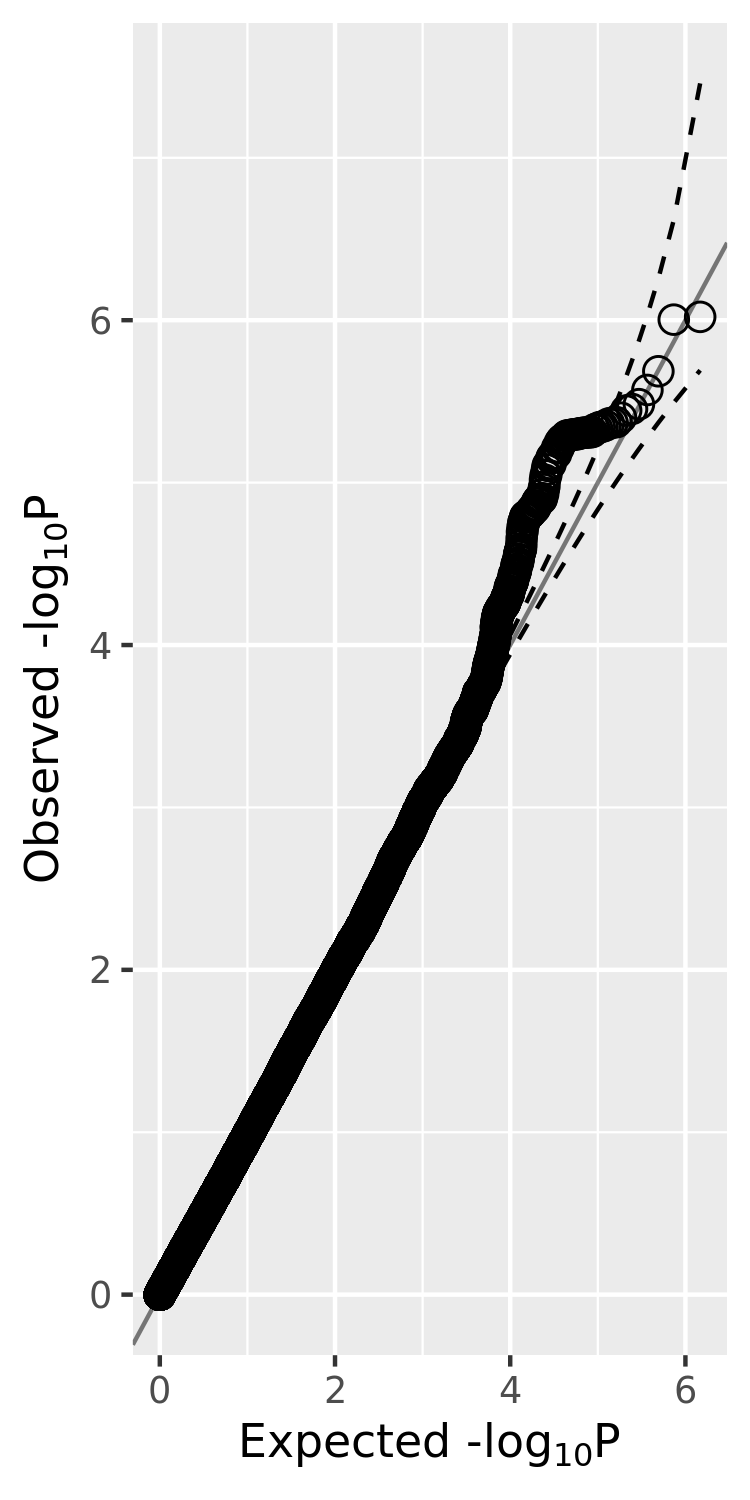 | 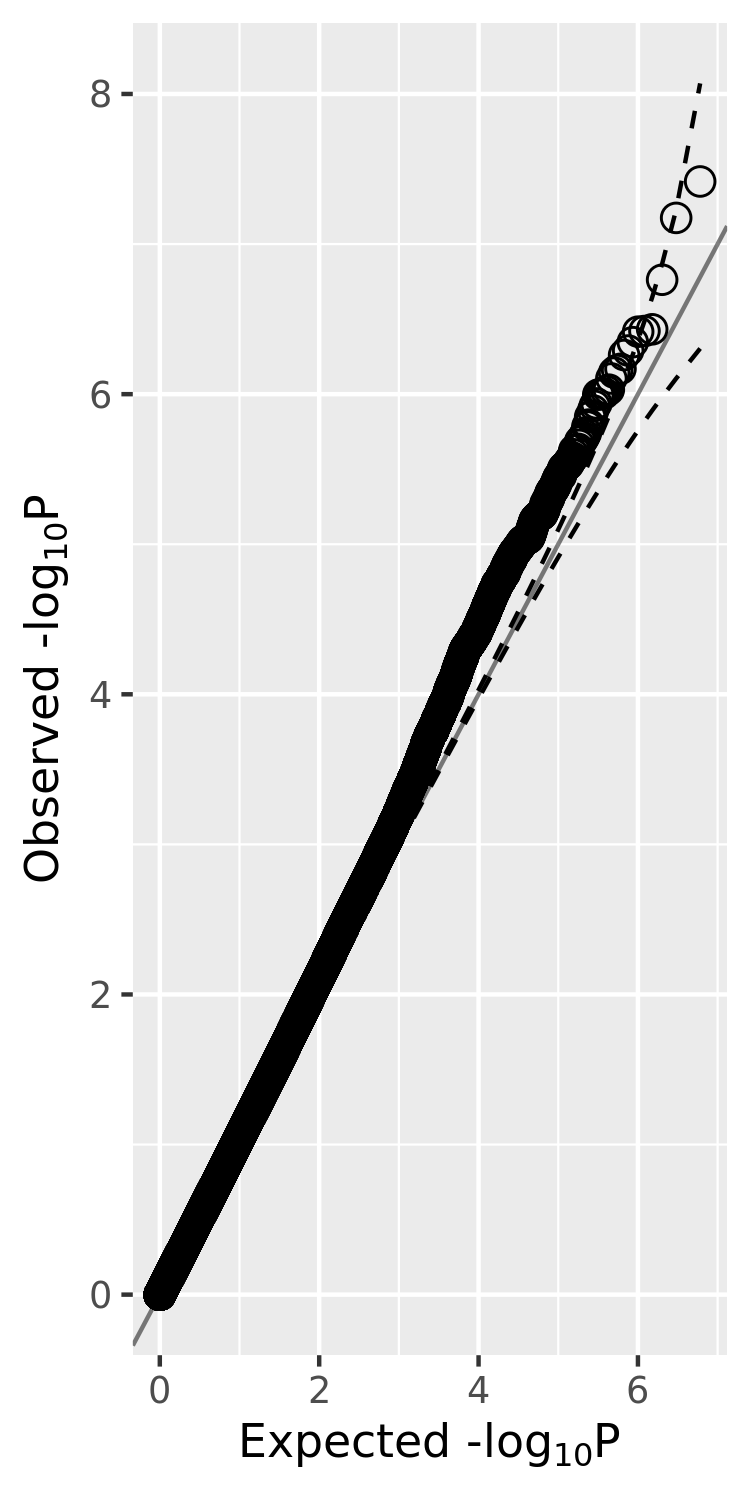 |
| Non-HDL_INV:OE, λ=1.003 | Non-HDL_INV:CM, λ=0.971 | Non-HDL_INV: Meta, λ=1.065 |
| 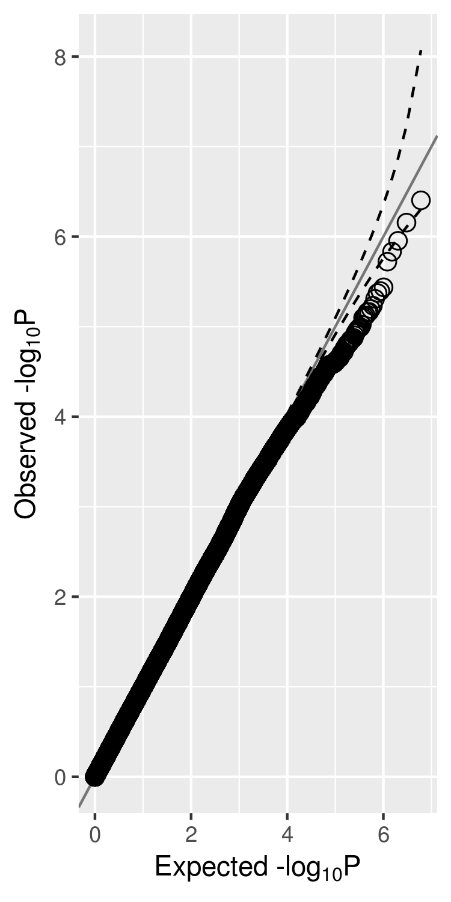 | 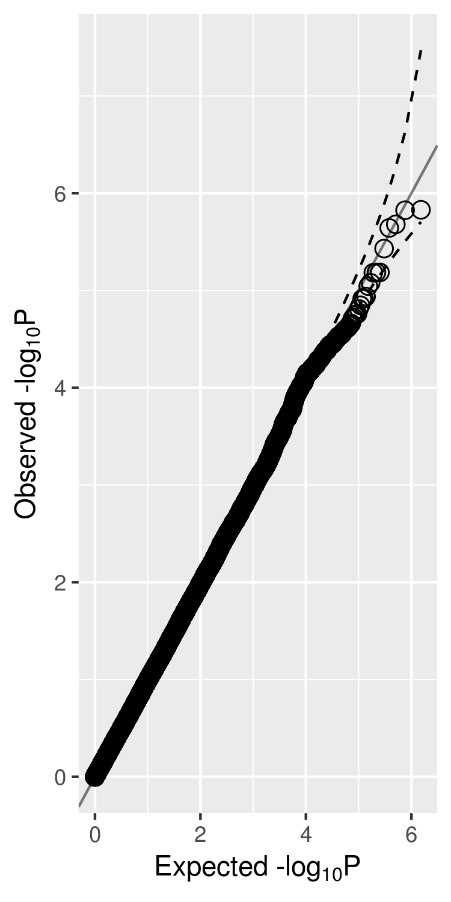 | 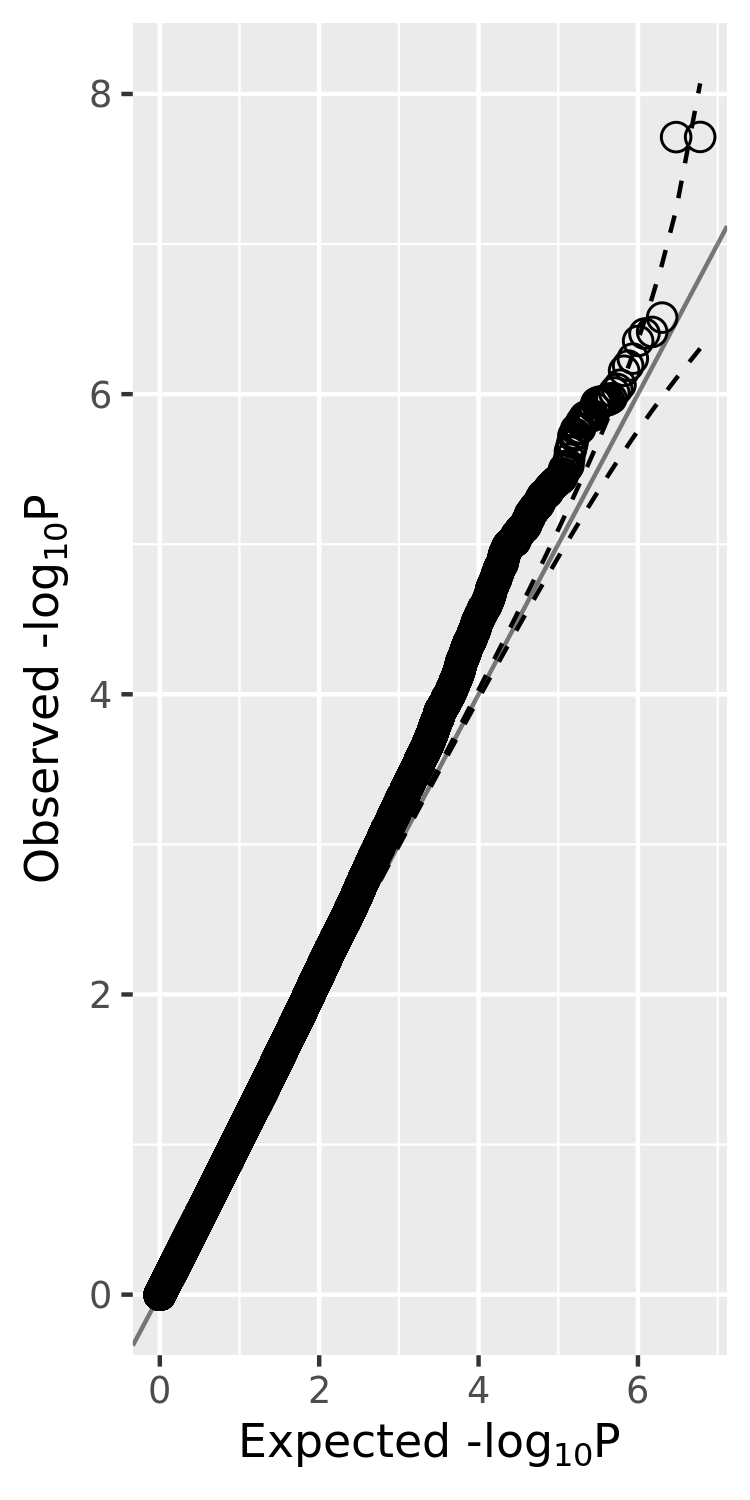 |
| DBP_INV: OE, λ=0.994 | DBP_INV:CM, λ=0.986 | DBP_INV: Meta, λ=1.07 |
| 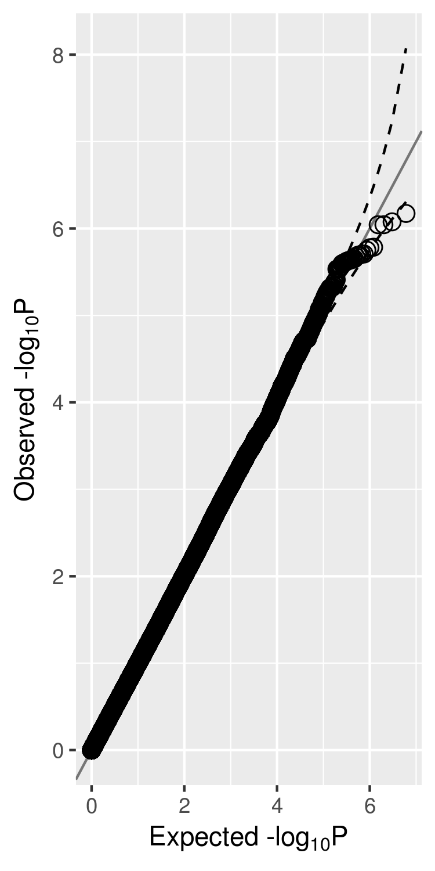 | 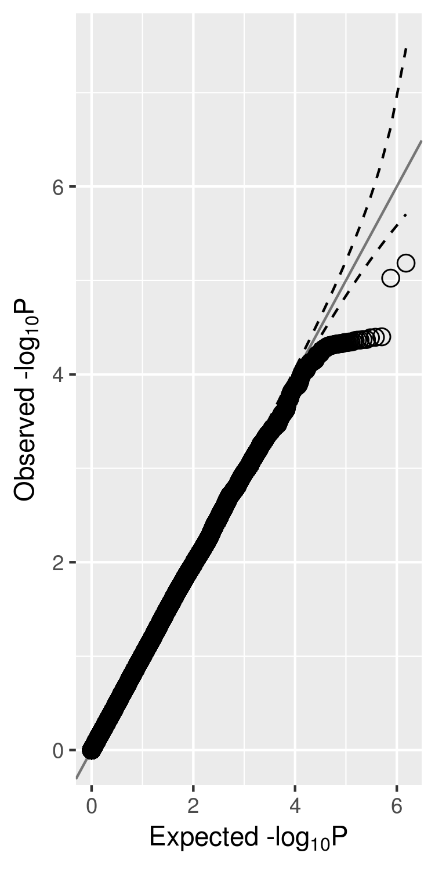 | 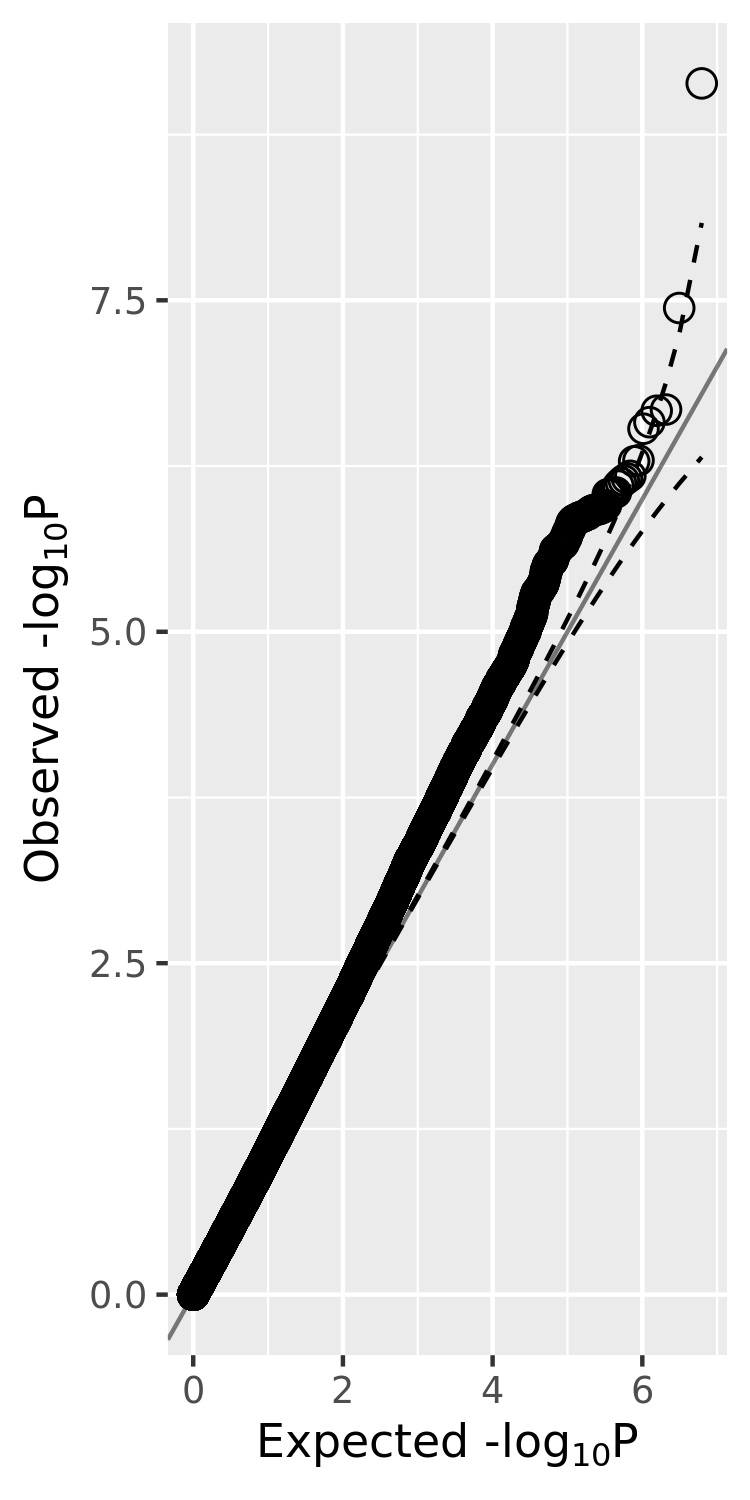 |
| FPG_INV:OE, λ=1.003 | FPG_INV:CM, λ=0.984 | FPG_INV: Meta, λ=1.069 |
| 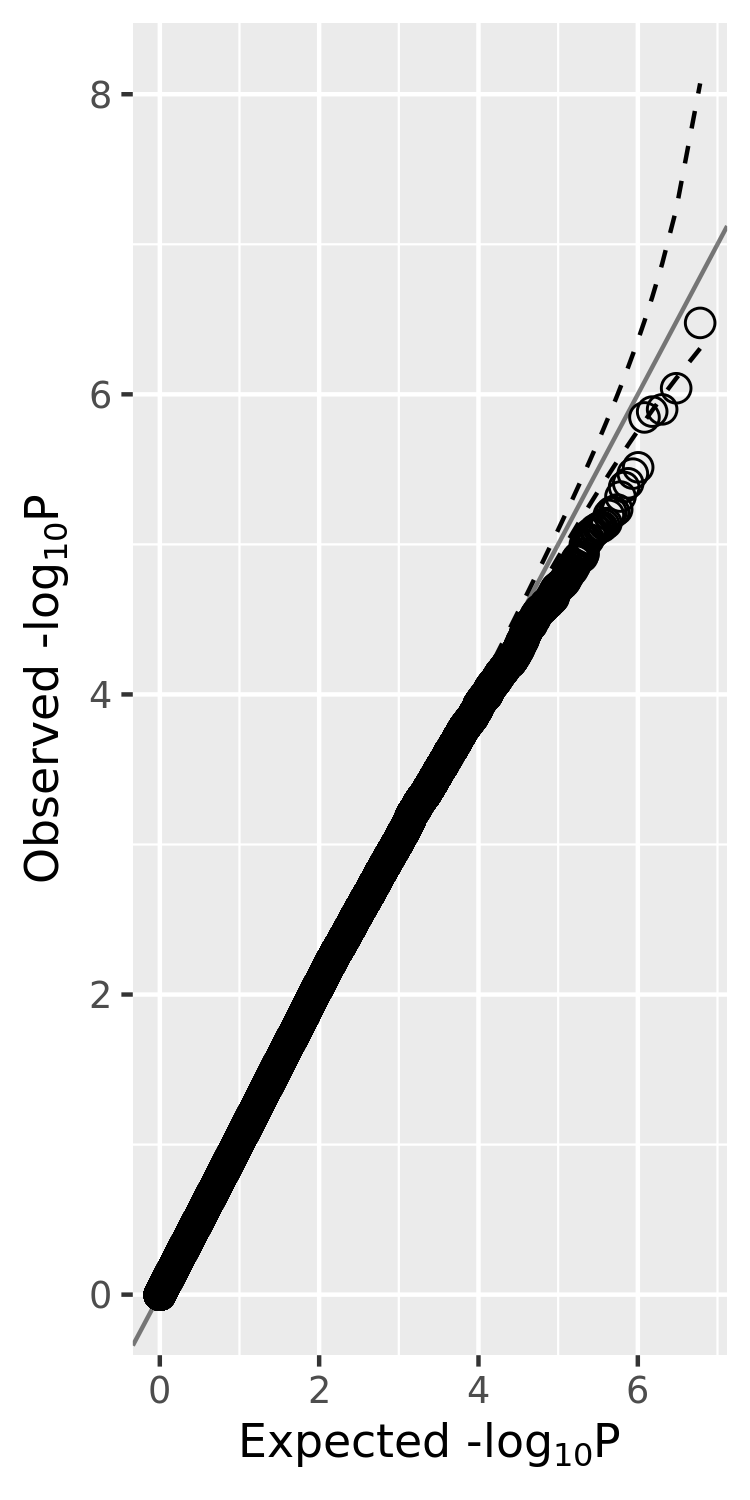 | 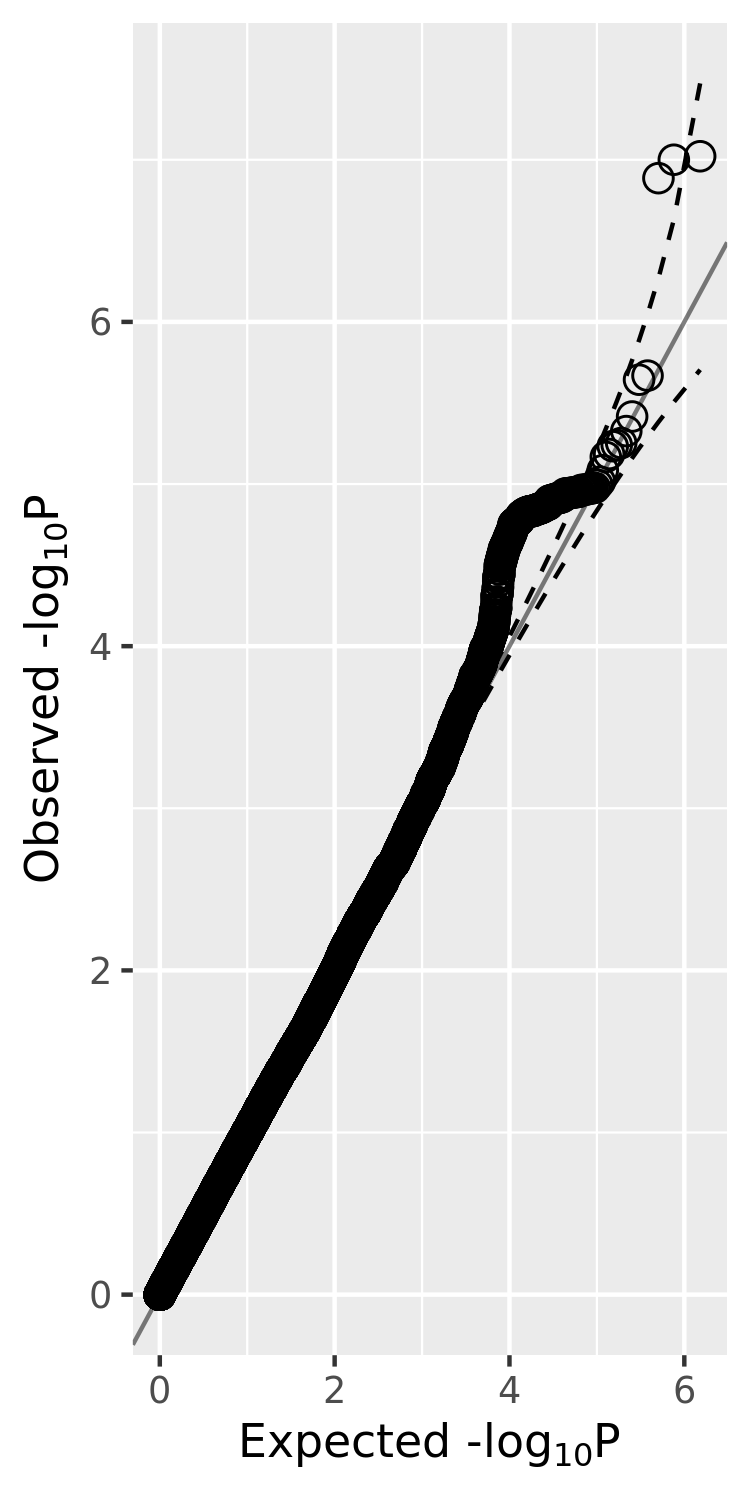 | 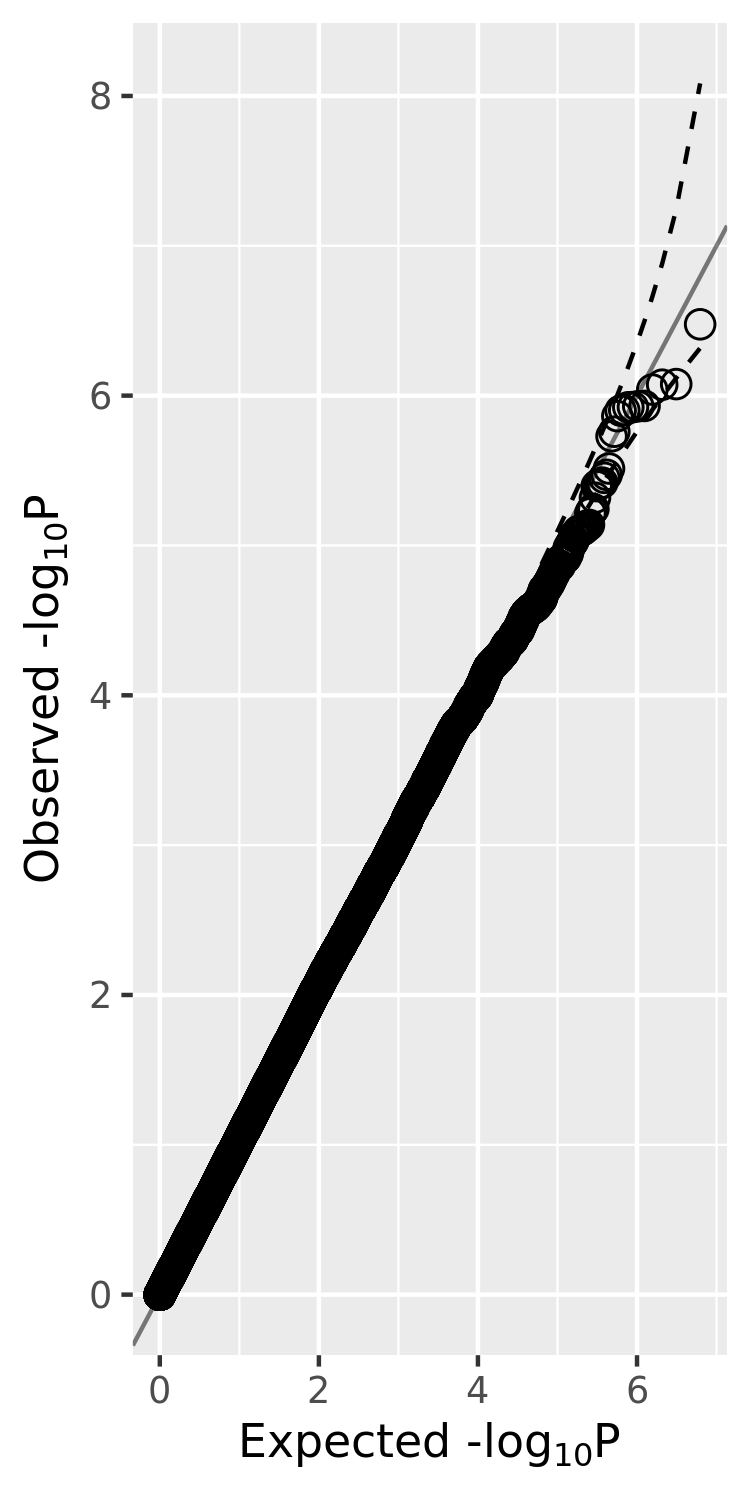 |
| HbA1C_INV:OE, λ=1.006 | HbA1C_INV:CM, λ=1.042 | HbA1C_INV: Meta, λ=1.053 |
| 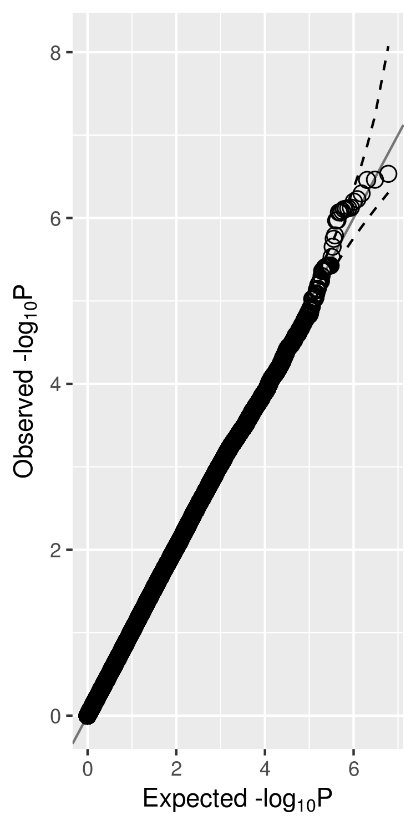 | 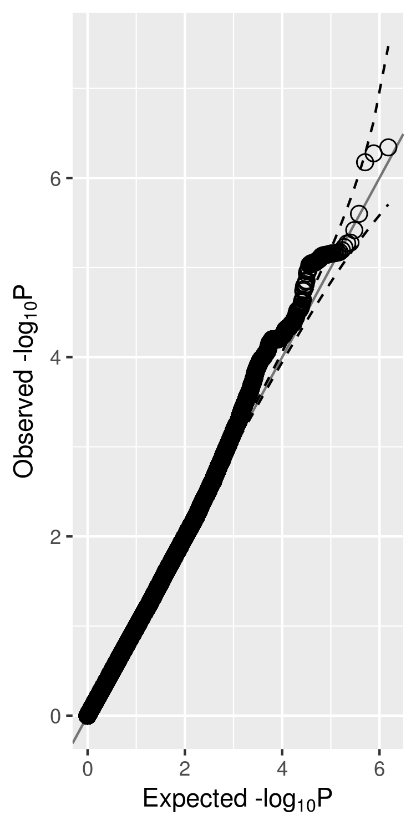 | 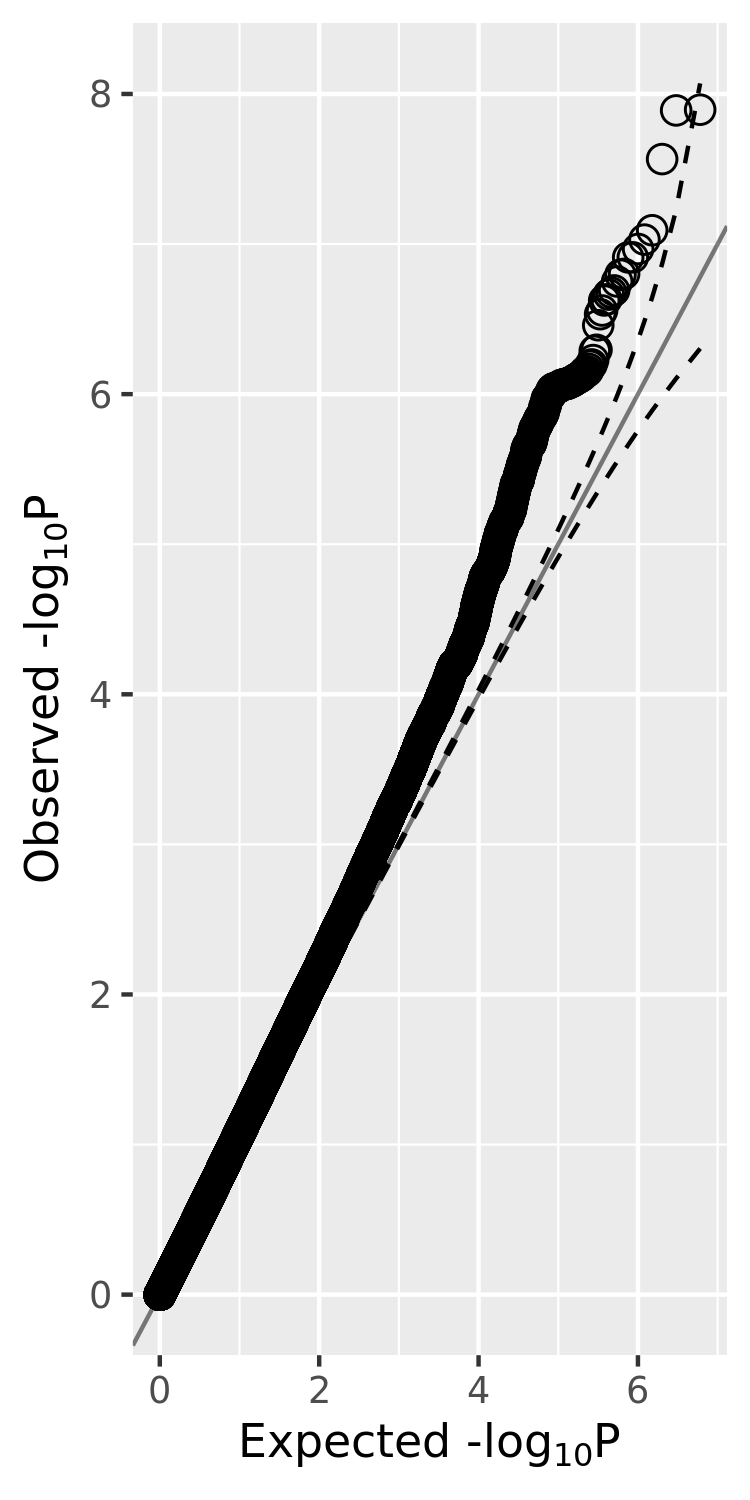 |
| SBP_INV:OE, λ=1.029 | SBP_INV:CM, λ=0.979 | SBP_INV: Meta, λ=1.09 |
| 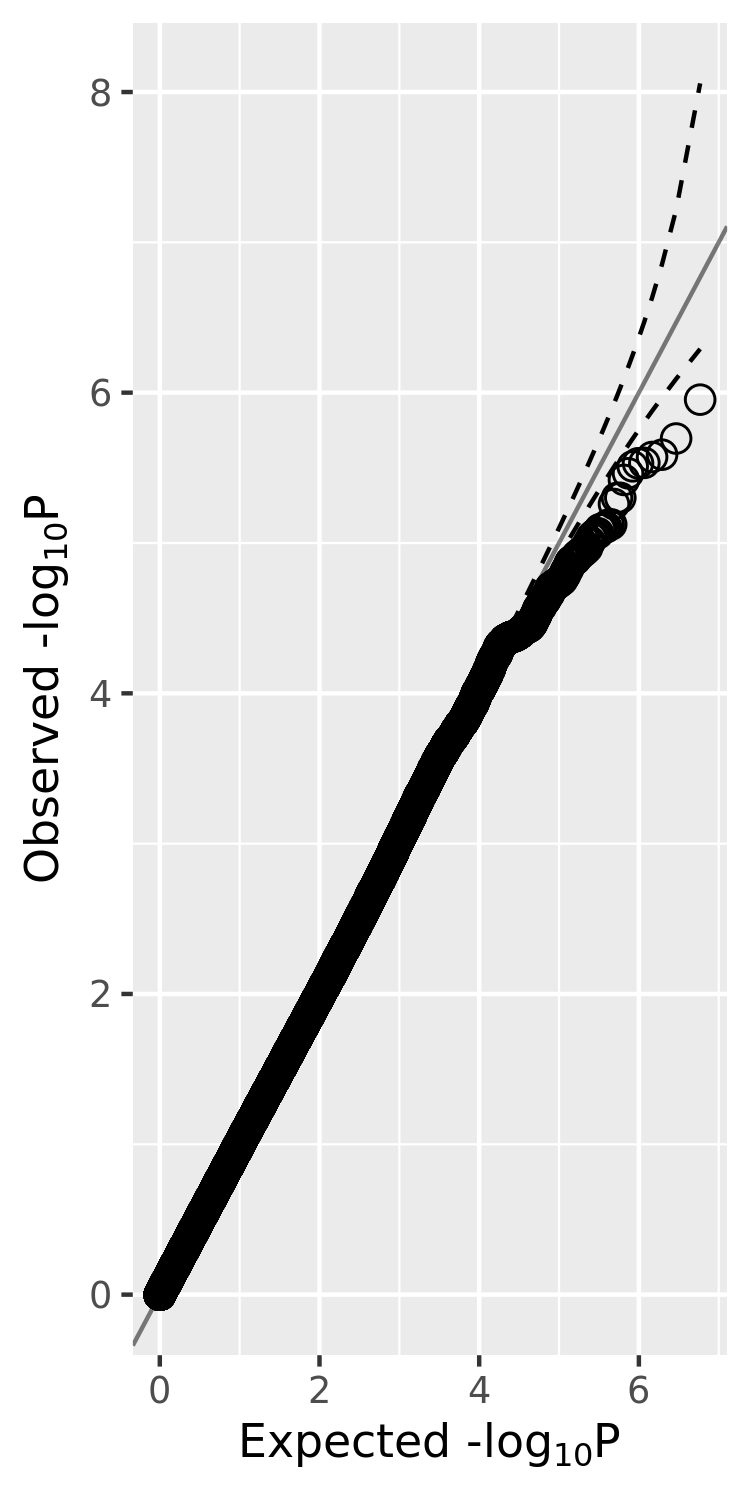 | 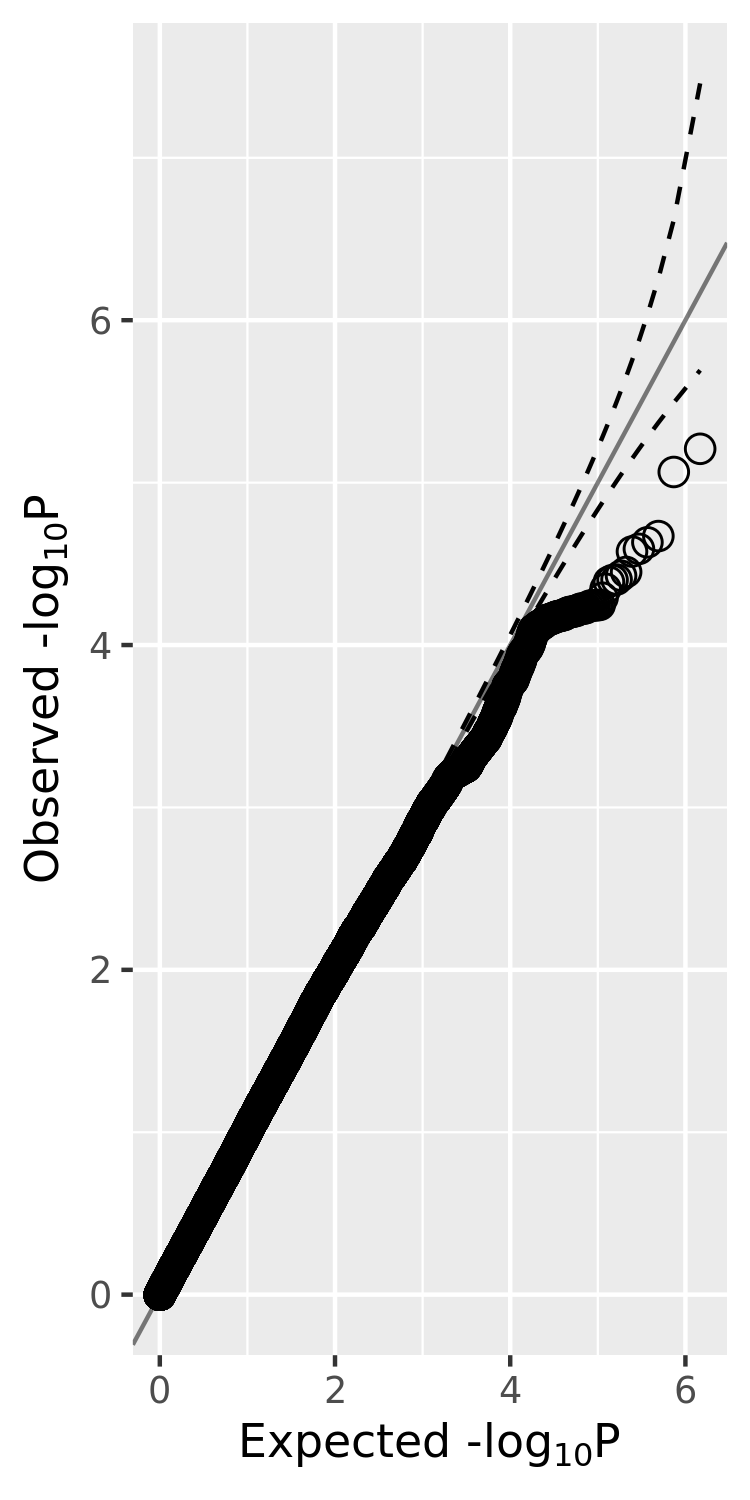 | 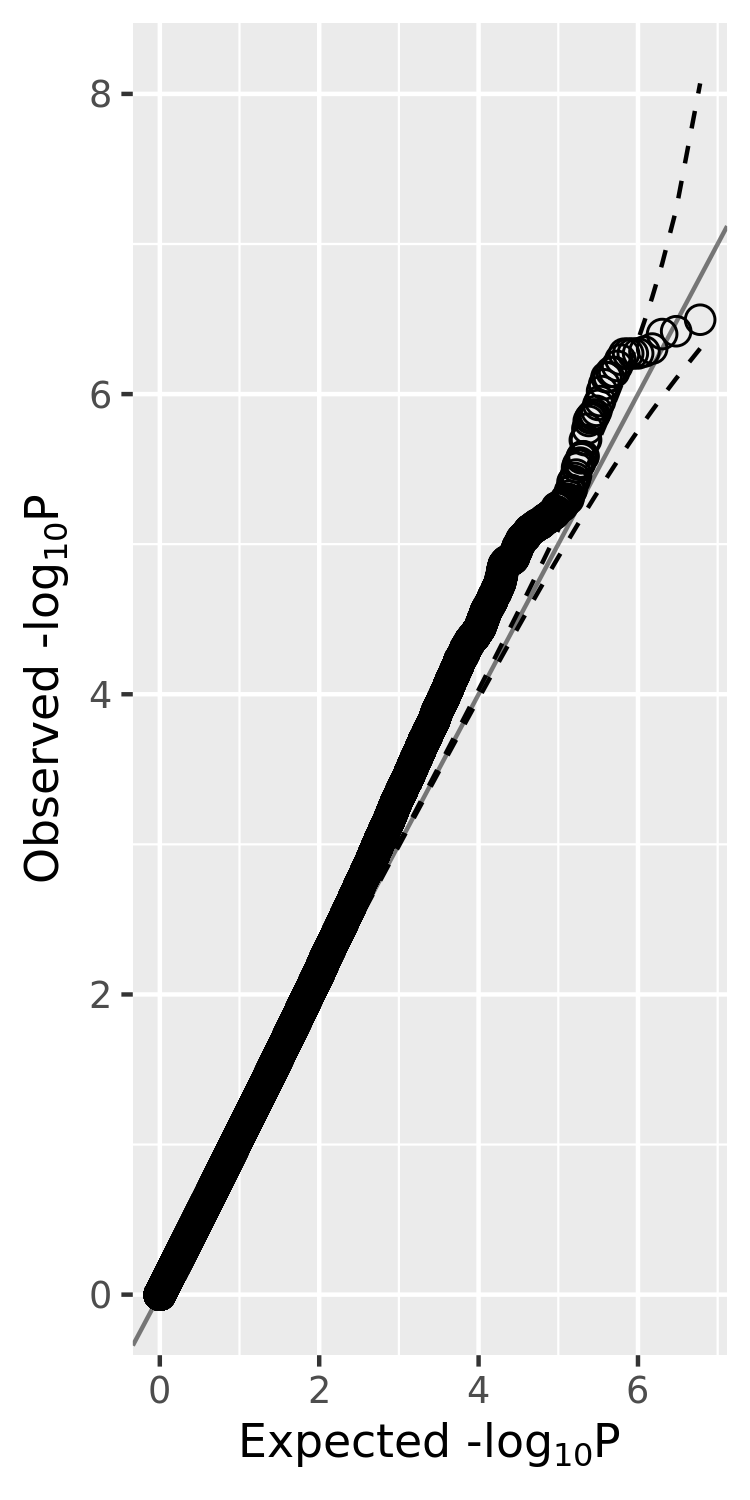 |
| BMI_INV:OE, λ=1.007 | BMI_INV:CM, λ=1.011 | BMI_INV: Meta, λ=1.076 |
| 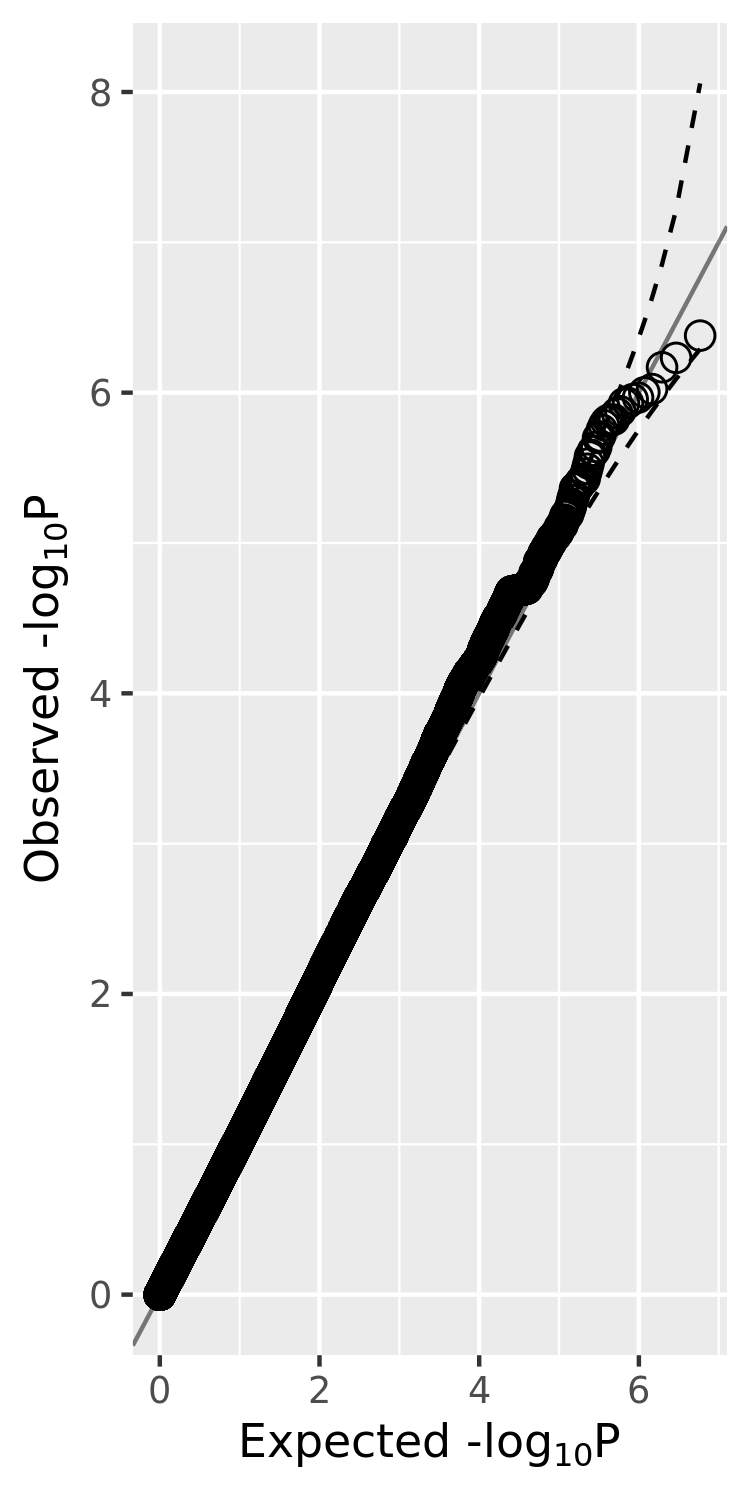 | 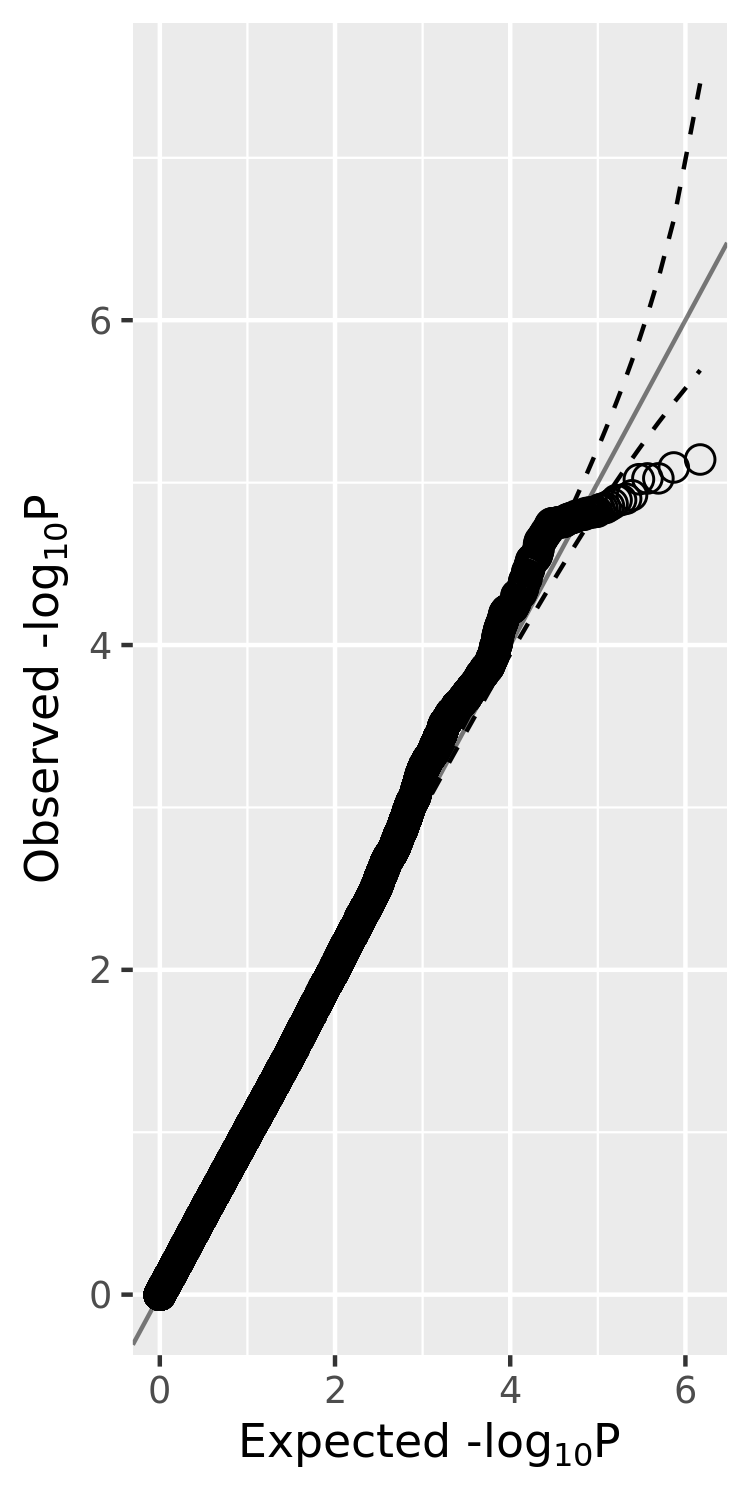 | 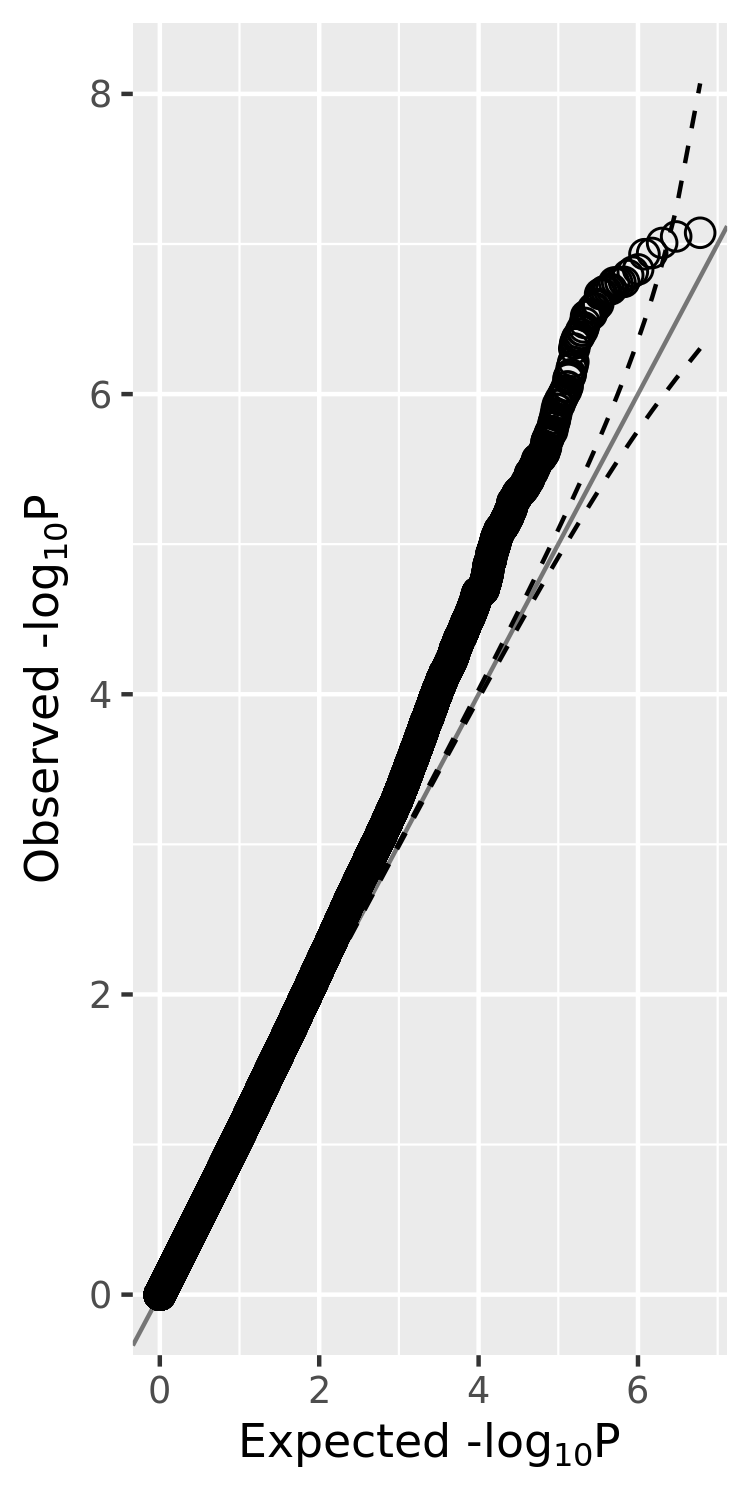 |
| Height_INV:OE, λ=1.051 | Height_INV: CM, λ=1.004 | Height_INV: Meta, λ=1.095 |
| 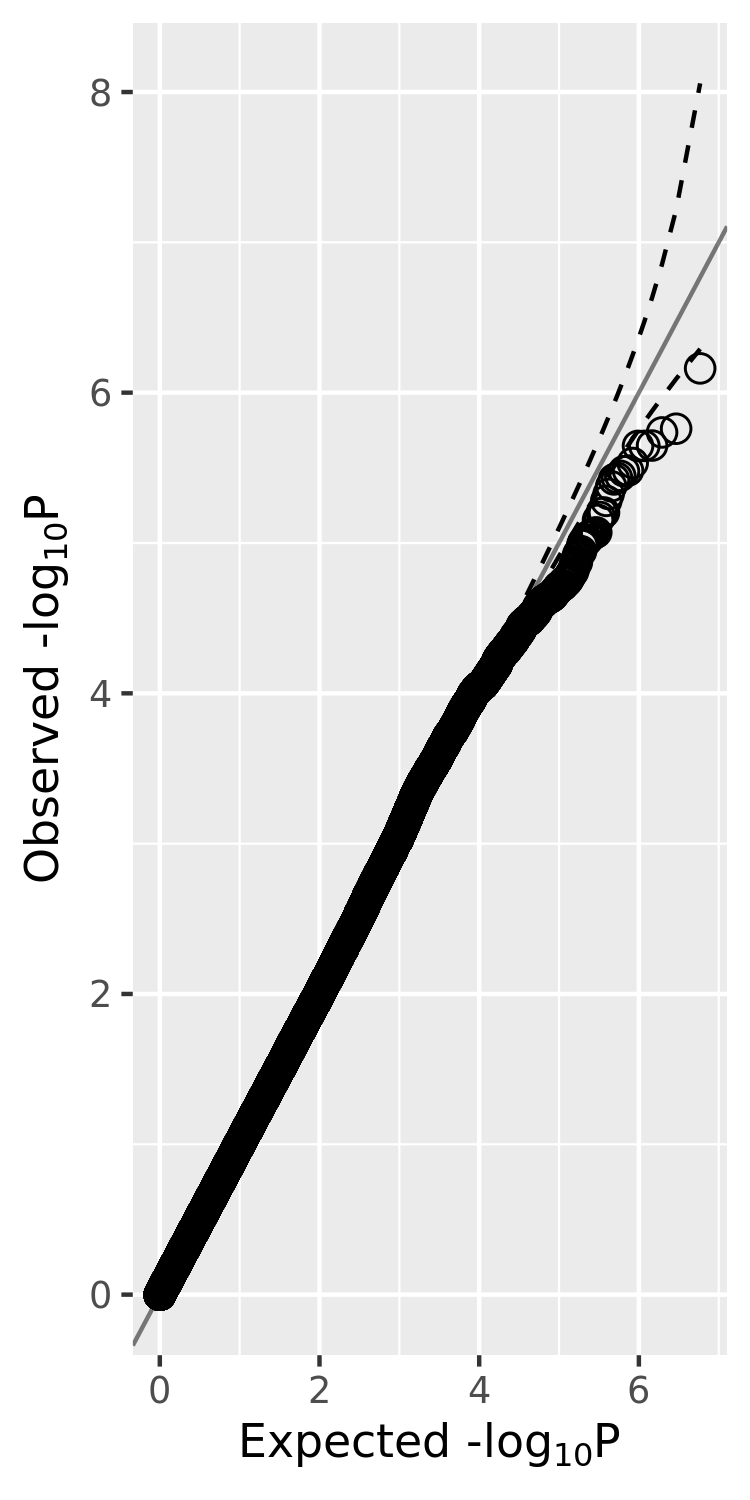 | 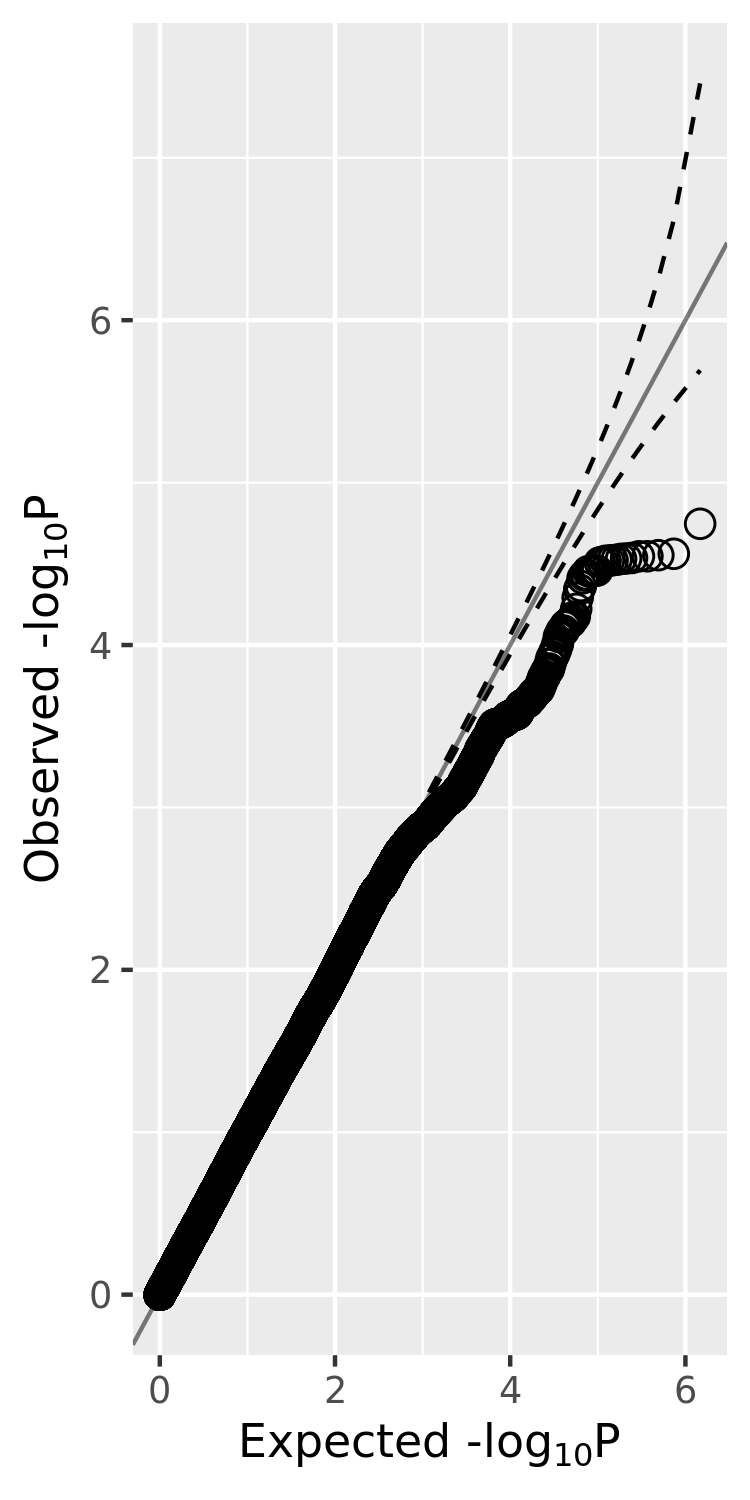 | 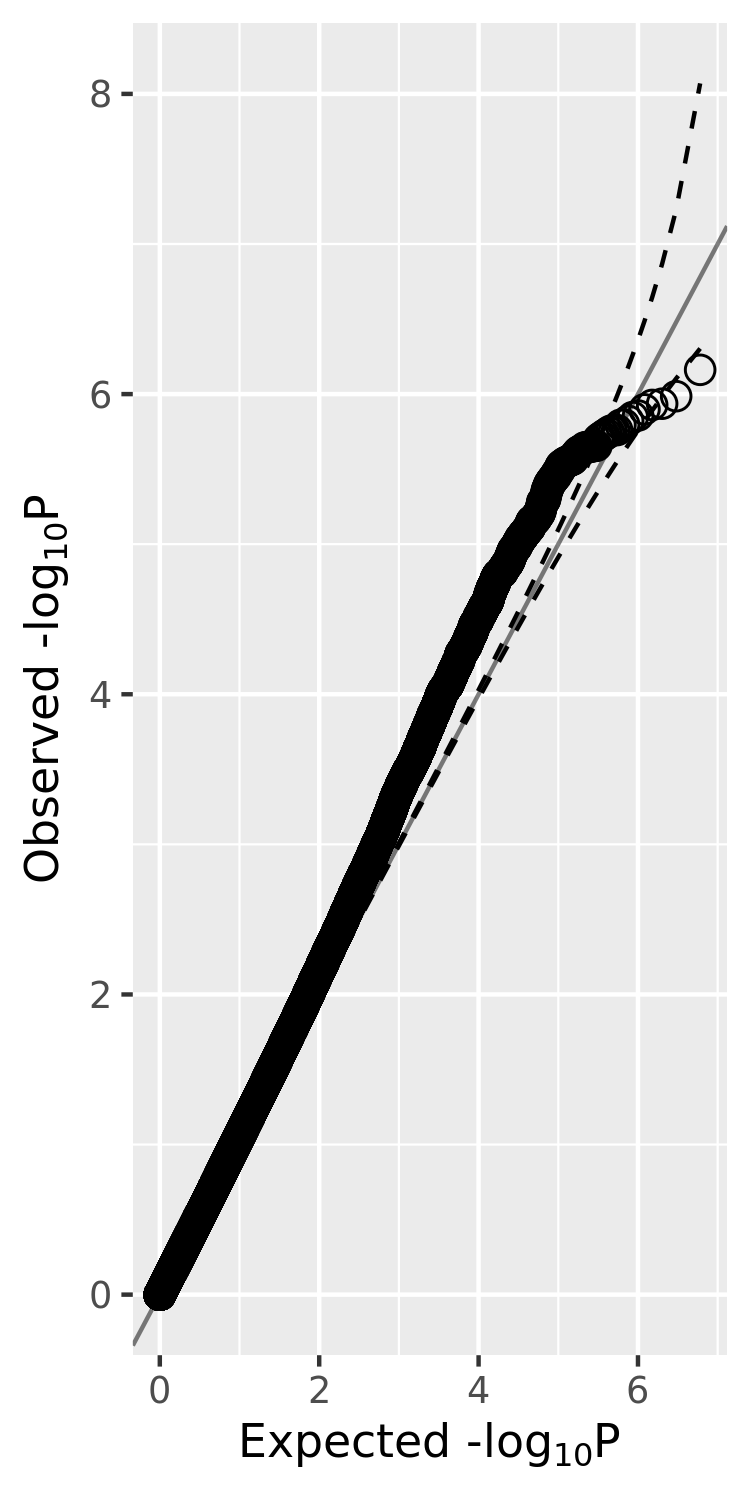 |
| Weight_INV:OE, λ=1.008 | Weight_INV:CM, λ=1.009 | Weight_INV: Meta, λ=1.073 |
| 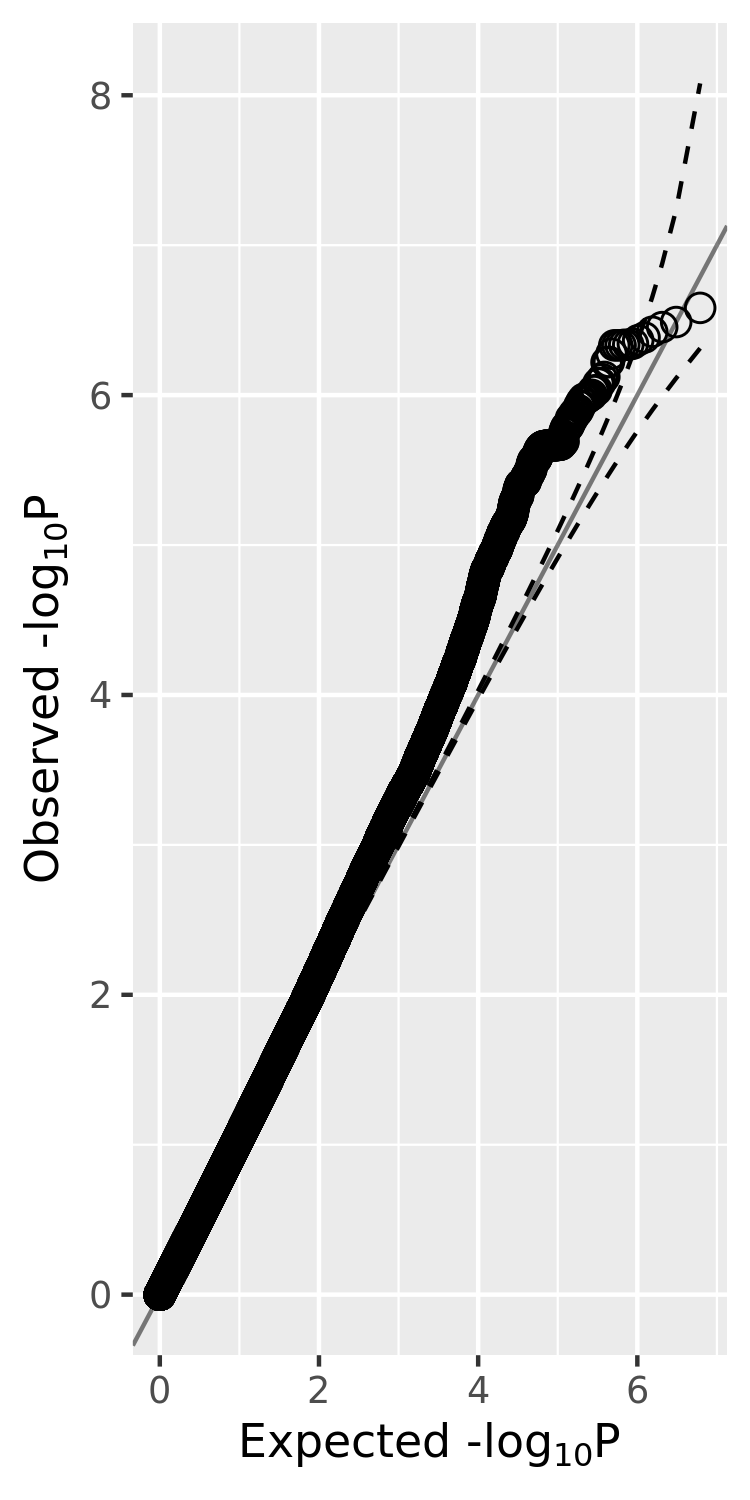 | 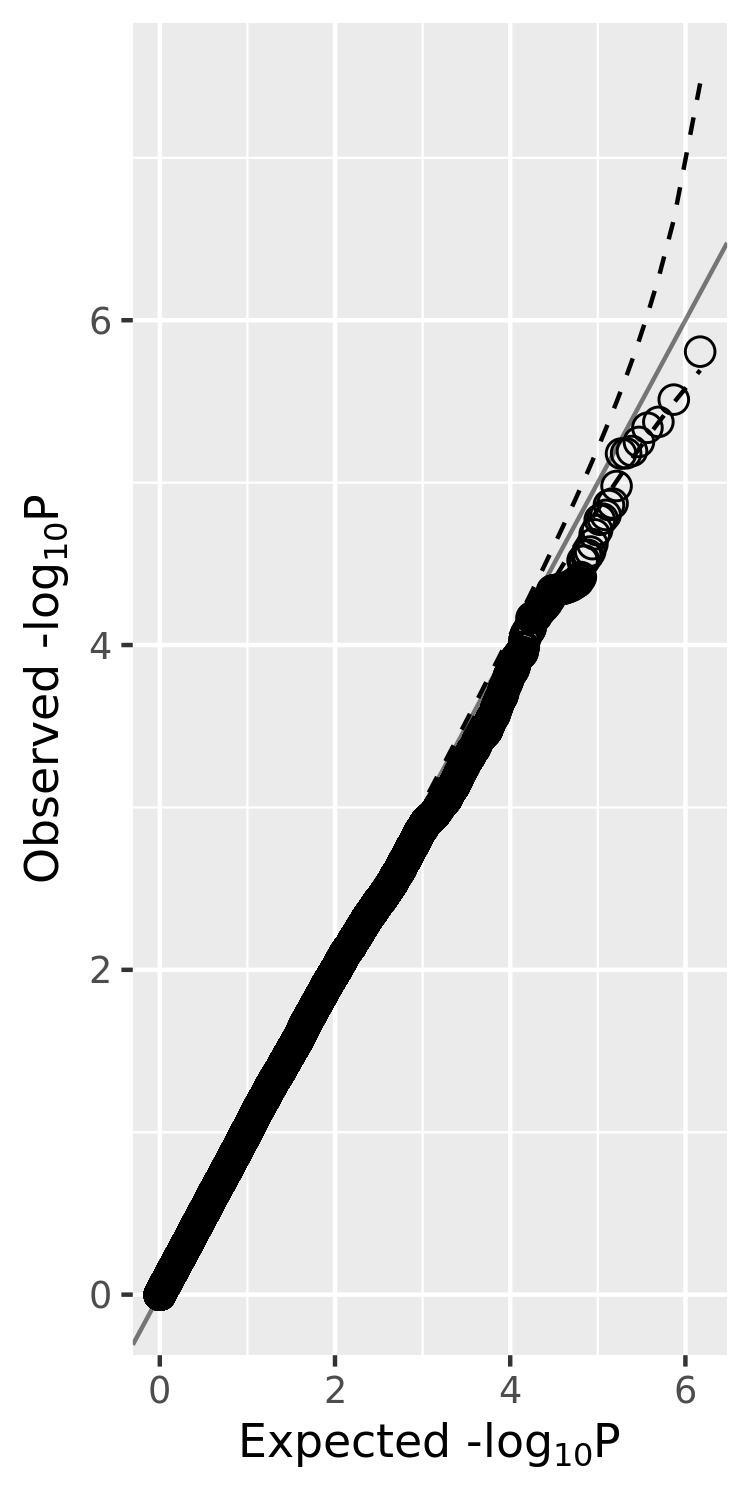 | 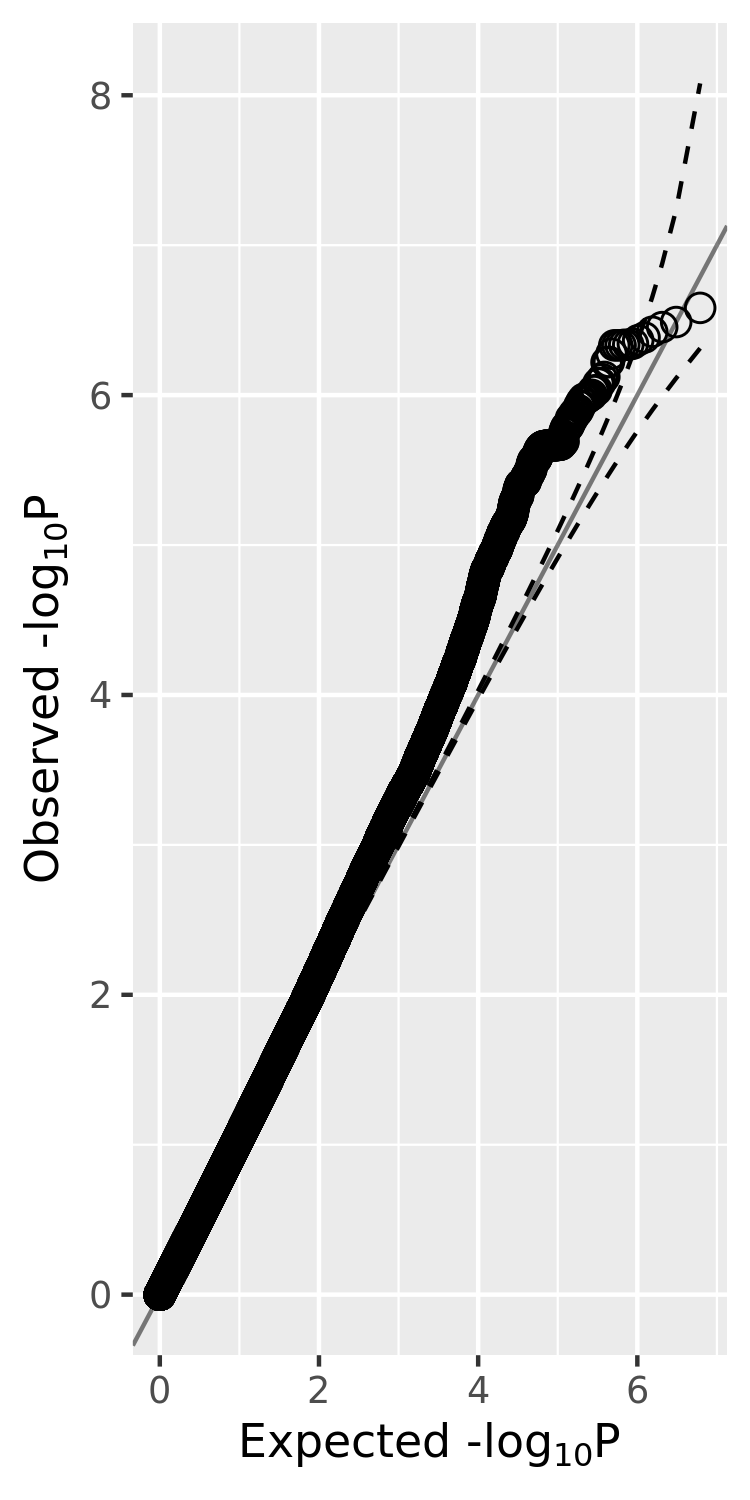 |
| WC_INV:OE, λ=1.004 | WC_INV:CM, λ=1.01 | WC_INV: Meta, λ=1.07 |

**Supplementary Figure S4: Manhattan plots depicting the −log_10_(*p*-values) from the GWAS for all the 13 traits (forming the four classes of anthropometry, glycemic, lipids and blood pressure) in the meta-analysis study.**


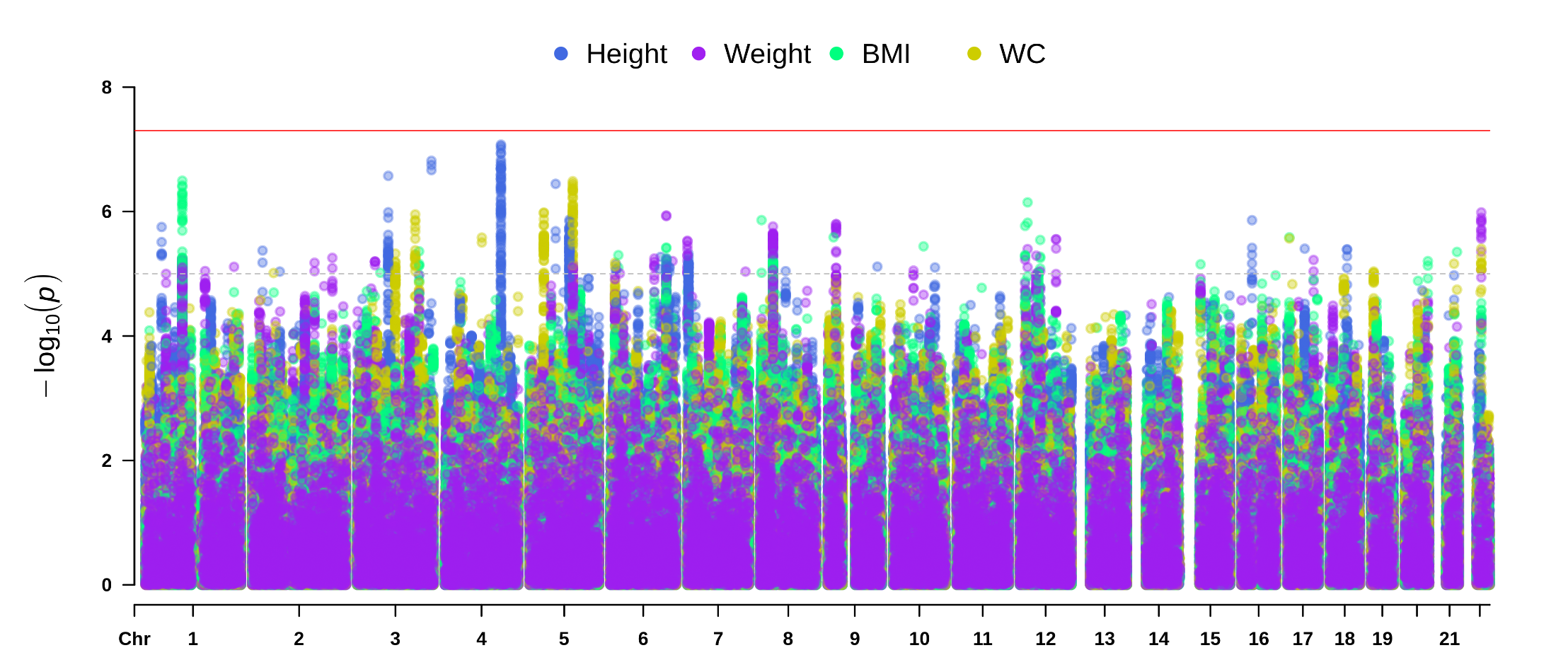


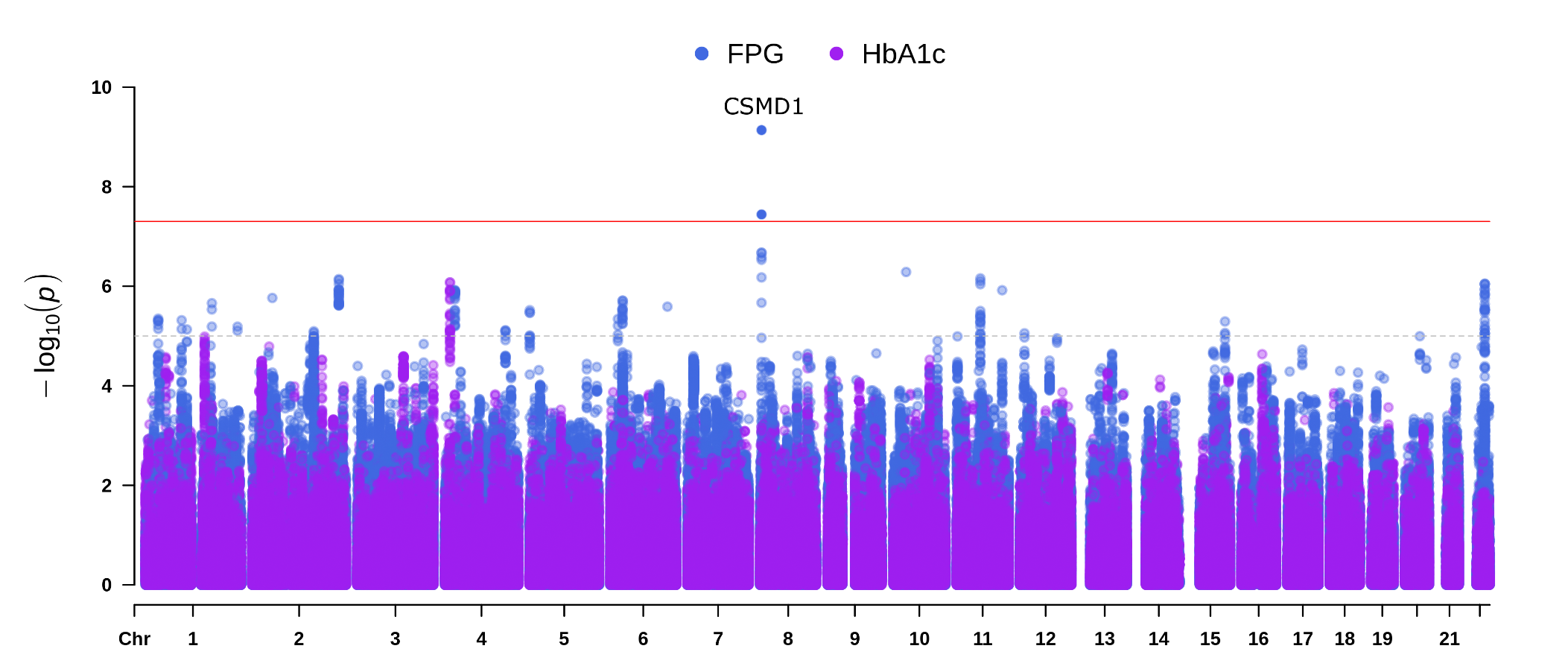


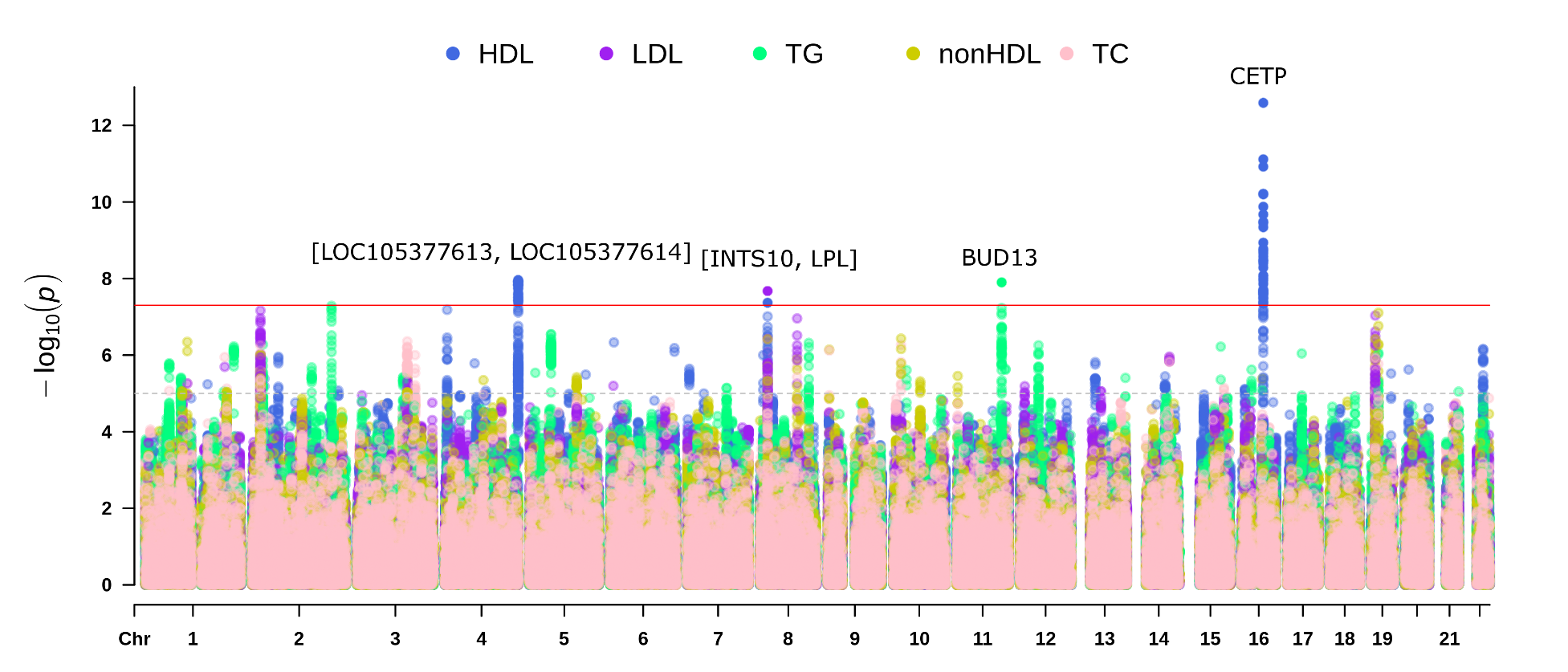


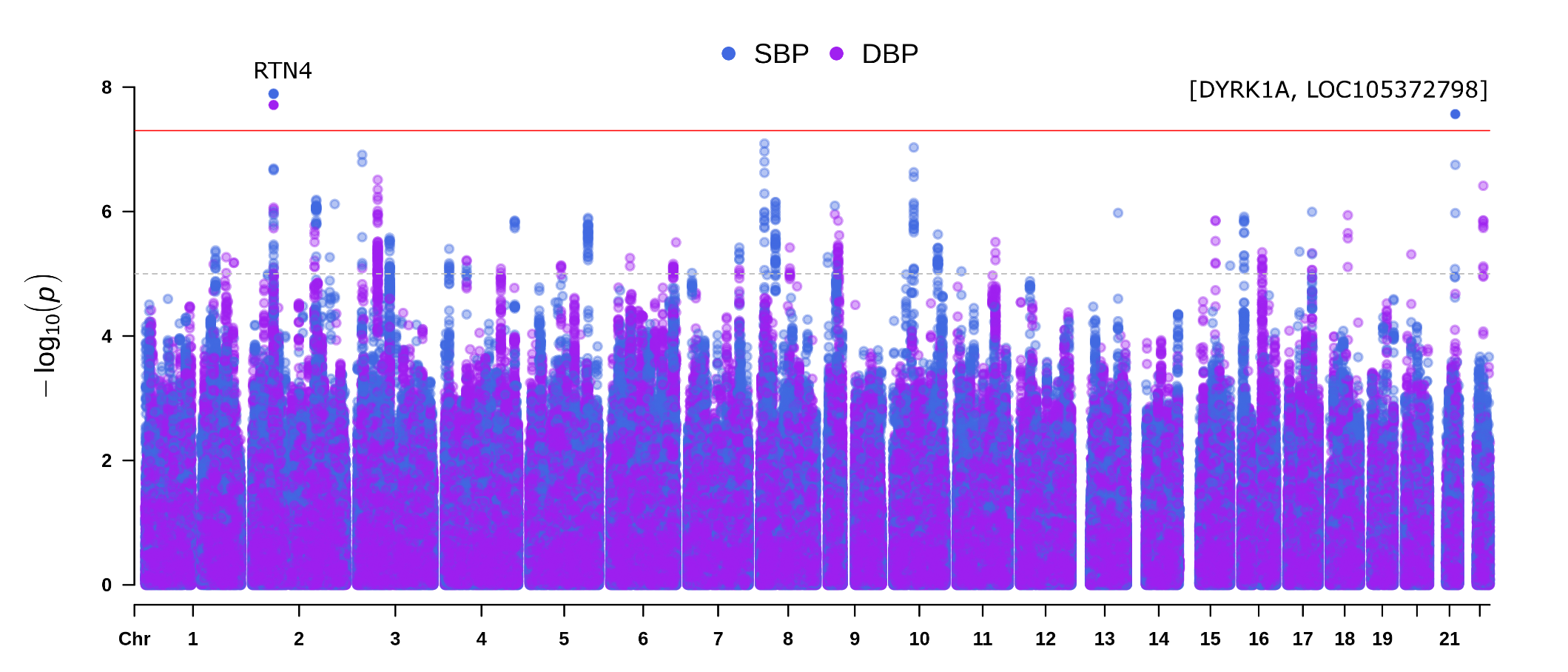


**Supplementary Figure S5. Regional association plots for regions of 500 Kb centered at the risk variants associating with metabolic traits at genome-wide significance in Kuwaiti population.** Associations of the top markers and its LD partners are displayed. The SNPs are color-coded as per the r^2^ value for the SNP with the identified risk variant (Blue dots: r^2^ ≤ 0.2; Purple dots: r^2^ > 0.2 & ≤ 0.4; Green dots: r^2^ > 0.4 & ≤ 0.6; Orange dots: r^2^ > 0.6 & ≤ 0.8; Red dots: r^2^ > 0.8 & ≤ 1.0). The X-axis represents the gene region in physical order; the Y-axis represents −log_10_ P-value of the associations with FPG for all the SNPs. The dashed horizontal line represents a p-value of 5×10^-08^. To generate regional association plot for a SNP-trait association, all the genotyped SNPs (passing the QC analyses) from a region of around 500 Kb centered on the SNP were tested for association with the trait; the resultant statistics and the SNPs were displayed in the regional association plot. Region-plot tool (https://github.com/pgxcentre/region-plot) was used to produce regional plots.


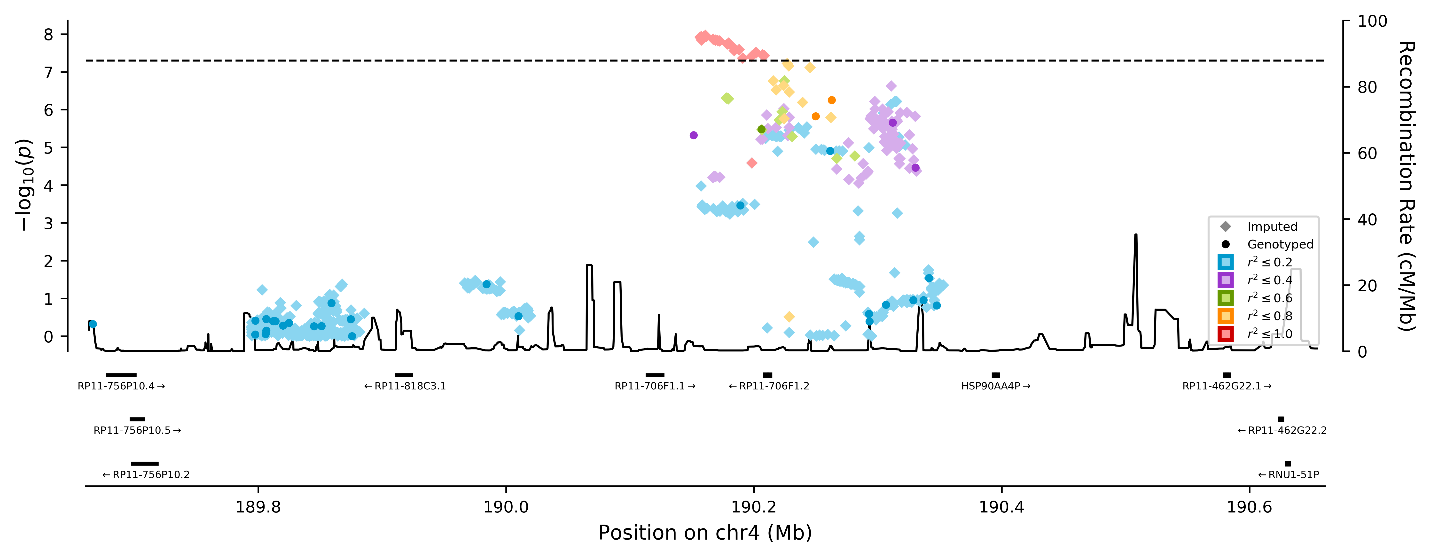


Regional plot for chr4:189660826_190660826_HDL_INV (encompassing the many SNPs - rs11132637, rs35387314, rs6846011, rs34453299, rs561984720, rs9790417, rs11942078, rs35668405, rs35602882, rs35266160, rs11721957, rs35466621, rs35699925, rs34329457, rs13147669, rs76018028, rs35192598, rs55944922, rs13141596, rs13120111, rs13119288, rs36021514, rs34728227, rs35127507, rs34706863 - all associating with HDL_INV at genome-wide significance).


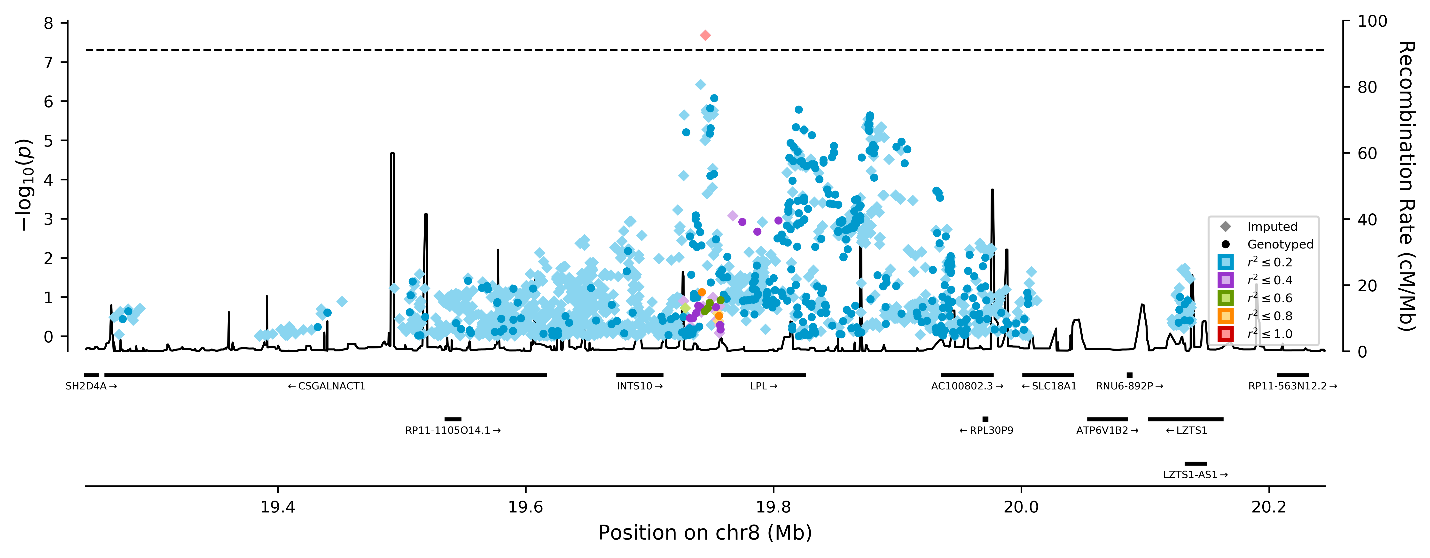


Regional plot for chr8:19245039_20245089_LDL_INV (encompassing the SNP rs10635970 associating with LDL_INV at genome-wide significance).


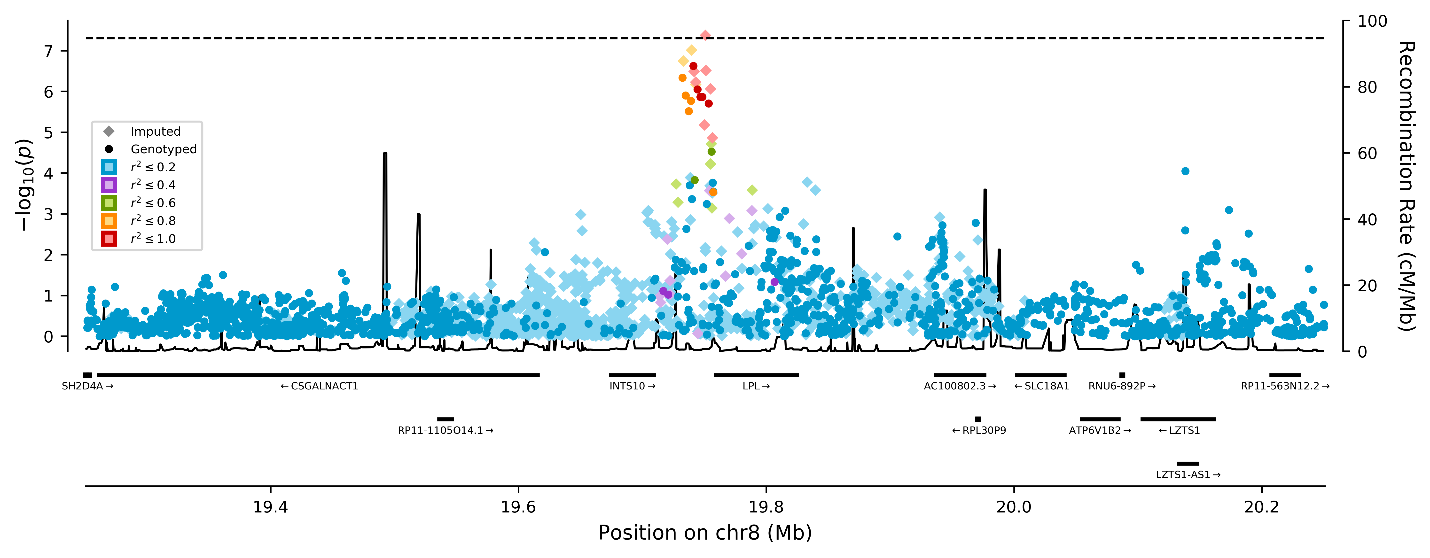


Regional plot for chr8:19245039_20245089_HDL_INV (encompassing the SNP rs112861901 associating with HDL_INV at genome-wide significance).


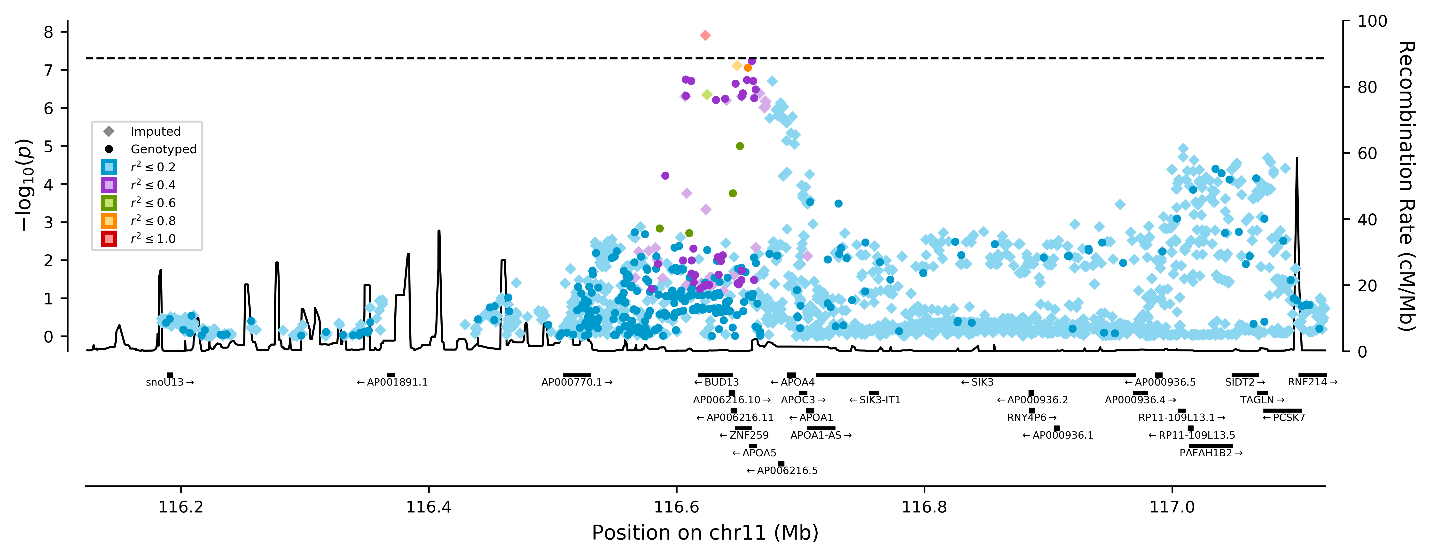


Regional plot for chr11:116123213_117123213_TG_INV (encompassing the SNP rs66505542 associating with TG_INV at genome-wide significance).


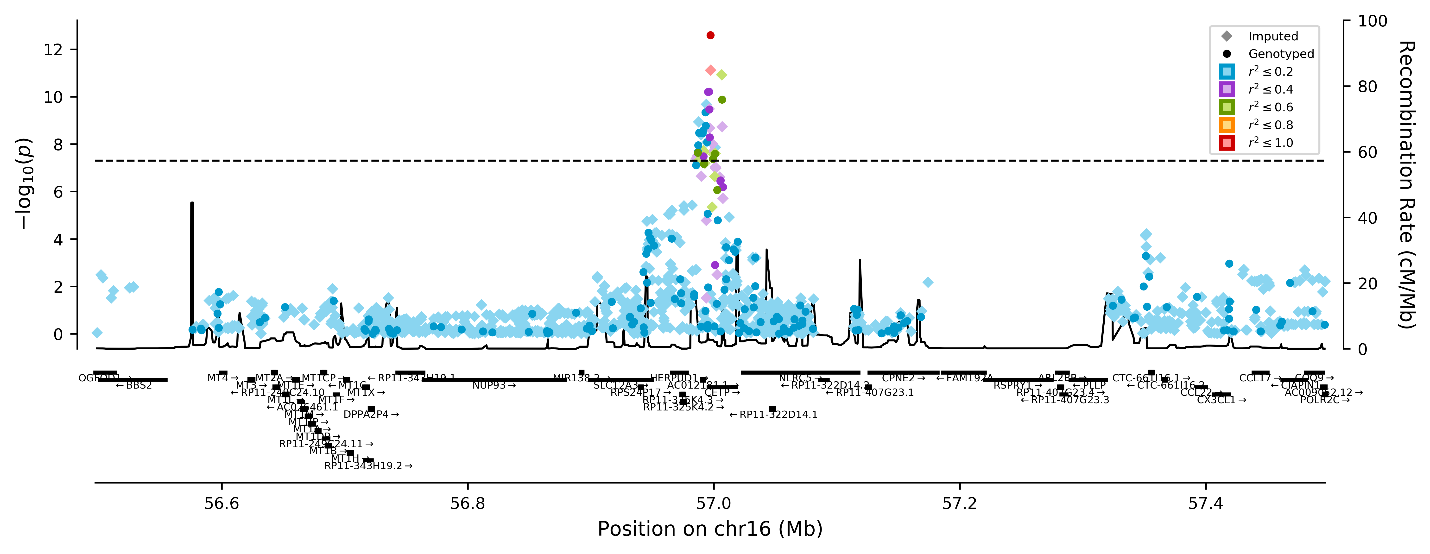


Regional plot for chr16:56497233_57497233_HDL_INV (encompassing the many SNPs - rs289713_A, rs7499892_T, rs11076175_G, rs200751500_A, rs12720908_TCACA, rs12720922_A, rs17231569_C, rs11508026_T, rs7203984_C, rs12720926_G, rs5817082_CA, rs1864163_A, rs34620476_A, rs34145065_G, rs708272_A, rs711752_A, rs3816117_C, rs1800775_A, rs17231506_T, rs36229491_TA, rs821840_G, rs3764261_A, rs12149545_A, rs201825234_A, rs6499862_A, rs6499861_G, rs183130_T, rs247617_A, rs247616_T, rs2399597_A, rs173539_T, rs56228609_T, rs56156922_C, rs12446515_T, rs7205692_G, rs7203286 rs11862052_T - all associating with HDL_INV at genome-wide significance).


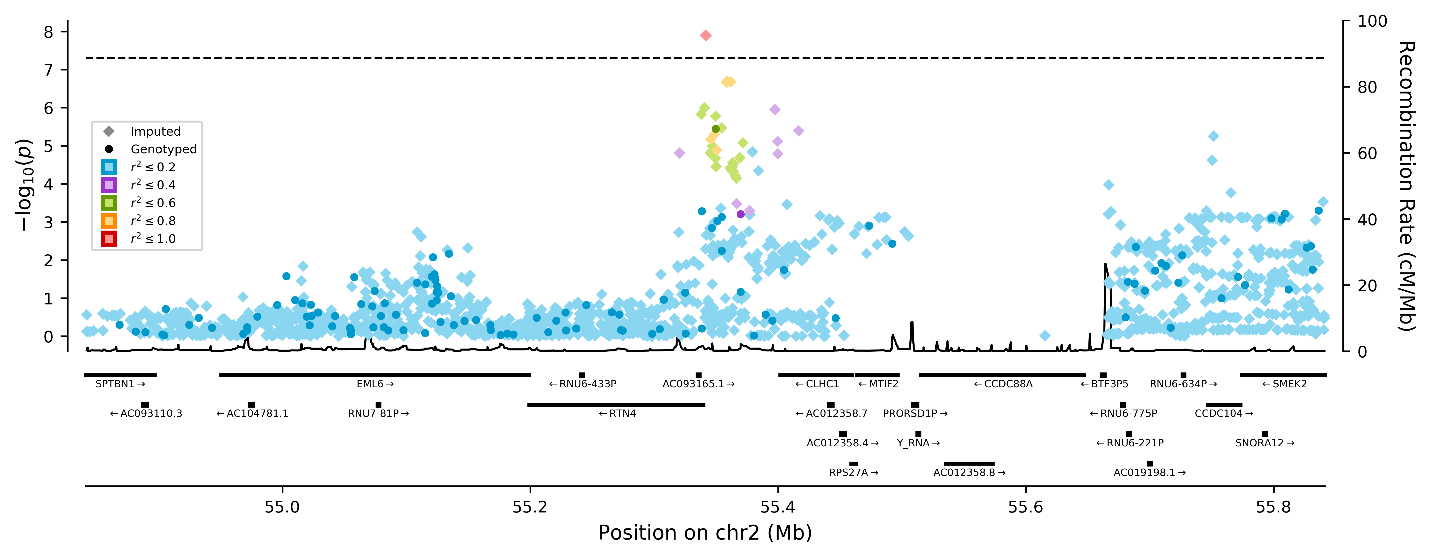


Regional plot for chr2:54841367-55841367_SBP_INV (encompassing the SNP rs2920844 associating with SBP_INV at genome-wide significance).


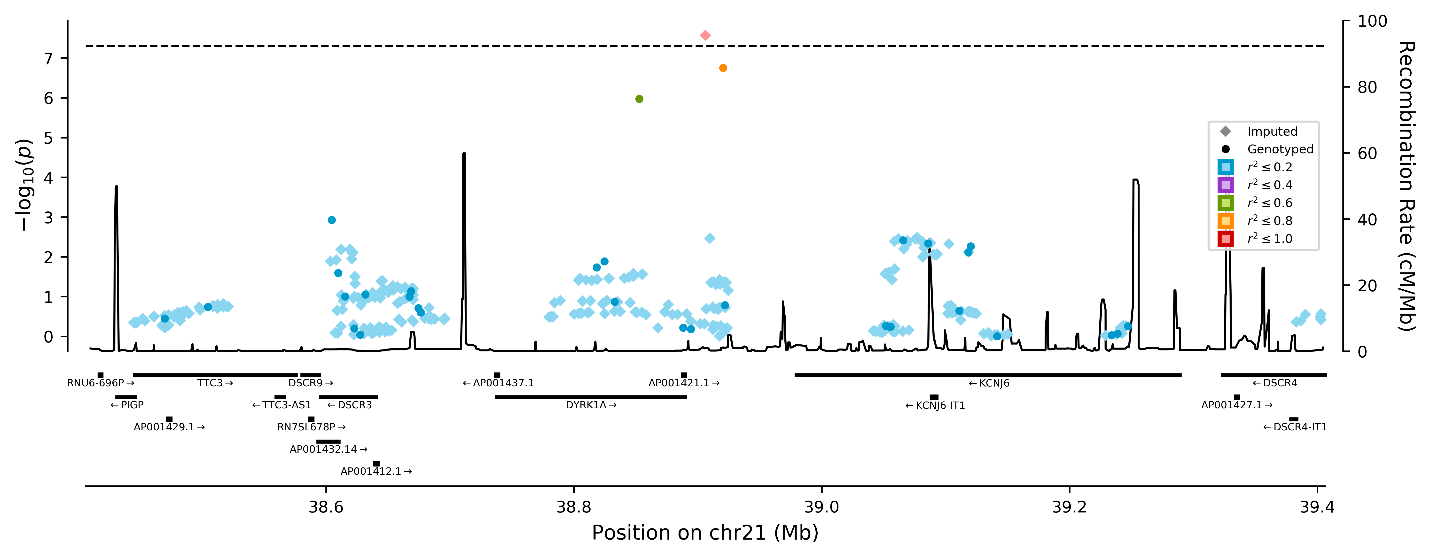


Regional plot for chr21:38406071-39406071_SBP_INV (encompassing the SNP rs2835788 associating with SBP_INV at genome-wide significance).


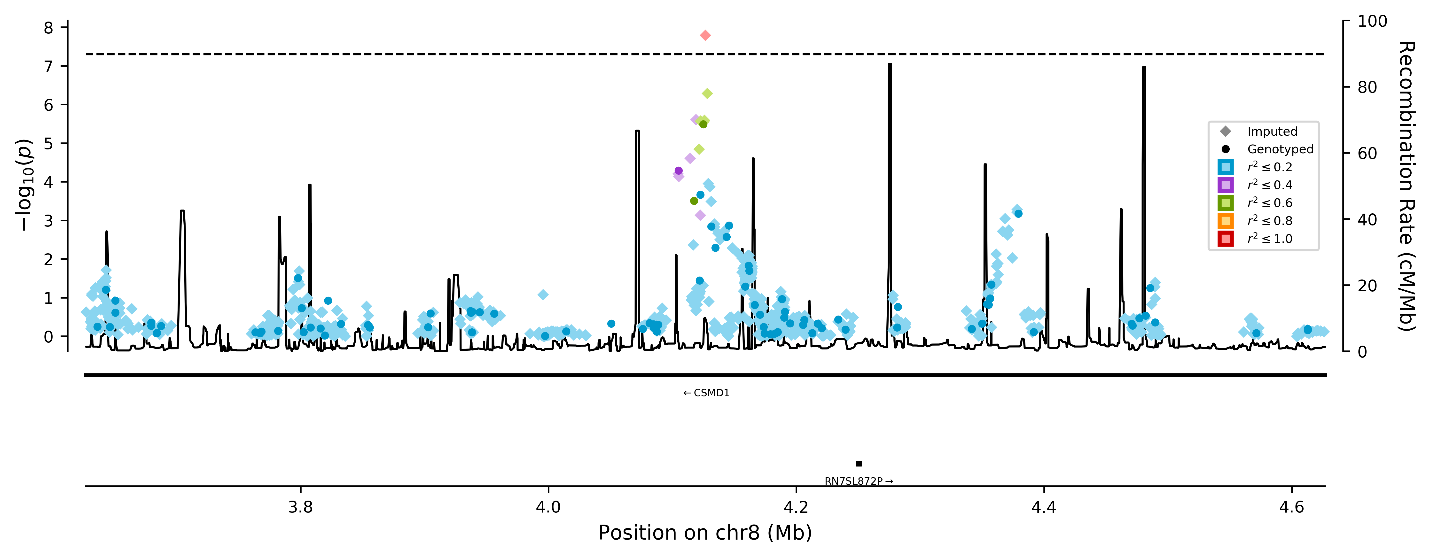


Regional plot for chr8:3626701-4626701_FPG_INV (encompassing the SNP rs7838666 associating with FPG_INV at genome-wide significance).


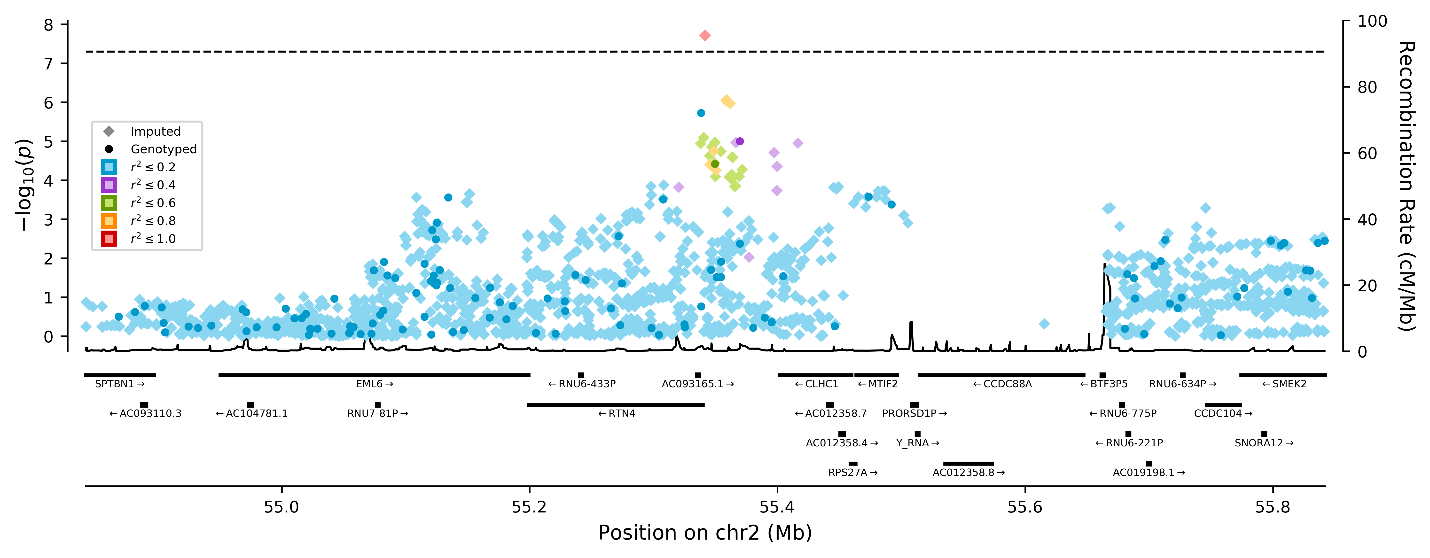


Regional plot for chr2:54841367-55841367_DBP_INV (encompassing the SNP rs2920844 associating with DBP_INV at genome-wide significance).


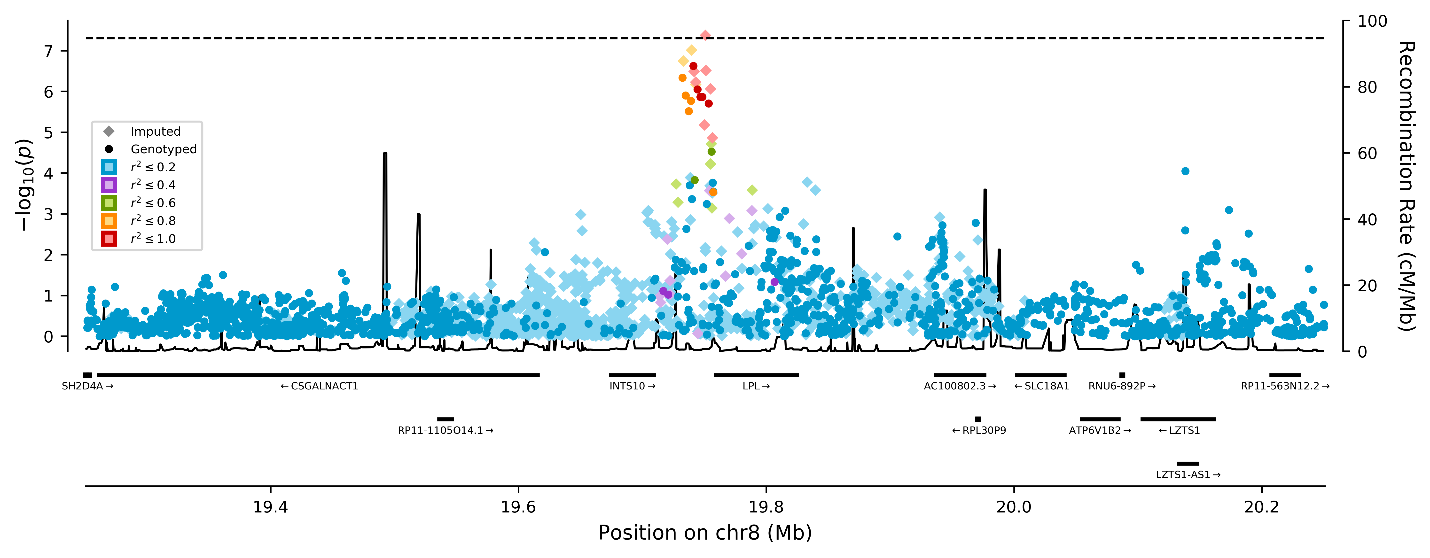


Regional plot for chr8_19245039-20245039_LDL_INV (encompassing the SNP rs10635970 associating with LDL_INV at genome-wide significance)

**Supplementary Figure S6: Mapping of the variants associated with the 13 metabolic traits in our meta-analysis to different classes of metabolic traits in GWAS Catalog. A.** Shared effect of anthropometric variants on other traits; **B.** Shared effect of glycemic variants on other traits; **C.** Shared effect of blood pressure variants on other traits; **D.** Shared effect of lipid variants on other traits.

.
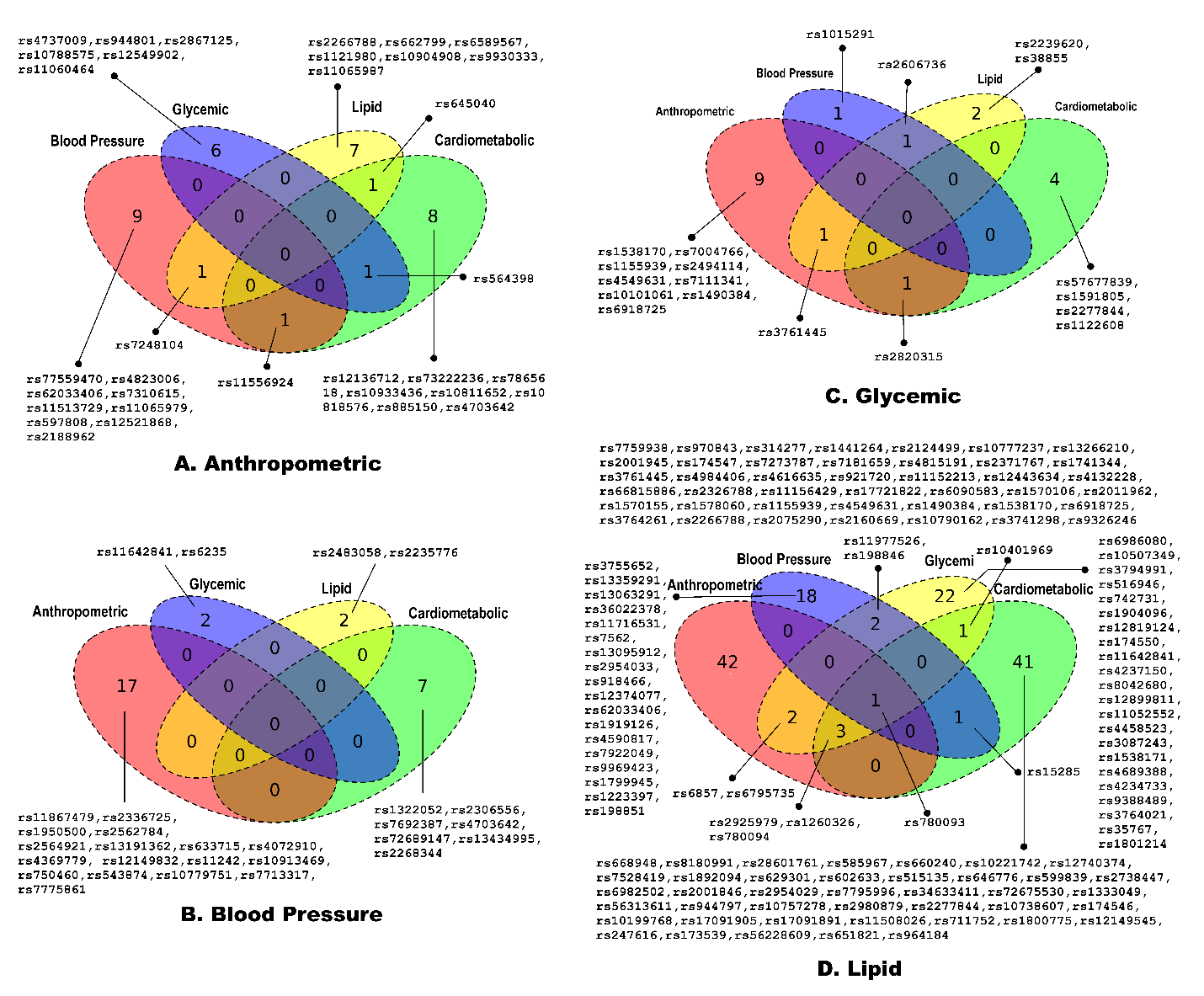


**Supplementary Figure S7: Mapping of the genes that harbour the variants associated with the 13 metabolic traits in our meta-analysis to different classes of metabolic traits in GWAS Catalog. A.** Shared effect of anthropometric genes on other traits; **B.** shared effect of glycemic genes on other traits; **C.** shared effect of blood pressure genes on other traits; **D.** shared effect of lipid genes on other traits.


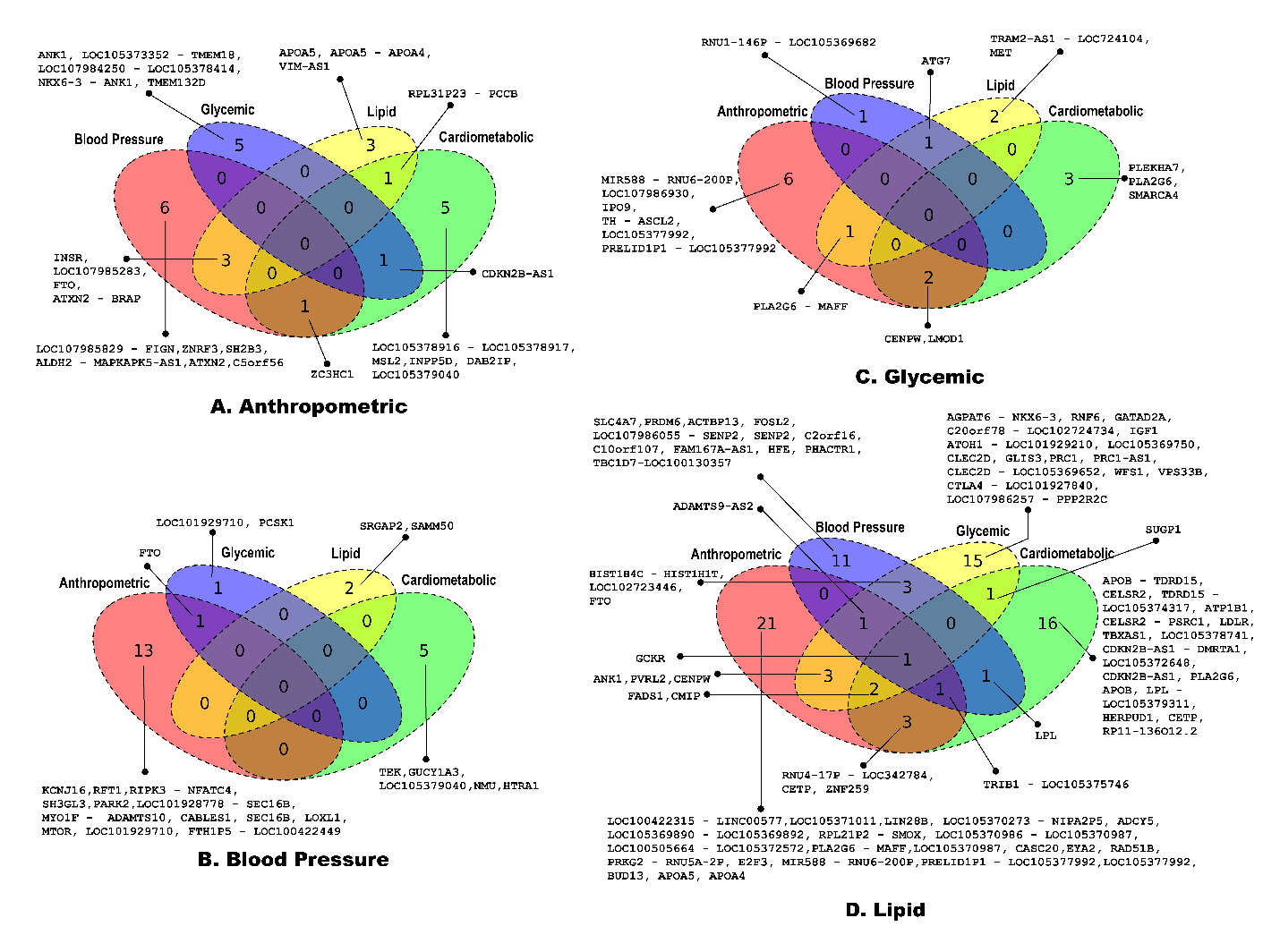


**Supplementary Figure S8: Ethnic transferability of association signals for metabolic traits among populations.** The figure presents the counts of transferable association signals (at the level of genes) from each global population to Arab population through ‘Direct’ **(A)** or ‘Indirect’ **(B)** relationships.


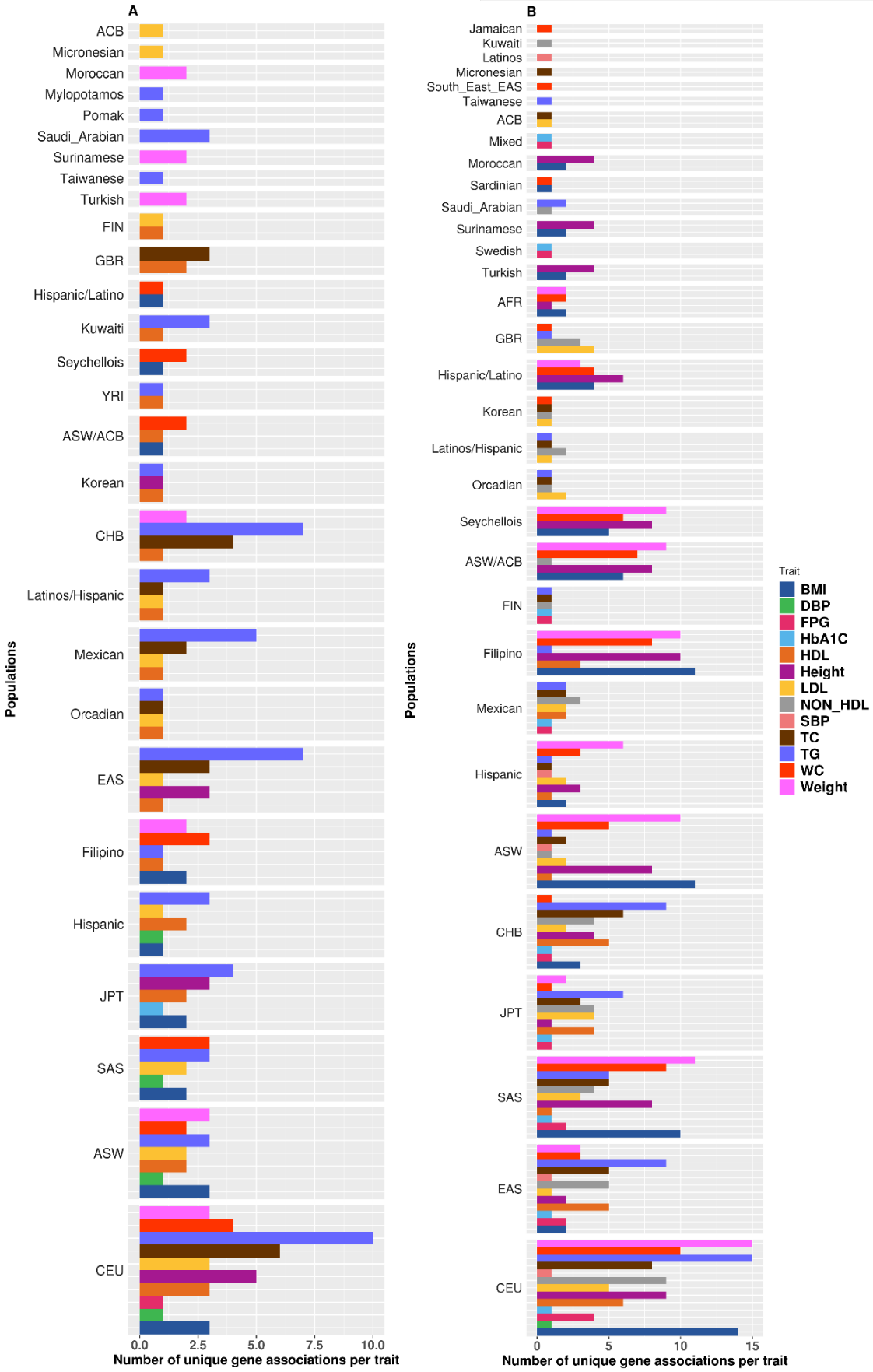


| **Supplementary Figure S9: Estimation of power and sample size for the variants of different effect sizes and MAF’s for the different traits.** The left panel considers established variants replicated in our study (at either borderline or suggestive p-values**)** and the right panel considers established variants non-replicated in our study. | |
| --- | --- |
| 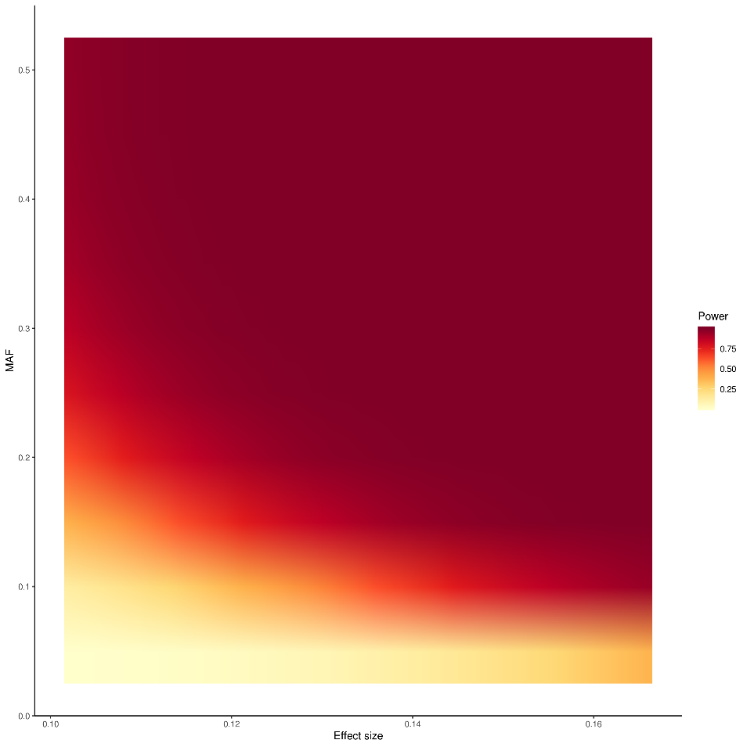 | 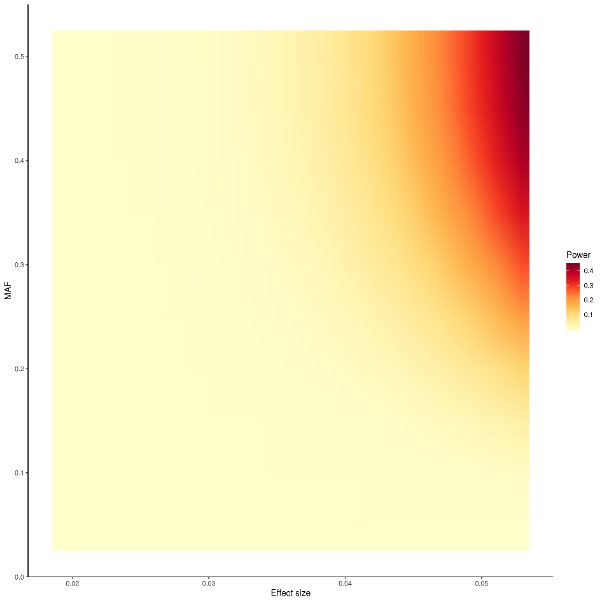 |
| BMI: Study variants at sample size 10K | BMI: Non-replicated variants at sample size 20K |
| 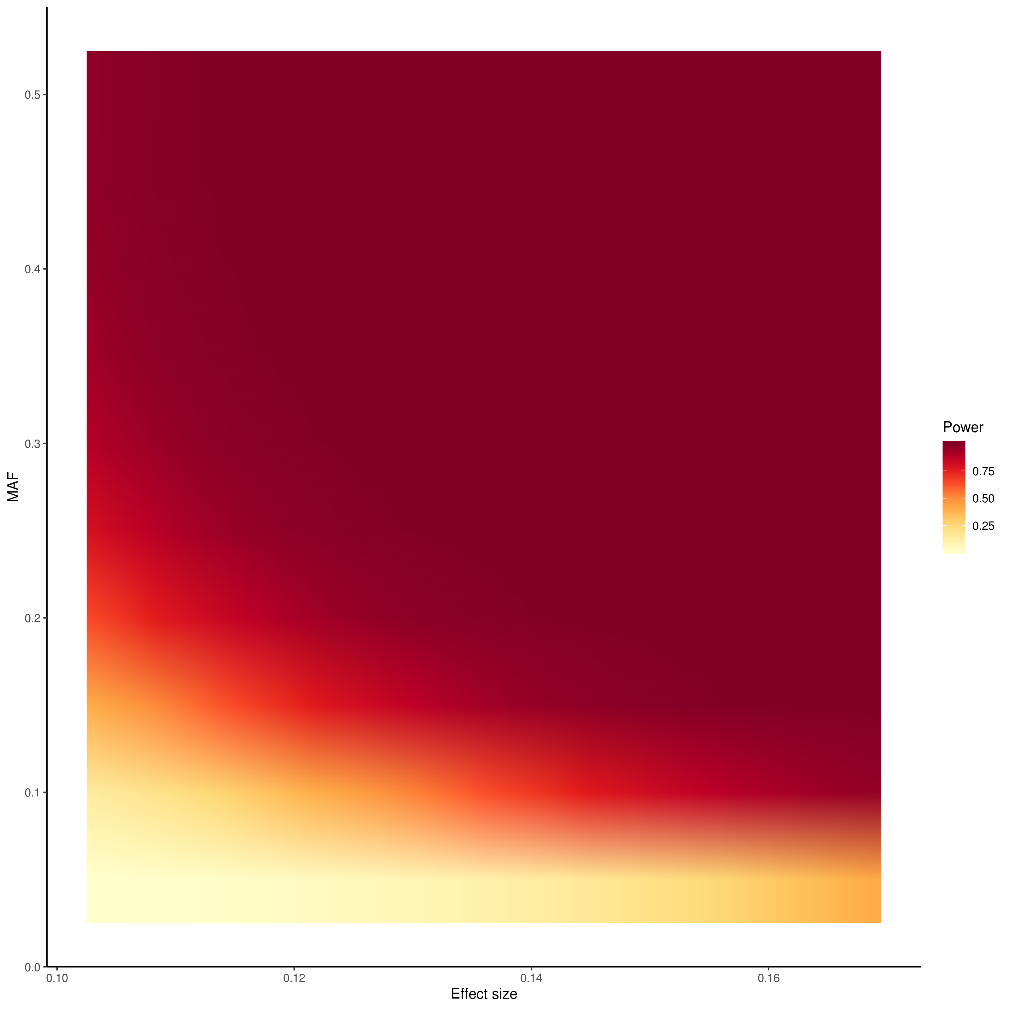 | 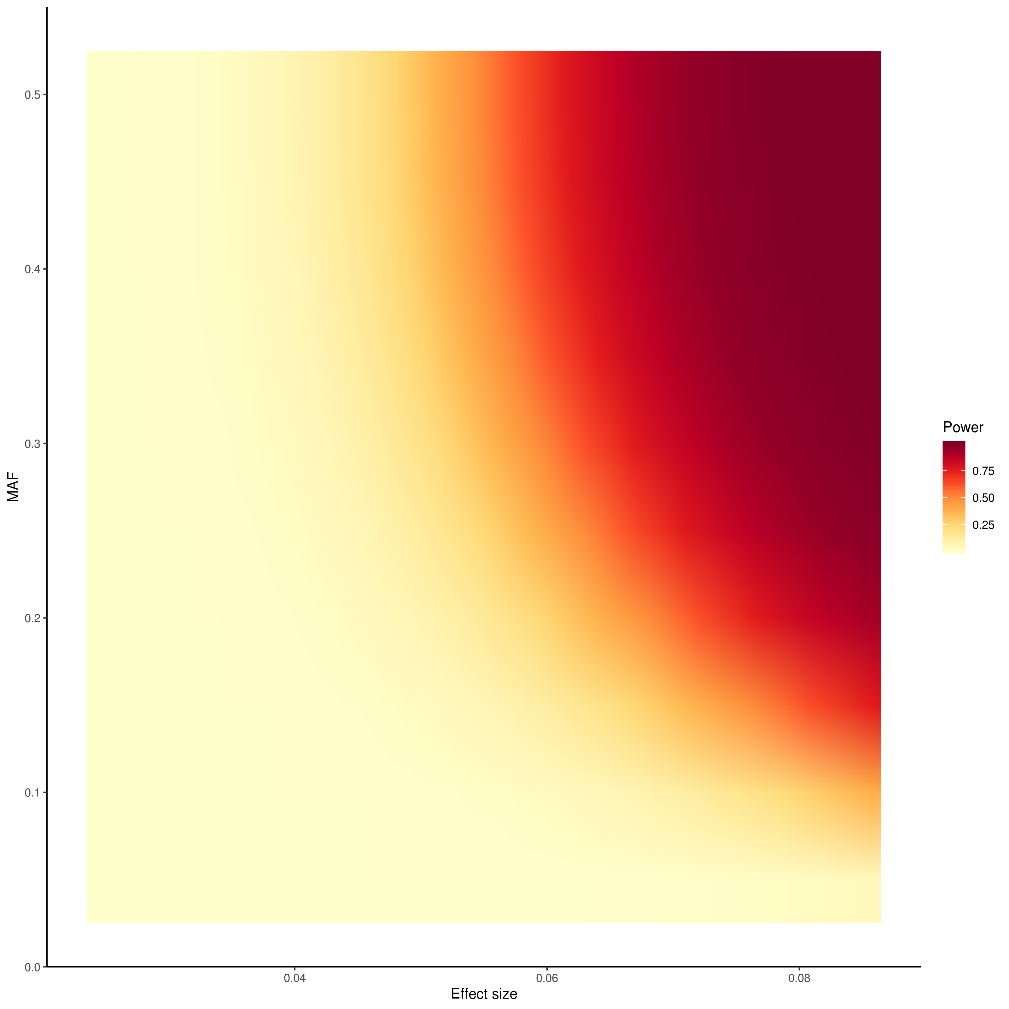 |
| DBP: Study variants at sample size 10K | DBP: Non-replicated variants at sample size 20K |
| 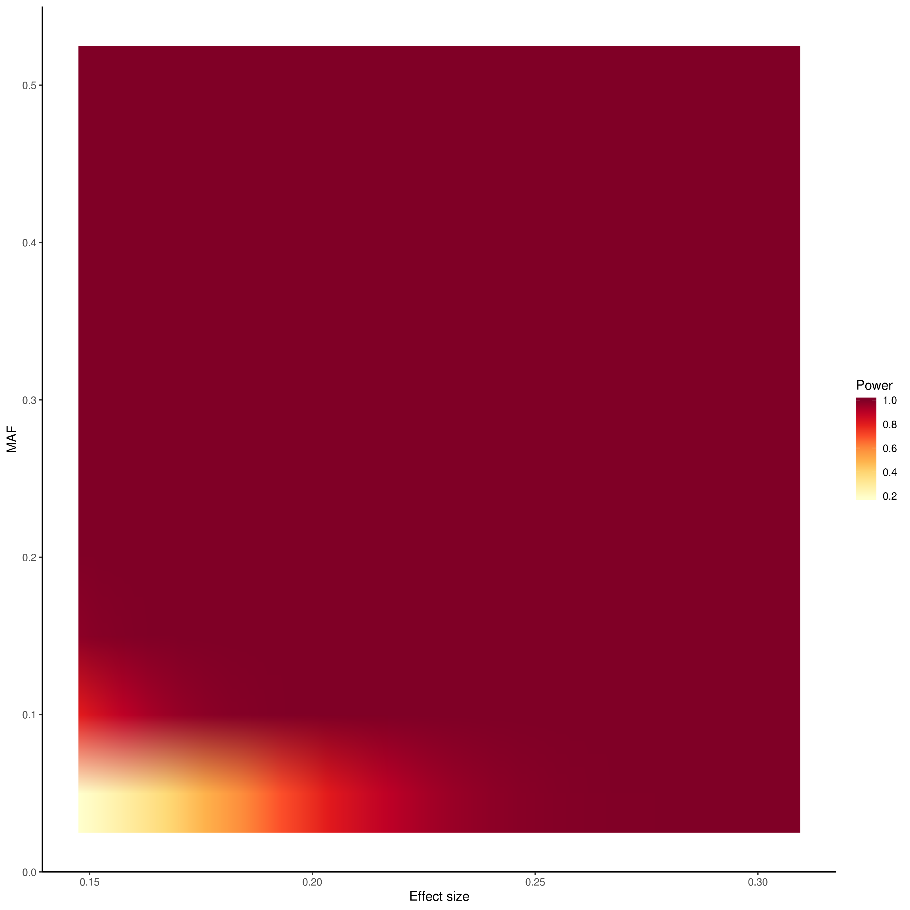 | 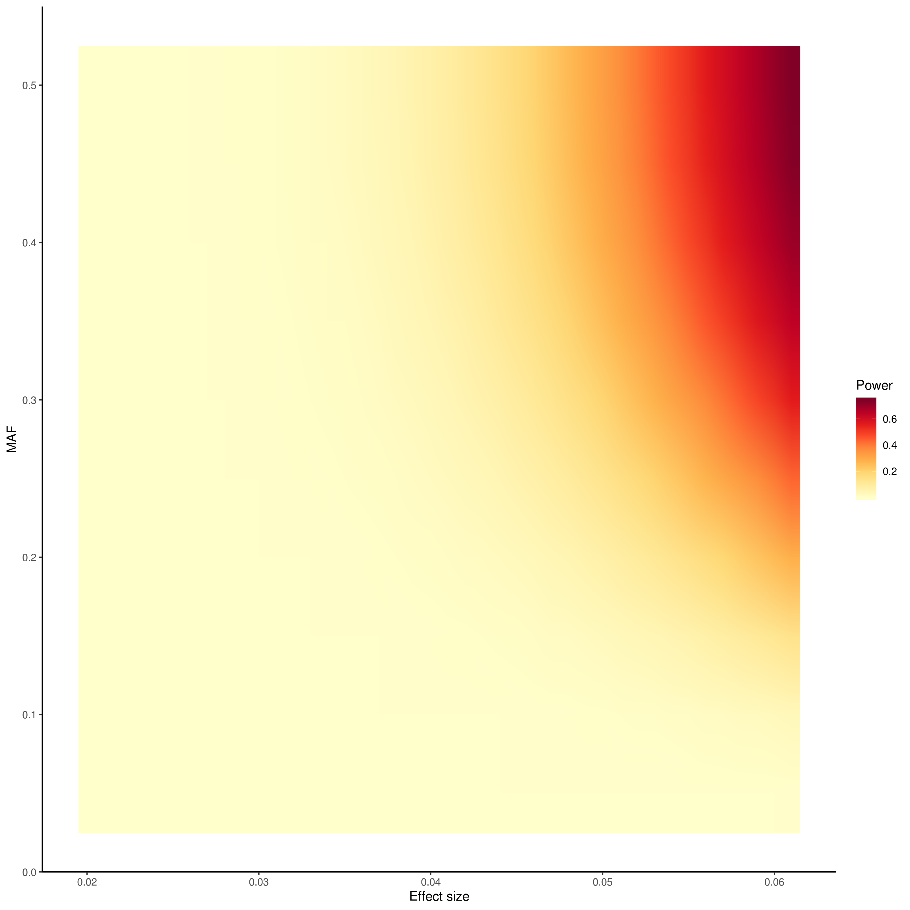 |
| FPG: Study variants at sample size 10K | FPG: Non-replicated variants at sample size 20K |
| 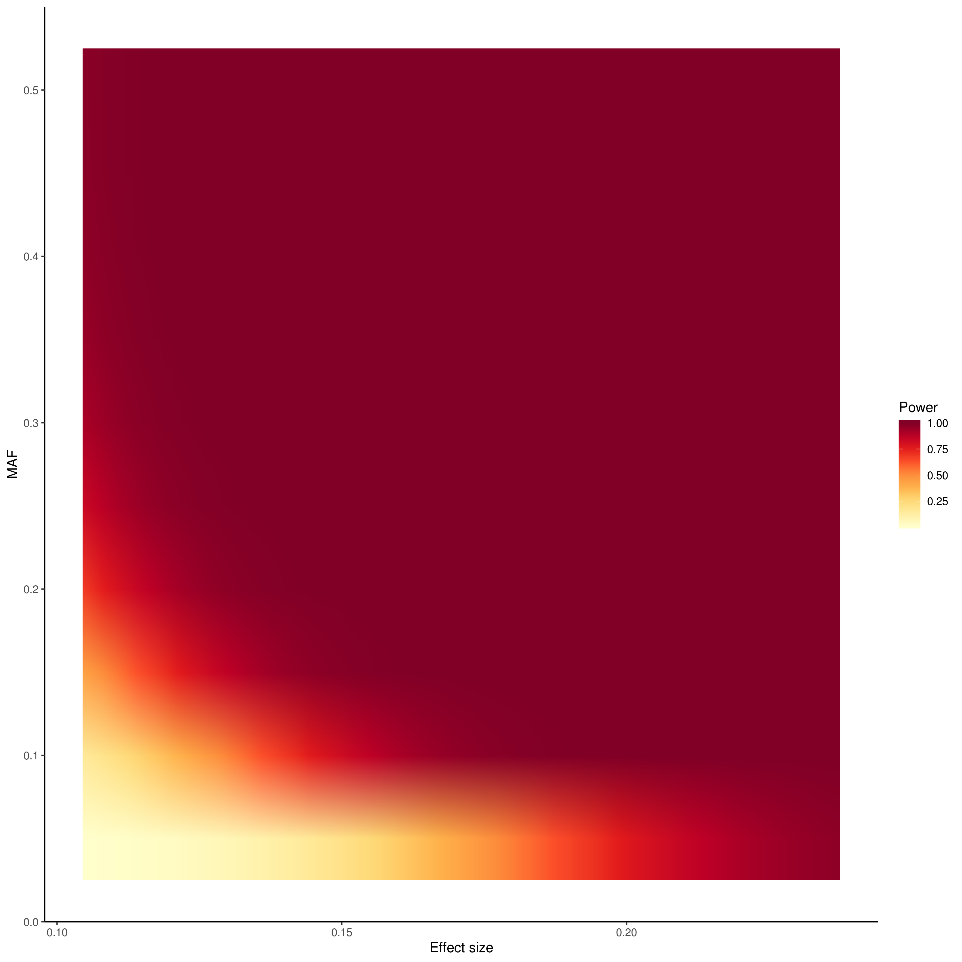 | 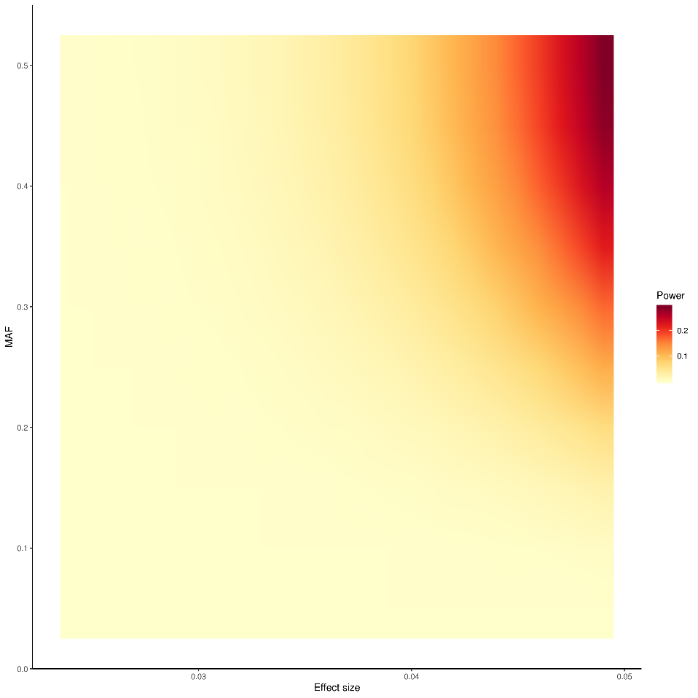 |
| HbA1c: Study variants at sample size 10K | HbA1c: Non-replicated variants at sample size 20K |
| 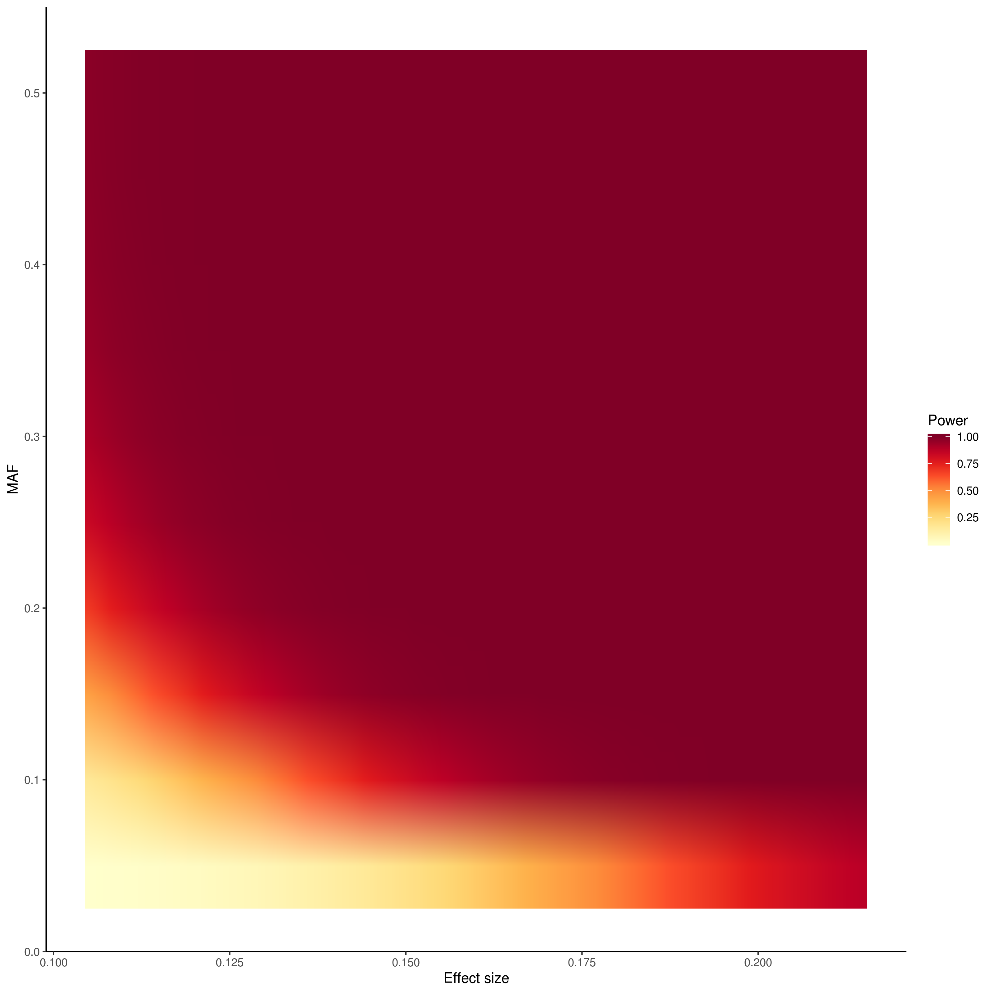 | 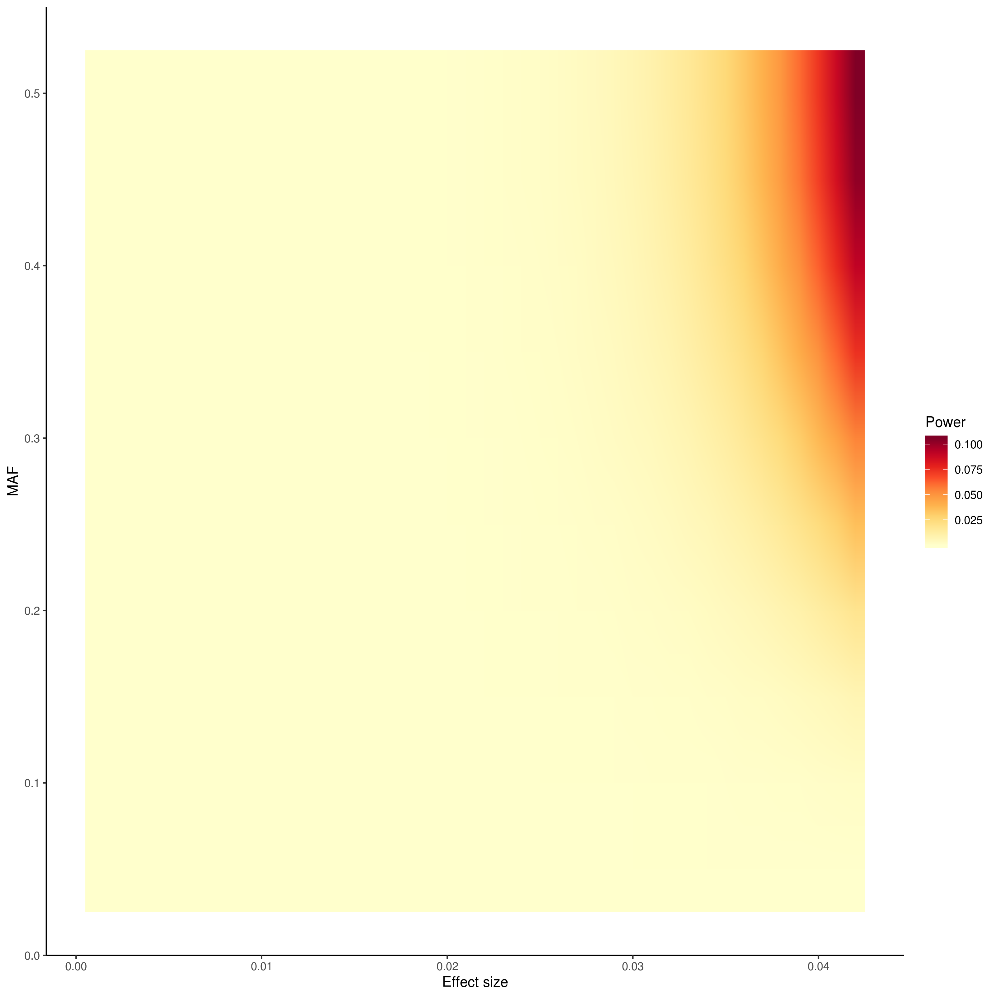 |
| HDL: Study variants at sample size 10K | HDL: Non-replicated variants at sample size 20K |
| 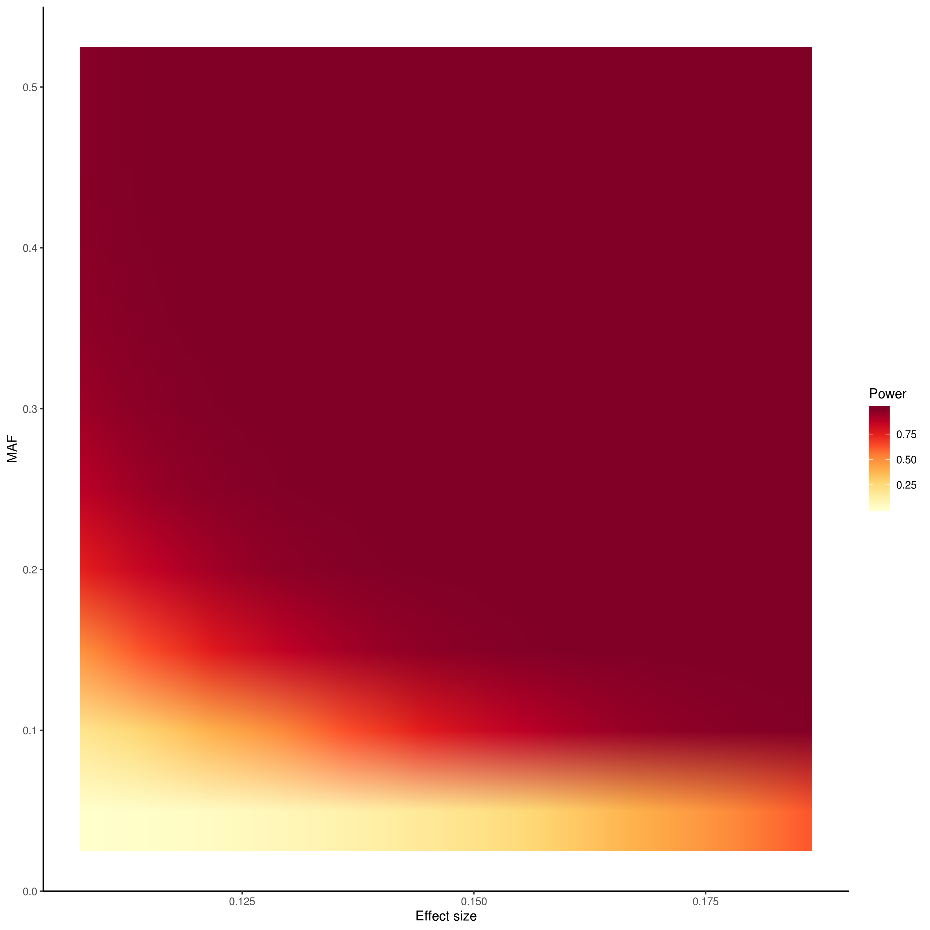 | 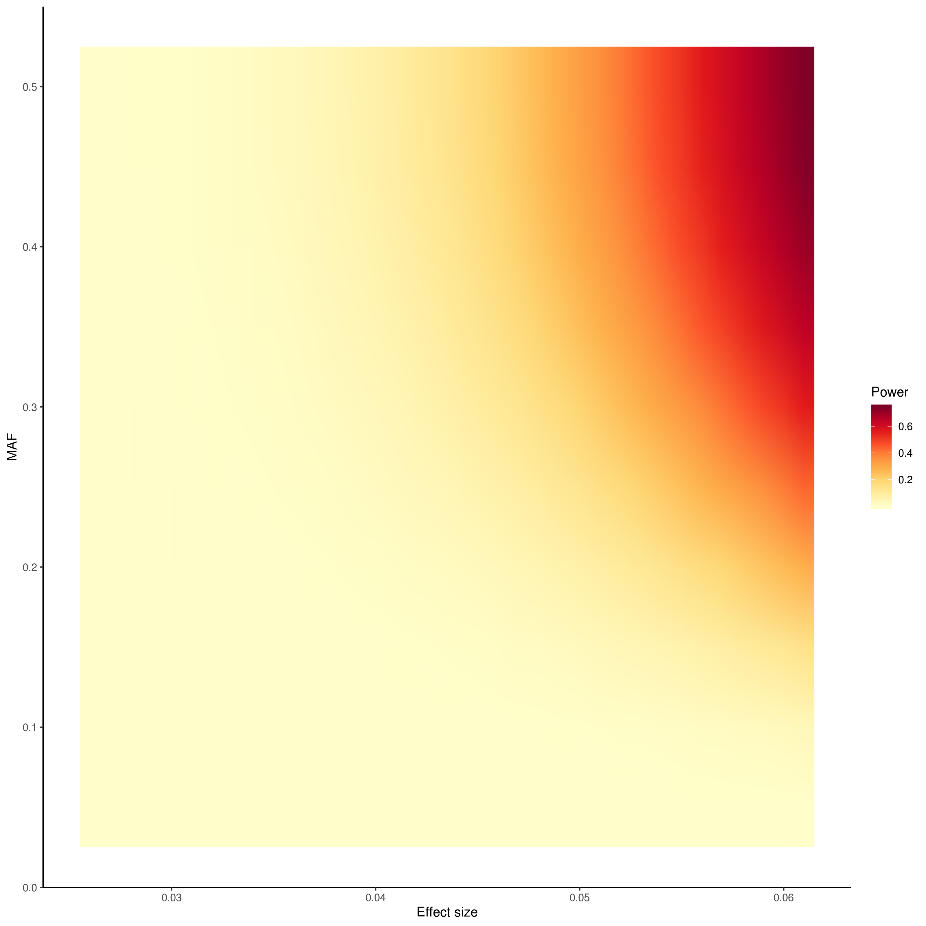 |
| Height: Study variants at sample size 10K | Height: Non-replicated variants at sample size 20K |
| 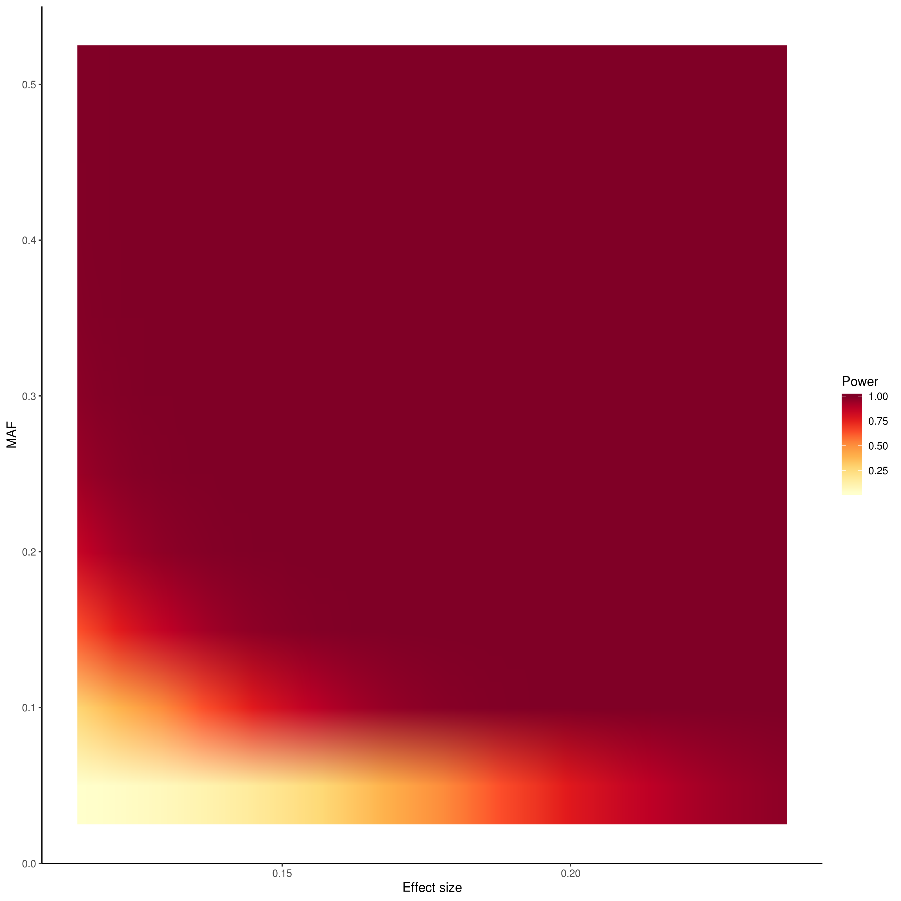 | 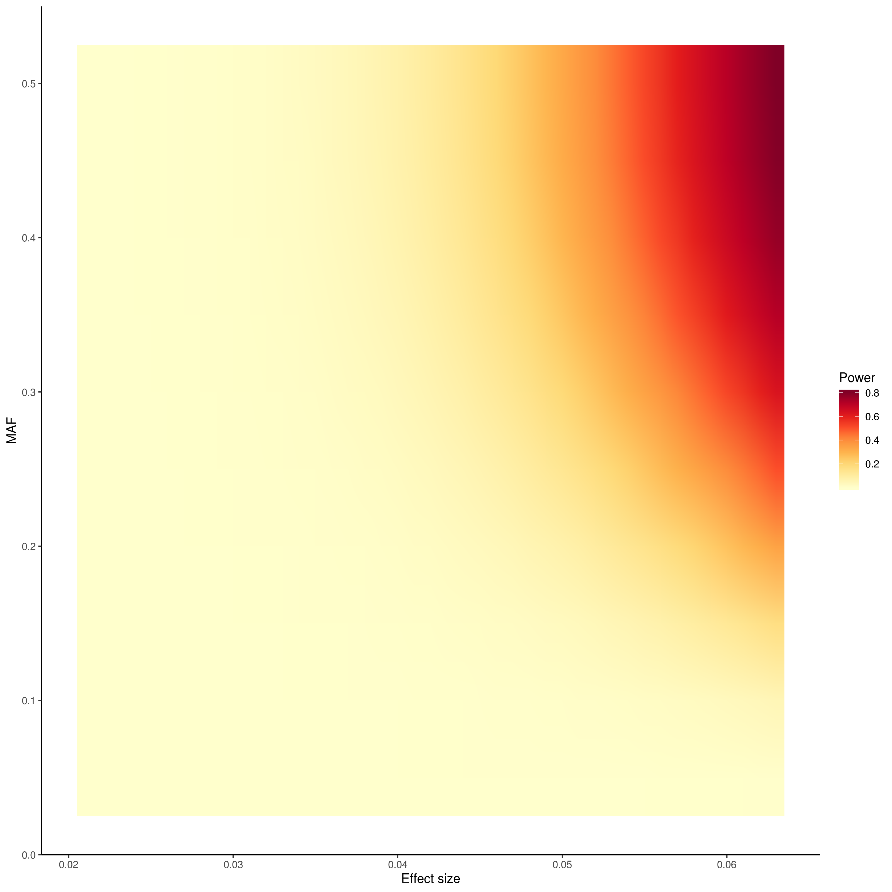 |
| LDL: Study variants at sample size 10K | LDL: Non-replicated variants at sample size 20K |
| 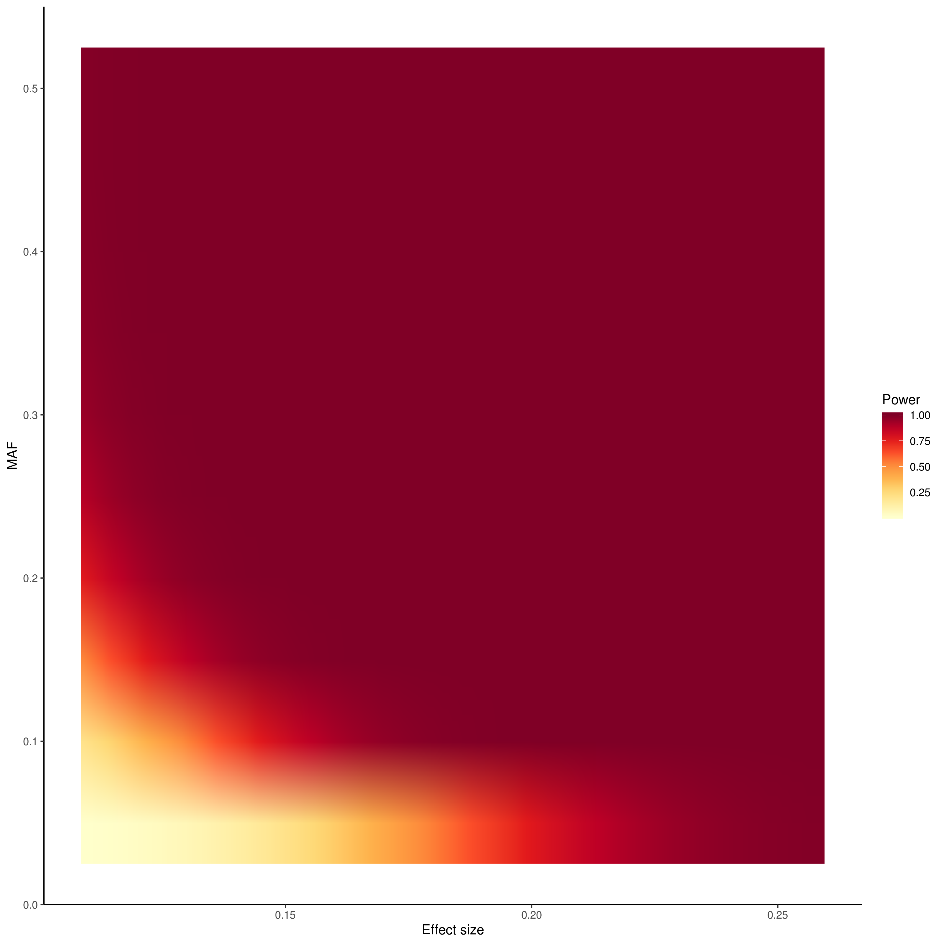 | 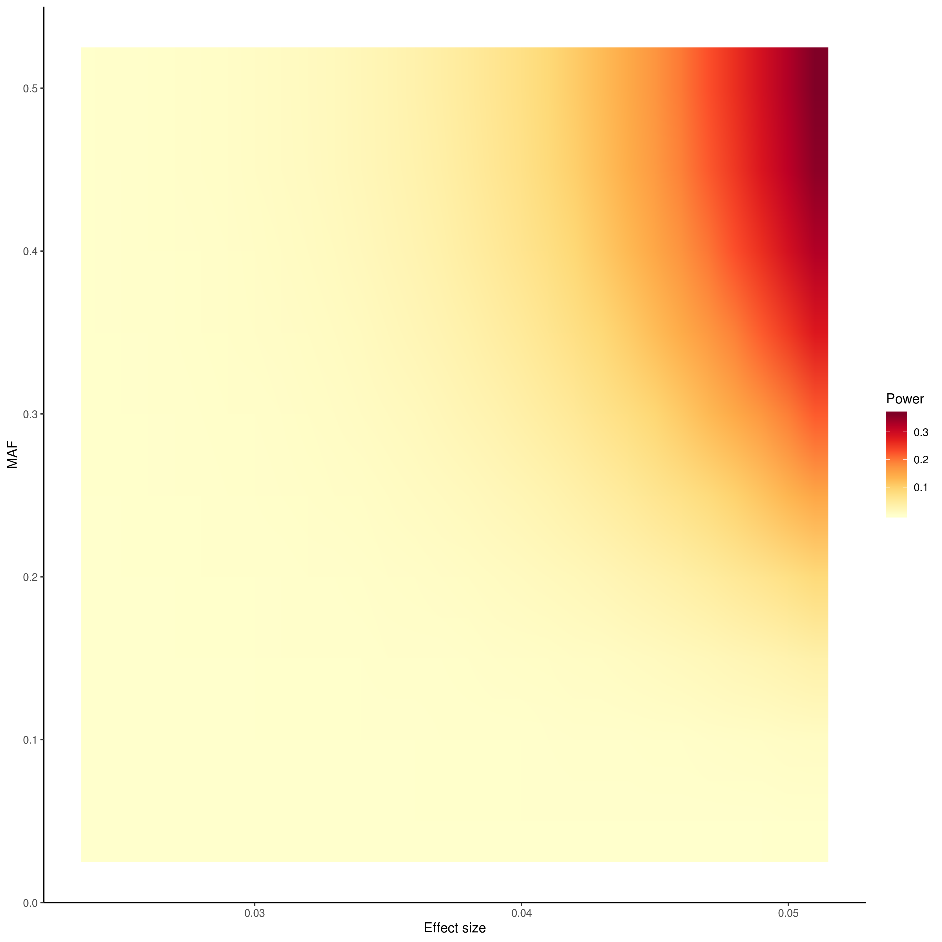 |
| SBP: Study variants at sample size 10K | SBP: Non-replicated variants at sample size 20K |
| 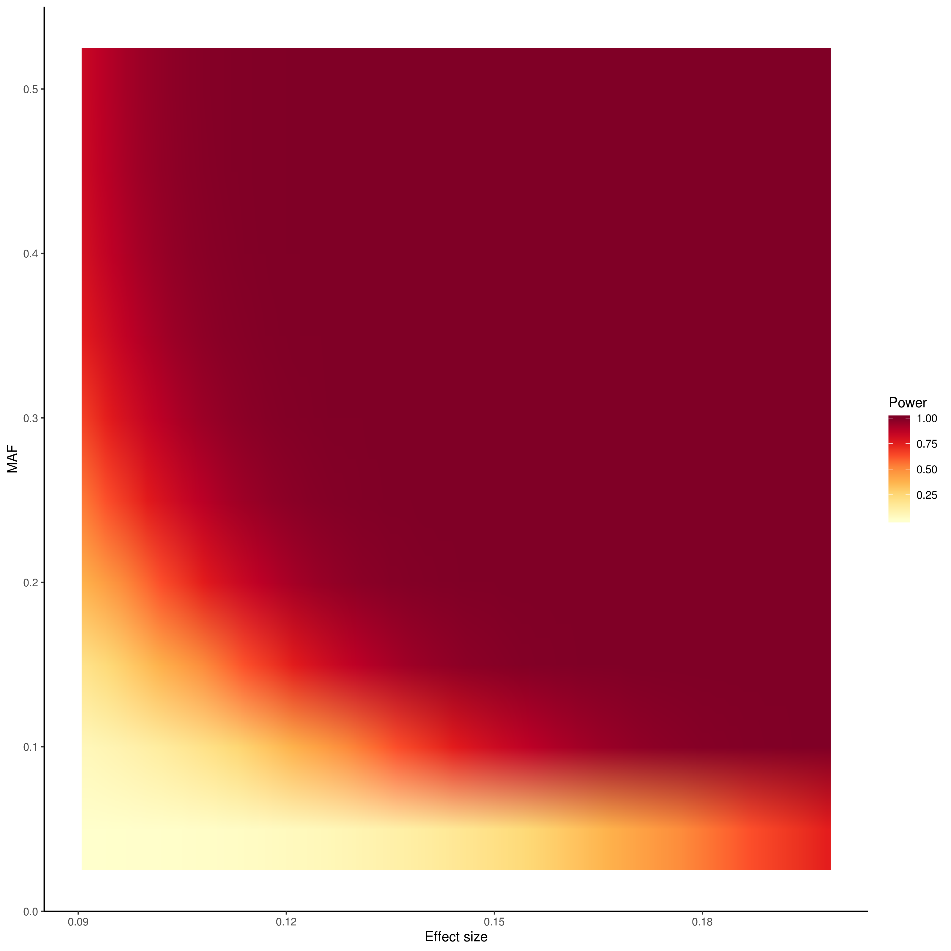 | 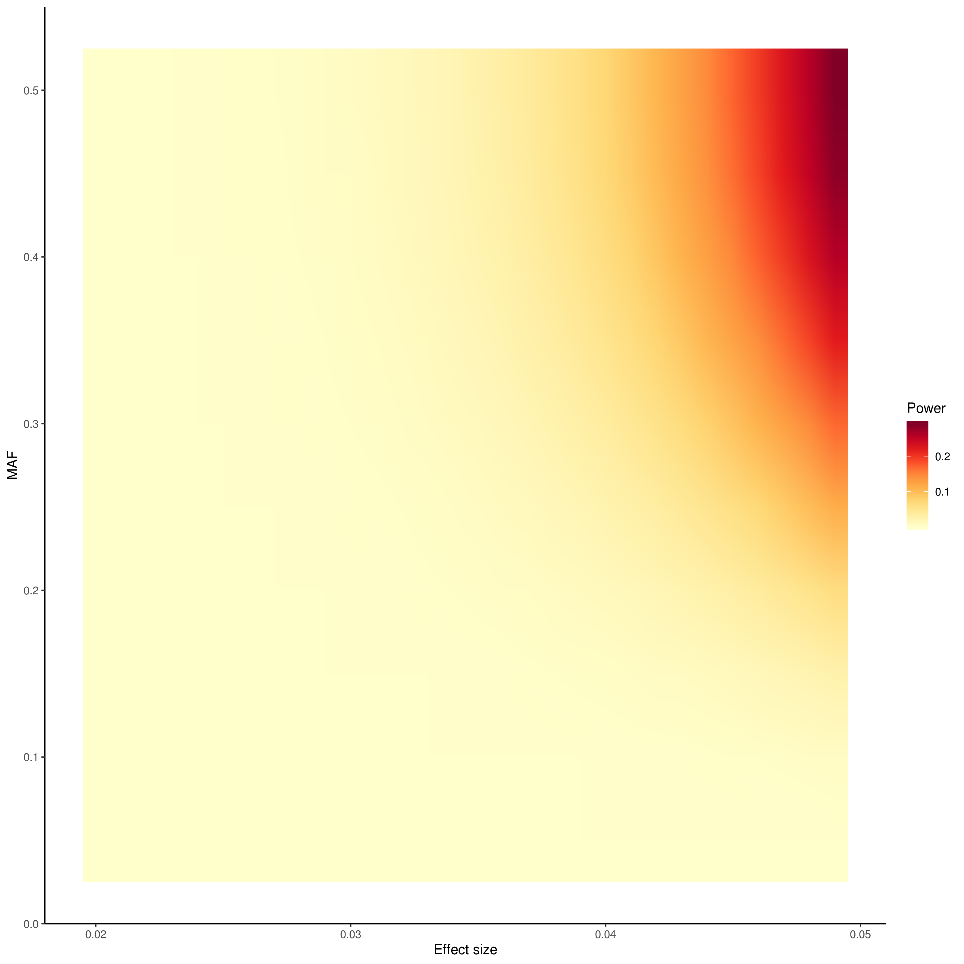 |
| TC: Study variants at sample size 10K | TC: Non-replicated variants at sample size 20K |
| 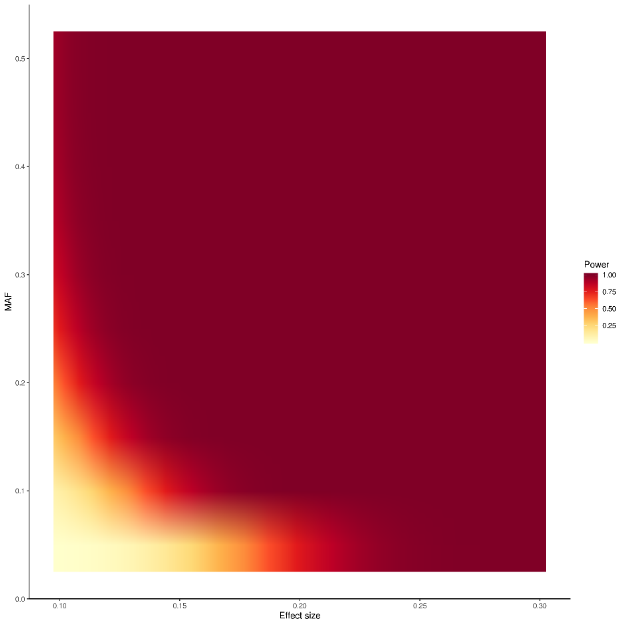 | 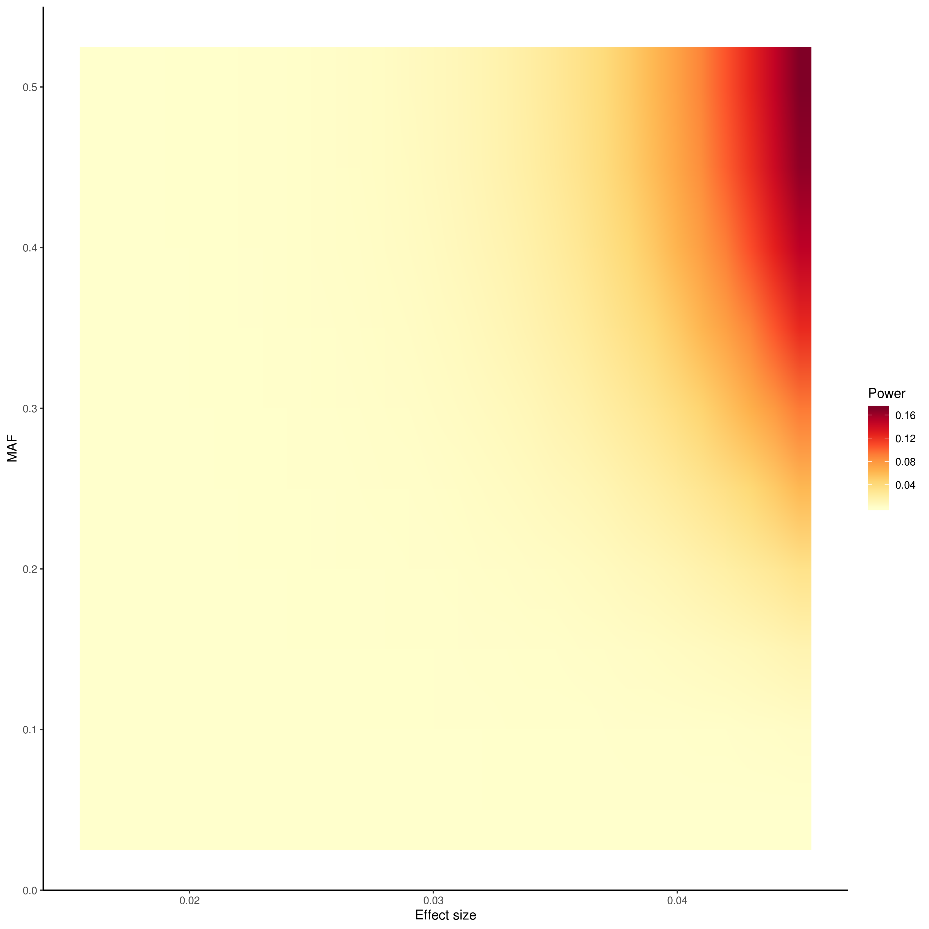 |
| TG: Study variants at sample size 10K | TG: Non-replicated variants at sample size 20K |
| 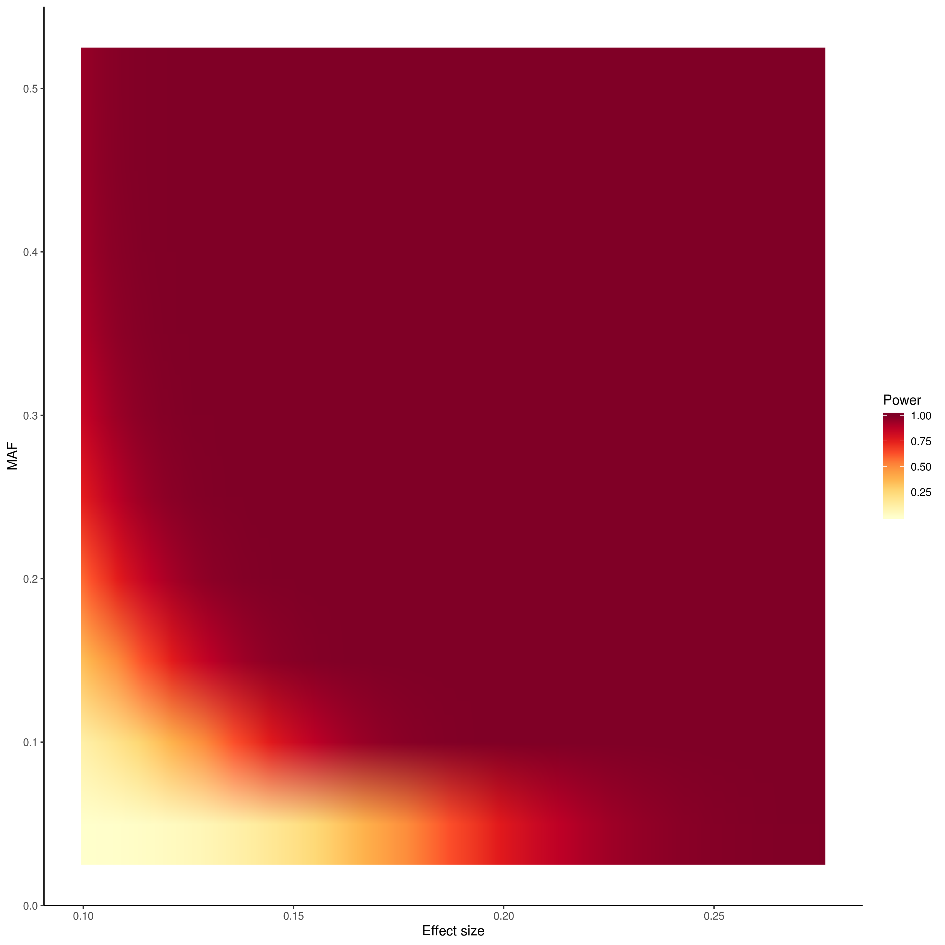 | 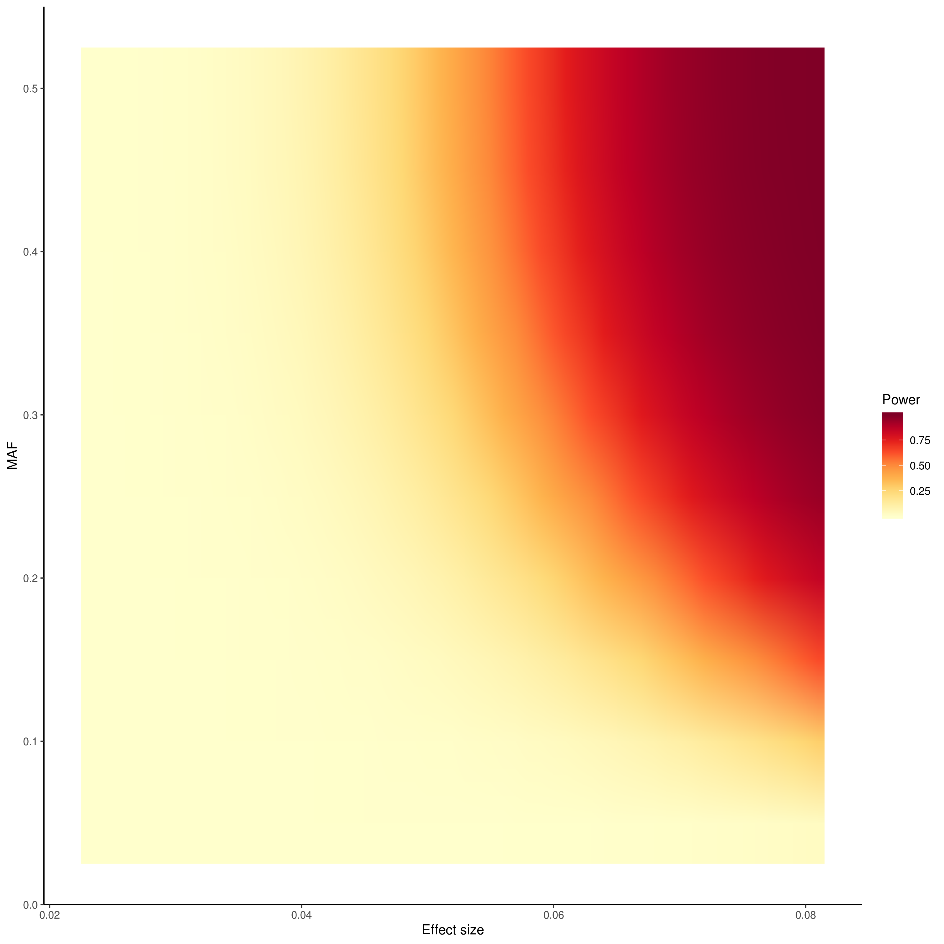 |
| WC: Study variants at sample size 10K | WC: Non-replicated variants at sample size 20K |
| 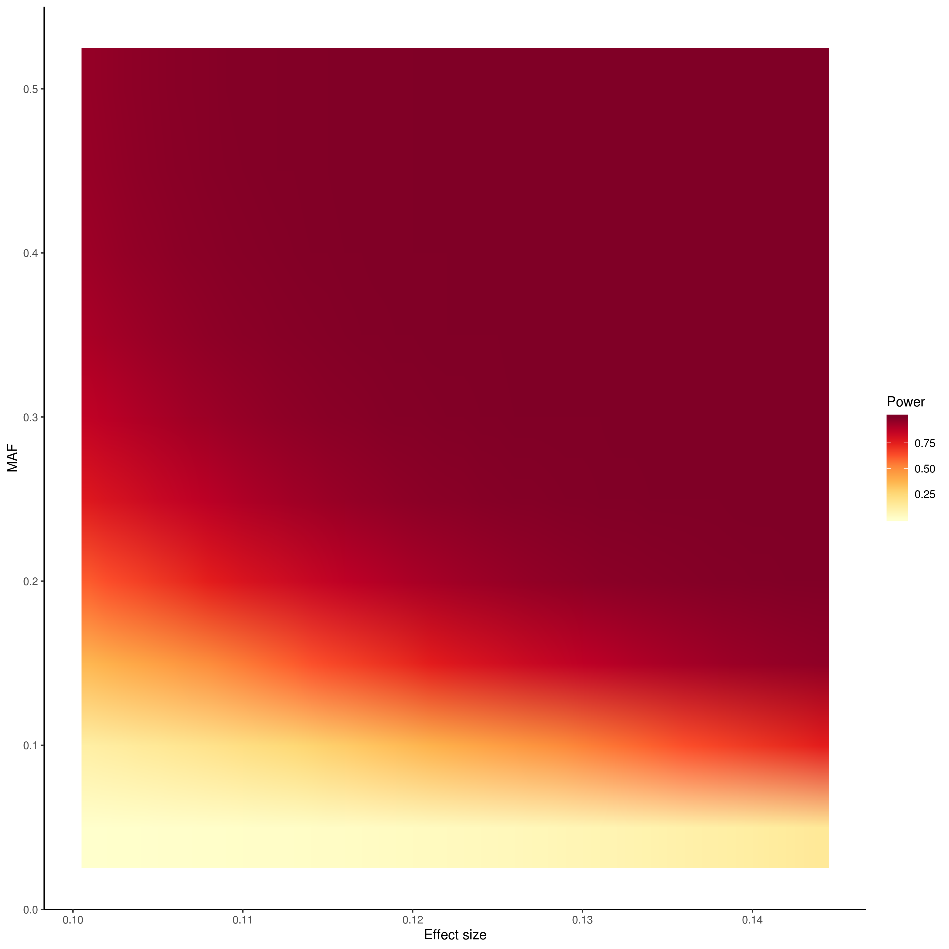 | 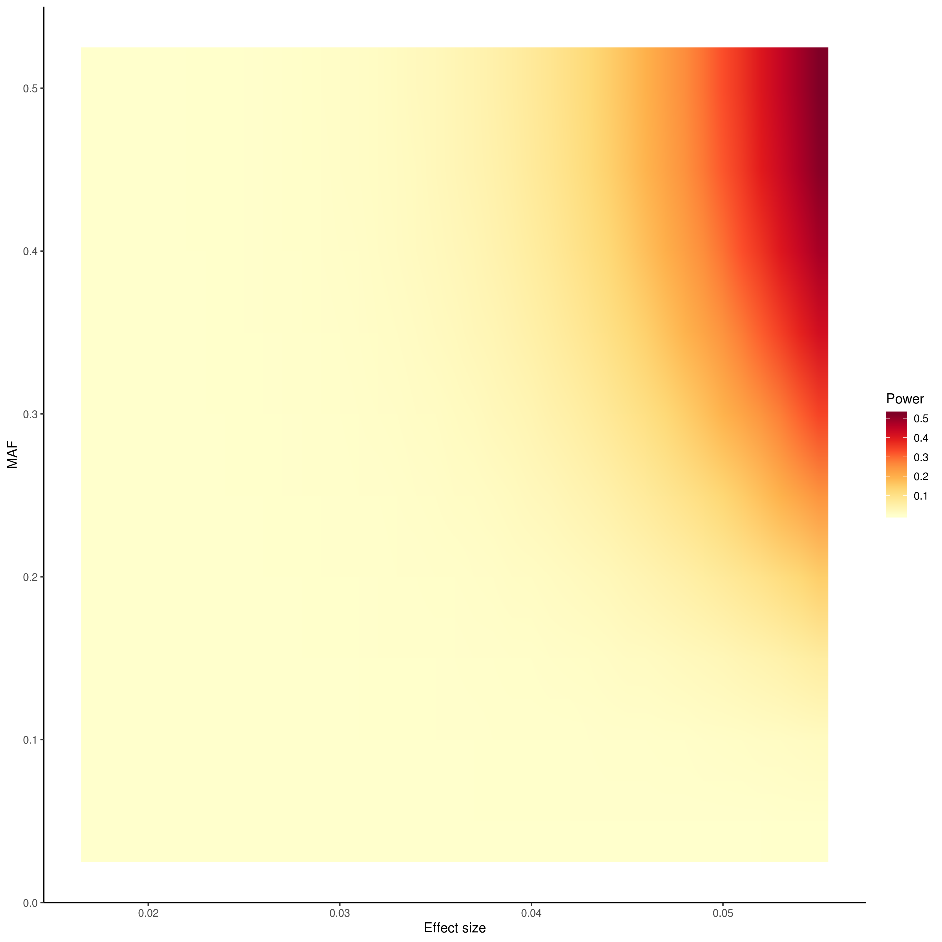 |
| Weight: Study variants at sample size 10K | Weight: Non-replicated variants at sample size 20K |

**Supplementary Figure S10: Functional consequences of the study variants in terms of functional consequence (A) and proximity to transcription start sites (B).**


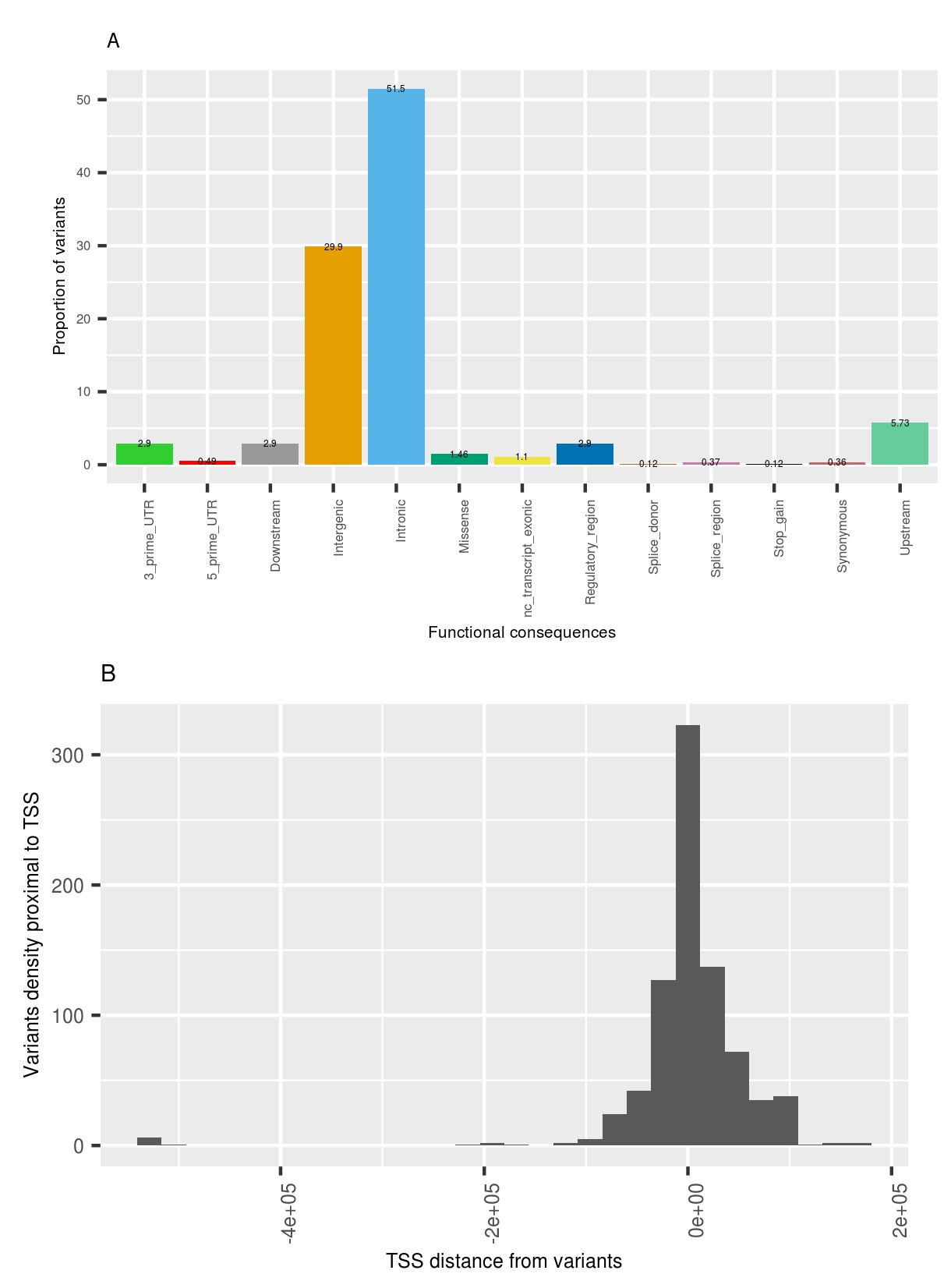


**Supplementary Figure S11. Tissue-wide distribution of gene regulatory patterns (as deduced by considering eQTL from GTEx) by the study variants (A) and at the level of genes harboring the variants (B).**


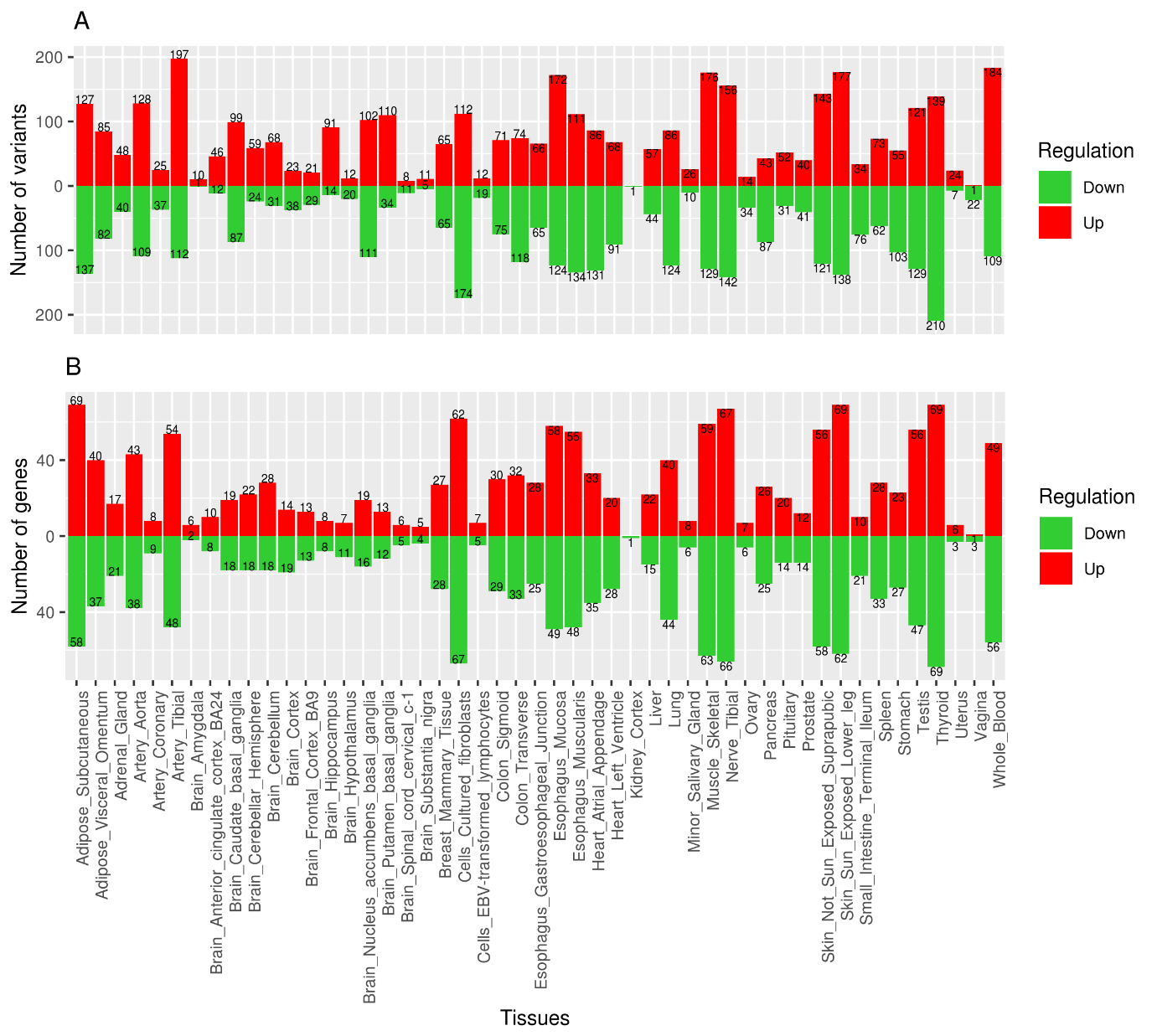


**Supplementary Figure S12. Tissue-wide distribution of gene regulatory patterns (as deduced by considering only those eQTL with Q-value ≤ 0.05 from GTEx) by the study variants (A) and at the level of genes harboring the variants (B).**


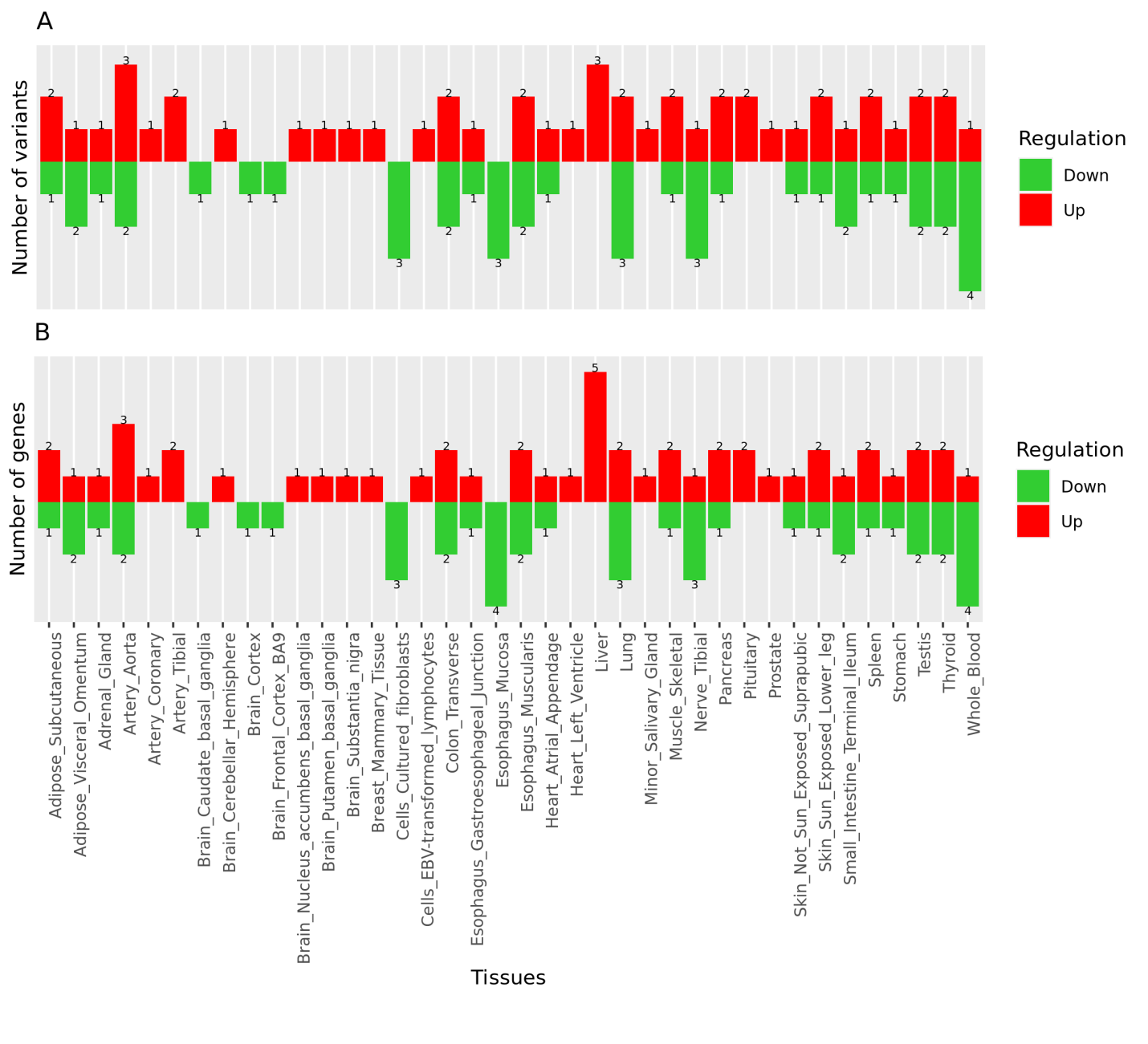

Supplement: Supplementary file 1 — Supplementary file1 (DOCX 13940 kb) [file 439_2020_2222_MOESM1_ESM.docx]
