# Supplementary material for: Genome-wide landscape establishes novel association signals for metabolic traits in the Arab population
Source: Hum Genet. 2020 Sep 9;140(3):505–28. doi: 10.1007/s00439-020-02222-7 (PMC7889551; doi:10.1007/s00439-020-02222-7)
Supplement: Supplementary file 2 — Supplementary file2 (DOCX 47 kb) [file 439_2020_2222_MOESM2_ESM.docx]

**Supplementary Table S1. Statistics on the number of imputed SNPs as per different R-squared (Rsq) threshold values.** The Rsq represents per-sample imputation confidence scores between true and imputed genotypes averaged over imputation chunks. The corresponding mean values for Rsq are listed for each set of SNPs satisfying the Rsq threshold.

| Genotyping array | Cut-off threshold for Rsq | No. of **imputed SNPs** | Mean (±SD) values of Rsq |
| --- | --- | --- | --- |
| HumanOmniExpress | Rsq≥0.0 (Full set of imputed markers) | 48424667 | 0.285 (±0.308) |
| HumanCardio-Metabo | Rsq≥0.0 (Full set of imputed markers) | 46597973 | 0.074 (±0.175) |
| HumanOmniExpress | Rsq≥0.3 | 18501610 | 0.633 (±0.212) |
| HumanCardio-Metabo | Rsq≥0.3 | 3657531 | 0.610 (±0.225) |
| HumanOmniExpress | Rsq≥0.4 | 15056208 | 0.696 (±0.182) |
| HumanCardio-Metabo | Rsq≥0.4 | 2782428 | 0.691 (±0.195) |
| HumanOmniExpress | Rsq≥0.5 | 12228271 | 0.754 (±0.152) |
| HumanCardio-Metabo | Rsq≥0.5 | 2163824 | 0.762 (±0.163) |
| HumanOmniExpress | Rsq≥0.5 & MAF ≥5% | 6083249 | 0.822 (±0.144) |
| HumanCardio-Metabo | Rsq≥0.5 & MAF ≥5% | 1521123 | 0.759 (±0.163) |

**Supplementary Table S2: The 313 search terms (labels) used in GWAS Catalog (version 1.0) to curate metabolic traits corresponding to the study-specific 13 traits.** The 313 terms fall into five classes of anthropometry & obesity, blood pressure & hypertension, glycemia and diabetes, lipid profile, and cardiometabolic phenotypes.

| **Trait Class (count of search terms)** | **Search terms (labels) used to curate metabolic traits in GWAS Catalog** |
| --- | --- |
| Anthropometry and obesity (99) | Obesity (extreme); Height; Obesity-related traits; Body mass index; Waist-hip ratio; Body mass in chronic obstructive pulmonary disease; Weight; Waist circumference; Waist circumference and related phenotypes; Obesity (early onset extreme); Body mass index (SNP x SNP interaction); Obesity; Body mass index in non-asthmatics; Anthropometric traits in newborns; Fat distribution (HIV); Weight loss (gastric bypass surgery); Body mass index in asthmatics; Body mass index (change over time); Hip circumference (psychosocial stress interaction); Birth length; Body mass (lean); Anthropometric traits; Obesity and osteoporosis; Fat body mass; Visceral fat; Visceral adipose tissue/subcutaneous adipose tissue ratio; Visceral adipose tissue adjusted for BMI; Birth weight; Body mass index (education interaction); Body fat percentage; Waist circumference adjusted for body mass index; Waist-to-hip ratio adjusted for body mass index; BMI (smoking interaction); Waist circumference adjusted for BMI (smoking interaction); Body fat mass; Visceral adipose tissue; Childhood body mass index; Obesity in adult survivors of childhood cancer exposed to cranial radiation; Obesity in adult survivors of childhood cancer not exposed to cranial radiation; Body mass index variance; Body mass index in physically active individuals; Body mass index in physically inactive individuals; Waist circumference adjusted for BMI in active individuals; Waist circumference adjusted for BMI in inactive individuals; Waist-to-hip ratio adjusted for BMI in active individuals; Waist-to-hip ratio adjusted for BMI in inactive individuals; Body mass index (physical activity interaction); Body mass index (joint analysis main effects and physical activity interaction); Waist circumference adjusted for BMI (joint analysis main effects and physical activity interaction); Waist circumference adjusted for BMI (adjusted for smoking behaviour); Waist circumference adjusted for BMI (joint analysis main effects and smoking interaction); Waist circumference adjusted for BMI in non-smokers; BMI (adjusted for smoking behaviour); Body mass index (joint analysis main effects and smoking interaction); BMI in smokers; Height adjusted BMI; Hip circumference adjusted for BMI; Waist-to-hip ratio adjusted for BMI (joint analysis for main effect and physical activity interaction); BMI in non-smokers; Waist-to-hip ratio adjusted for BMI (adjusted for smoking behaviour); Waist-to-hip ratio adjusted for BMI (smoking interaction); Waist-to-hip ratio adjusted for BMI in non-smokers; Body mass index (age interaction); Body mass index (recreational physical activity interaction); Body mass index (alcohol intake interaction); Waist-to-hip circumference ratio (alcohol intake interaction); Waist-to-hip circumference ratio (dietary energy interaction); Waist-to-hip circumference ratio (recreational physical activity interaction); Body mass index (smoking years interaction); Waist-to-hip circumference ratio (smoking years interaction); Waist-to-hip circumference ratio (ever vs never smoking interaction); Body mass index (ever vs never smoking interaction); Body mass index (dietary energy interaction); Waist circumference adjusted for BMI in smokers; Waist-to-hip ratio adjusted for BMI in smokers; Waist-to-hip ratio adjusted for BMI (joint analysis main effects and smoking interaction); Body mass index (adult); Visceral adipose tissue/subcutaneous adipose tissue ratio adjusted for BMI; Body mass index (change over time) in cancer; Body mass index (change over time) in lung cancer; Body mass index (change over time) in gastrointestinal cancer; Body mass index (change over time) in chronic obstructive pulmonary disease; Body mass index (change over time) in gastrointestinal cancer or chronic obstructive pulmonary disease; Body mass index (change over time) in lung cancer or chronic obstructive pulmonary disease; Body mass index (change over time) in cancer or chronic obstructive pulmonary disease; Visceral adipose tissue attenuation (Hounslow unit scale); Hip circumference; Body mass index (age <50); Body mass index (age>50); Body mass index x sex x age interaction (4df test); Body mass index x age interaction; Waist-to-hip ratio adjusted for BMI x sex interaction; Waist-to-hip ratio adjusted for BMI (age <50); Waist-to-hip ratio adjusted for BMI (age >50); Waist-to-hip ratio adjusted for BMI; Waist-to-hip ratio adjusted for BMI x sex x age interaction (4df test); Body mass index and waist-hip ratio (pleiotropy); Body mass index and cholesterol (psychopharmacological treatment); Waist Circumference - Triglycerides (WC-TG). |
| Blood pressure and hypertension (46) | Blood pressure; Diastolic blood pressure; Hypertension; Systolic blood pressure; Hypertension risk in short sleep duration; Blood pressure (anthropometric measures interaction); Hypertension (young onset); Plasma plasminogen activator levels; Systolic blood pressure (alcohol consumption interaction); Blood pressure measurement (cold pressor test); Blood pressure measurement (high sodium and potassium intervention); Blood pressure measurement (low sodium intervention); Blood pressure measurement (high sodium intervention); Diastolic blood pressure (alcohol consumption interaction); Mean arterial pressure (alcohol consumption interaction); Systolic blood pressure (long-term average); Diastolic blood pressure (long-term average); Mean arterial pressure (long-term average); Diastolic blood pressure response to hydrochlorothiazide in hypertension; Systolic blood pressure change trajectories; Systolic blood pressure (cigarette smoking interaction); Mean arterial pressure; Blood pressure (age interaction); Blood pressure (smoking interaction); Plasma plasminogen levels; Systolic blood pressure (dietary potassium intake interaction); Hypertension (SNP x SNP interaction); Blood pressure traits (multi-trait analysis); Systolic blood pressure in combination therapy (beta blocker and thiazide diuretic); Diastolic blood pressure in combination therapy (beta blocker and thiazide diuretic); Diastolic blood pressure x sodium interaction (2df test); Diastolic blood pressure x sodium interaction (1df test); Mean arterial pressure x sodium interaction (2df test); Systolic blood pressure x alcohol consumption interaction (2df test); Diastolic blood pressure x alcohol consumption interaction (2df test); Diastolic blood pressure x alcohol consumption (light vs heavy) interaction (2df test); Mean arterial pressure x alcohol consumption interaction (2df test); Diastolic blood pressure x smoking status (current vs non-current) interaction (2df test); Diastolic blood pressure x smoking status (current vs non-current) interaction (1df test); Systolic blood pressure x smoking status (ever vs never) interaction (2df test); Diastolic blood pressure x smoking status (ever vs never) interaction (1df test); Diastolic blood pressure x smoking status (ever vs never) interaction (2df test); Mean arterial pressure x alcohol consumption (light vs heavy) interaction (2df test); Diastolic blood pressure (cigarette smoking interaction); Systolic blood pressure night-to-day ratio in hypertension; Diastolic blood pressure night-to-day ratio in hypertension |
| Glycemia and diabetes (75) | Type 2 diabetes; Insulin resistance/response; Proinsulin levels; Type 1 diabetes; Glycated hemoglobin levels; Type 2 diabetes nephropathy; Response to metformin in type 2 diabetes (glycemic); Fasting plasma glucose; Insulin-like growth factors; Diabetes related insulin traits; Diabetes (incident); Diabetes (gestational); Fasting plasma glucose (childhood); Diabetic retinopathy in type 2 diabetes; Type 2 diabetes and other traits; Diabetic retinopathy; Homeostasis model assessment of beta-cell function (dietary factor interaction); Homeostasis model assessment of insulin resistance (dietary factor interaction); Fasting insulin (dietary factor interaction); Glycemic traits (pregnancy); Insulin-related traits; Two-hour glucose challenge; Type 2 diabetes (young onset) and obesity; Type 1 diabetes nephropathy; Diabetes in response to antihypertensive drug treatment (treatment strategy interaction); Response to metformin (IC50); Peak insulin response; Insulin secretion rate; Insulin disposition index; Fasting blood glucose; Prevalent type 2 diabetes; Type 2 diabetes (age of onset); Glucose homeostasis traits; Type 1 diabetes and autoimmune thyroid diseases; Diabetic kidney disease; Type 2 diabetes (dietary heme iron intake interaction); Fasting blood glucose adjusted for BMI; Glycemic traits; Diabetic nephropathy in type 1 diabetes; Insulin levels in response to oral glucose tolerance test (120 minutes); Insulin levels in response to oral glucose tolerance test (30 minutes); Response to metformin in type 2 diabetes (HbA1c reduction); Corrected insulin response; Insulin sensitivity index; Insulin levels; Area under the curve of insulin levels; Insulin levels adjusted for BMI; Corrected insulin response adjusted for insulin sensitivity index; Insulinoma-associated antigen 2 autoantibody levels in type 1 diabetes; Insulin levels in response to oral glucose tolerance test (fasting); Fasting blood insulin; Homeostasis model assessment of insulin resistance; Homeostasis model assessment of beta-cell function; Fasting blood insulin (BMI interaction); Fasting blood glucose (BMI interaction); Change in HbA1c levels in response to metformin treatment in type 2 diabetes; Change in glucose in response to thiazide diuretic treatment in hypertension; Blood glucose levels; Diabetes mellitus; Type 1 diabetes in high risk HLA genotype individuals (time to event); Type 1 diabetes autoantibodies in high risk HLA genotype individuals (time to event); Elevated fasting plasma glucose; Hemoglobin A1c levels; Diabetic macular edema in type 2 diabetes; Proliferative diabetic retinopathy in type 2 diabetes; Diabetic kidney disease in type 2 diabetes; Early diabetic kidney disease in type 2 diabetes; Diabetic kidney disease in type 2 diabetes (ESRD vs. no ESRD); Early diabetic kidney disease in diabetes; Diabetic kidney disease in diabetes (ESRD vs. no ESRD); Blood sugar levels; Glycemic traits (multi-trait analysis); QT interval (sulfonylurea treatment interaction); JT interval (sulfonylurea treatment interaction); QRS interval (sulfonylurea treatment interaction) |
| Lipid profiles (55) | Triglycerides; HDL cholesterol; Lipid traits; LDL cholesterol; Cholesterol, total; Hypertriglyceridemia; HDL Cholesterol - Triglycerides (HDLC-TG); Triglycerides-Blood Pressure (TG-BP); Cholesterol; Cholesterol and Triglycerides; LDL (oxidized); Triglyceride levels; HDL cholesterol levels; LDL cholesterol levels; Triglyceride to HDL cholesterol ratio; Response to exercise (triglyceride level interaction); Lipoprotein (a) - cholesterol levels; Total cholesterol levels; High density lipoprotein cholesterol levels; LDL cholesterol to HDL cholesterol ratio; Triglyceride change in response to fenofibrate in statin-treated type 2 diabetes; LDL cholesterol change in response to fenofibrate in statin-treated type 2 diabetes; HDL cholesterol change in response to fenofibrate in statin-treated type 2 diabetes; Cholesterol efflux capacity; Total triglycerides levels; Free cholesterol levels; Total cholesterol levels in HDL; Total cholesterol levels in LDL; Serum total cholesterol levels; Triglyceride levels in very large VLDL; Triglyceride levels in large VLDL; Total cholesterol levels in medium VLDL; Cholesterol ester levels in medium VLDL; Free cholesterol levels in medium VLDL; Triglyceride levels in medium VLDL; Total cholesterol levels in small VLDL; Free cholesterol levels in small VLDL; Free cholesterol levels in IDL; Total cholesterol levels in large LDL; Free cholesterol levels in large LDL; Total cholesterol levels in medium LDL; Cholesterol ester levels in medium LDL; Total cholesterol levels in small LDL; Free cholesterol levels in very large HDL; Triglyceride levels in very large HDL; Total cholesterol levels in large HDL; Cholesterol ester levels in very large HDL; Free cholesterol levels in large HDL; Total cholesterol levels in medium HDL; Cholesterol ester levels in medium HDL; Free cholesterol levels in medium HDL; Triglyceride levels in small HDL; Triglyceride levels in small VLDL; Triglyceride levels in very small VLDL; Cholesterol ester levels in large LDL |
| Cardiometabolic phenotypes (38) | Adiponectin levels; Coronary heart disease; Cardiovascular disease risk factors; Bilirubin levels; Myocardial infarction; C-reactive protein; Coronary artery disease; C-reactive protein levels; Major CVD; Adiposity; Homocysteine levels; Myocardial infarction in coronary artery disease; Bilirubin levels in extreme obesity; Myocardial infarction (early onset); Cardiovascular heart disease in diabetics; C-reactive protein and white blood cell count; C-reactive protein levels or total cholesterol levels (pleiotropy); C-reactive protein levels or triglyceride levels (pleiotropy); C-reactive protein levels or LDL-cholesterol levels (pleiotropy); C-reactive protein levels or HDL-cholesterol levels (pleiotropy); circulating leptin levels; Adiponectin levels in pregnancy; Cardiovascular disease in hypertension (calcium channel blocker interaction); Myocardial infarction in hypertension (calcium channel blocker interaction); Cardiovascular disease in hypertension (ACE inhibitor interaction); circulating leptin levels adjusted for BMI; Adiponectin levels (BMI-adjusted); Myocardial infarction in darapladib-treated cardiovascular disease (time to event); Myocardial infarction in cardiovascular disease (time to event) (darapladib treatment interaction); Coronary heart disease (SNP X SNP interaction); C-reactive protein (red blood cell fatty acid level interaction); Coronary artery disease (myocardial infarction, percutaneous transluminal coronary angioplasty, coronary artery bypass grafting, angina or chromic ischemic heart disease); Cardiovascular risk factors (age interaction); Cardiovascular risk factors; Coronary atherosclerosis (increased number of diseased vessels) (traffic exposure interaction); Coronary artery disease in type 1 diabetes; C-peptide levels in type I diabetes; Coronary artery disease in diabetes |

**Supplementary Table S3: SNP quality information corresponding to the top 70 markers that showed mean imputation quality score >= 0.5 and passed quality control steps (in terms of consistency in allele frequencies between the two BeadChips, and HWE**<10^−6^**).** These 70 markers showed association in meta-analysis at genome-wide significance (p-value <5.0×10^-08^) among the Kuwaiti population.

| **Chromosome: Position** | **SNP** | **Reference / Alternate allele** | **Alternate Allele Frequency** | | **Genotype counts (Reference homozygous/ Heterozygous/ Alternate homozygous)** | | **HWE P-value** | | **Mean imputation quality score** | | **Genotype status^Source^** |
| --- | --- | --- | --- | --- | --- | --- | --- | --- | --- | --- | --- |
|  |  |  | OE | CM | OE | CM | OE | CM | OE | CM |  |
| 2:55341367 | rs2920844 | C/T | 0.86 | 0.847 | 30/281/935 | 39/318/1073 | 1.18E-01 | 1.04E-02 | 0.946 | 0.80 | Imputed^OE,CM^ |
| 2:55341952 | rs2968781 | T/G | 0.86 | 0.847 | 30/281/935 | 39/318/1073 | 1.18E-01 | 1.41E-02 | 0.943 | 0.806 | Imputed^OE,CM^ |
| 4:190208378 | rs11132637 | A/G | 0.22 | 0.23 | 844/393/61 | 869/476/89 | 7.24E-02 | 3.54E-02 | 0.69 | 0.76 | Imputed^OE,CM^ |
| 4:190206771 | rs35387314 | T/C | 0.23 | 0.24 | 834/399/65 | 866/476/92 | 5.25E-02 | 1.72E-02 | 0.68 | 0.83 | Imputed^OE,CM^ |
| 4:190202004 | rs6846011 | G/A | 0.23 | 0.24 | 833/401/64 | 871/474/89 | 7.80E-02 | 2.90E-02 | 0.69 | 0.78 | Imputed^OE,CM^ |
| 4:190201161 | rs34453299 | G/A | 0.22 | 0.23 | 841/397/60 | 873/476/85 | 1.29E-01 | 6.91E-02 | 0.70 | 0.72 | Imputed^OE,CM^ |
| 4:190198217 | rs561984720 | GA/G | 0.22 | 0.23 | 855/383/60 | 874/476/84 | 4.40E-02 | 8.09E-02 | 0.64 | 0.69 | Imputed^OE,CM^ |
| 4:190191046 | rs9790417 | G/A | 0.23 | 0.24 | 833/398/67 | 872/474/88 | 3.53E-02 | 3.47E-02 | 0.71 | 0.72 | Imputed^OE,CM^ |
| 4:190188001 | rs11942078 | G/T | 0.23 | 0.24 | 832/400/66 | 871/476/87 | 5.37E-02 | 4.98E-02 | 0.71 | 0.7 | Imputed^OE,CM^ |
| 4:190184168 | rs35668405 | C/A | 0.23 | 0.24 | 834/398/66 | 874/473/87 | 4.32E-02 | 3.45E-02 | 0.71 | 0.68 | Imputed^OE,CM^ |
| 4:190184043 | rs35602882 | A/G | 0.22 | 0.23 | 840/394/64 | 898/460/76 | 5.02E-02 | 9.83E-02 | 0.72 | 0.63 | Imputed^OE,CM^ |
| 4:190179626 | rs35266160 | T/C | 0.23 | 0.23 | 836/398/64 | 902/462/70 | 6.37E-02 | 2.66E-01 | 0.71 | 0.6 | Imputed^OE,CM^ |
| 4:190178544 | rs11721957 | G/A | 0.23 | 0.24 | 830/402/66 | 878/471/85 | 5.53E-02 | 4.76E-02 | 0.71 | 0.65 | Imputed^OE,CM^ |
| 4:190172499 | rs35466621 | G/A | 0.23 | 0.24 | 831/401/66 | 895/462/77 | 5.43E-02 | 9.97E-02 | 0.69 | 0.62 | Imputed^OE,CM^ |
| 4:190170843 | rs35699925 | TAA/T | 0.23 | 0.24 | 829/403/66 | 898/459/77 | 6.69E-02 | 7.10E-02 | 0.69 | 0.61 | Imputed^OE,CM^ |
| 4:190169159 | rs34329457 | T/C | 0.23 | 0.24 | 831/402/65 | 899/461/74 | 8.14E-02 | 1.55E-01 | 0.69 | 0.61 | Imputed^OE,CM^ |
| 4:190167174 | rs13147669 | G/A | 0.23 | 0.24 | 832/401/65 | 902/462/70 | 8.03E-02 | 2.66E-01 | 0.68 | 0.6 | Imputed^OE,CM^ |
| 4:190161531 | rs76018028 | T/C | 0.23 | 0.24 | 834/398/66 | 907/458/69 | 4.39E-02 | 2.61E-01 | 0.67 | 0.58 | Imputed^OE,CM^ |
| 4:190160826 | rs35192598 | C/T | 0.22 | 0.22 | 847/388/63 | 912/463/59 | 3.92E-02 | 1.00E+00 | 0.67 | 0.54 | Imputed^OE,CM^ |
| 4:190159637 | rs55944922 | G/T | 0.23 | 0.24 | 835/397/66 | 908/457/69 | 4.32E-02 | 2.60E-01 | 0.67 | 0.57 | Imputed^OE,CM^ |
| 4:190159385 | rs13141596 | G/A | 0.22 | 0.22 | 849/386/63 | 912/464/58 | 3.81E-02 | 1.00E+00 | 0.67 | 0.53 | Imputed^OE,CM^ |
| 4:190157839 | rs13120111 | G/A | 0.23 | 0.24 | 846/387/65 | 910/458/66 | 2.00E-02 | 3.75E-01 | 0.66 | 0.56 | Imputed^OE,CM^ |
| 4:190157713 | rs13119288 | A/G | 0.23 | 0.24 | 845/387/66 | 910/458/66 | 1.59E-02 | 3.75E-01 | 0.66 | 0.56 | Imputed^OE,CM^ |
| 4:190157576 | rs36021514 | G/A | 0.23 | 0.24 | 846/387/65 | 910/459/65 | 2.00E-02 | 4.67E-01 | 0.66 | 0.56 | Imputed^OE,CM^ |
| 4:190157486 | rs34728227 | G/A | 0.23 | 0.24 | 847/386/65 | 910/462/62 | 1.96E-02 | 7.45E-01 | 0.66 | 0.56 | Imputed^OE,CM^ |
| 4:190157056 | rs35127507 | G/A | 0.23 | 0.24 | 847/386/65 | 910/463/61 | 1.96E-02 | 8.08E-01 | 0.66 | 0.55 | Imputed^OE,CM^ |
| 4:190156646 | rs34706863 | G/T | 0.23 | 0.24 | 850/383/65 | 910/468/56 | 1.49E-02 | 7.43E-01 | 0.66 | 0.55 | Imputed^OE,CM^ |
| 8:4126701 | rs7838666 | T/C | 0.689 | 0.685 | 108/449/720 | 141/564/729 | 2.23E-03 | 4.2E-02 | 0.547 | 0.510 | Imputed^OE,CM^ |
| 8:4128311 |  | C/T | 0.306 | 0.282 | 717/454/106 | 825/505/104 | 5.79E-03 | 3.37E-02 | 0.548 | 0.516 | Imputed^OE,CM^ |
| 8:19745039 | rs10635970 | T/TAA | 0.5 | 0.51 | 326/607/365 | 349/680/386 | 9.89E-03 | 1.51E-01 | 0.87 | 0.92 | Imputed^OE,CM^ |
| 8:19750951 | rs112861901 | T/C | 0.11 | 0.11 | 1060/229/9 | 1142/280/12 | 6.20E-01 | 3.28E-01 | 0.81 | 0.99 | Imputed^OE,CM^ |
| 11:116623213 | rs66505542 | TA/T | 0.75 | 0.72 | 96/438/764 | 117/580/737 | 1.95E-02 | 8.46E-01 | 0.88 | 1 | Imputed^OE,CM^ |
| 16:57006829 | rs289713 | T/A | 0.78 | 0.78 | 70/389/839 | 74/504/856 | 1.31E-02 | 1.00E+00 | 0.83 | 0.96 | Imputed^OE,CM^ |
| 16:57006590 | rs7499892 | C/T | 0.19 | 0.2 | 928/322/48 | 942/439/53 | 7.76E-03 | 8.64E-01 | 0.81 | 1 | Imputed^OE,^Genotyped^CM^ |
| 16:57006378 | rs11076175 | A/G | 0.17 | 0.18 | 939/316/43 | 968/422/44 | 2.73E-02 | 8.57E-01 | 0.87 | 0.99 | Imputed^OE,CM^ |
| 16:57001274 | rs200751500 | AC/A | 0.29 | 0.28 | 709/476/113 | 764/548/122 | 9.27E-03 | 9.86E-02 | 0.69 | 0.99 | Imputed^OE,CM^ |
| 16:57001254 | rs12720908 | T/TCACA | 0.19 | 0.2 | 869/375/54 | 923/462/49 | 1.83E-01 | 4.00E-01 | 0.93 | 1 | Imputed^OE,CM^ |
| 16:57000885 | rs12720922 | G/A | 0.19 | 0.2 | 870/374/54 | 923/461/50 | 1.54E-01 | 4.51E-01 | 0.92 | 1 | Imputed^OE,^ Genotyped^CM^ |
| 16:56999778 | rs17231569 | CG/C | 0.19 | 0.2 | 869/375/54 | 920/459/55 | 1.83E-01 | 8.68E-01 | 0.91 | 1 | Imputed^OE,CM^ |
| 16:56999328 | rs11508026 | C/T | 0.39 | 0.39 | 507/564/227 | 568/645/221 | 1.86E-03 | 9.25E-02 | 0.72 | 1 | Imputed^OE,CM^ |
| 16:56999258 | rs7203984 | A/C | 0.24 | 0.23 | 819/405/74 | 858/503/73 | 2.65E-02 | 1.00E+00 | 0.80 | 1 | Imputed^OE,^ Genotyped^CM^ |
| 16:56998918 | rs12720926 | A/G | 0.39 | 0.38 | 506/564/228 | 577/638/219 | 1.86E-03 | 5.55E-02 | 0.72 | 0.99 | Imputed^OE,CM^ |
| 16:56997349 | rs5817082 | C/CA | 0.29 | 0.32 | 744/456/98 | 673/608/153 | 2.67E-02 | 3.62E-01 | 0.73 | 1 | Imputed^OE,CM^ |
| 16:56997233 | rs1864163 | G/A | 0.25 | 0.29 | 794/422/82 | 743/564/127 | 2.14E-02 | 1.95E-01 | 0.76 | 1 | Imputed^OE,^ Genotyped^CM^ |
| 16:56996649 | rs34620476 | C/A | 0.41 | 0.39 | 466/576/256 | 558/654/222 | 6.69E-04 | 1.99E-01 | 0.76 | 1 | Imputed^OE,^ Genotyped^CM^ |
| 16:56996645 | rs34145065 | GCC/G | 0.41 | 0.38 | 465/578/255 | 569/647/218 | 1.02E-03 | 1.29E-01 | 0.76 | 0.99 | Imputed^OE,CM^ |
| 16:56996288 | rs708272 | G/A | 0.42 | 0.4 | 452/567/279 | 542/662/230 | 2.80E-05 | 2.45E-01 | 0.77 | 1 | Imputed^OE,CM^ |
| 16:56996211 | rs711752 | G/A | 0.42 | 0.4 | 451/568/279 | 542/662/230 | 3.61E-05 | 2.45E-01 | 0.77 | 1 | Imputed^OE,^ Genotyped^CM^ |
| 16:56996158 | rs3816117 | T/C | 0.52 | 0.52 | 315/618/365 | 340/724/370 | 5.95E-02 | 7.12E-01 | 0.97 | 1 | Imputed^OE,^ Genotyped^CM^ |
| 16:56995236 | rs1800775 | C/A | 0.52 | 0.51 | 314/619/365 | 344/717/373 | 6.77E-02 | 1.00E+00 | 1.00 | 1 | Genotyped^OE,CM^ |
| 16:56994528 | rs17231506 | C/T | 0.3 | 0.29 | 654/510/134 | 741/579/114 | 7.79E-03 | 9.48E-01 | 0.77 | 1 | Imputed^OE,^ Genotyped^CM^ |
| 16:56994244 | rs36229491 | T/TA | 0.3 | 0.29 | 656/508/134 | 741/579/114 | 6.25E-03 | 9.48E-01 | 0.77 | 1 | Imputed^OE,CM^ |
| 16:56993886 | rs821840 | A/G | 0.32 | 0.3 | 603/536/159 | 722/588/124 | 9.39E-03 | 7.98E-01 | 0.78 | 1 | Imputed^OE,CM^ |
| 16:56993324 | rs3764261 | C/A | 0.33 | 0.31 | 598/536/164 | 698/600/136 | 5.51E-03 | 6.63E-01 | 0.76 | 1 | Imputed^OE,^ Genotyped^CM^ |
| 16:56993161 | rs12149545 | G/A | 0.28 | 0.27 | 694/481/123 | 768/560/106 | 2.27E-03 | 7.88E-01 | 0.74 | 1 | Imputed^OE,^ Genotyped^CM^ |
| 16:56991948 | rs201825234 | G/A | 0.17 | 0.2 | 997/269/32 | 952/412/70 | 1.67E-02 | 4.97E-03 | 0.62 | 0.98 | Imputed^OE,CM^ |
| 16:56991524 | rs6499862 | G/A | 0.18 | 0.21 | 986/273/39 | 920/436/78 | 1.08E-03 | 7.68E-03 | 0.61 | 1 | Imputed^OE,^ Genotyped^CM^ |
| 16:56991495 | rs6499861 | C/G | 0.18 | 0.2 | 995/270/33 | 948/417/69 | 1.23E-02 | 1.10E-02 | 0.62 | 1 | Imputed^OE,CM^ |
| 16:56991363 | rs183130 | C/T | 0.32 | 0.29 | 614/532/152 | 729/584/121 | 8.64E-03 | 7.97E-01 | 0.74 | 1 | Imputed^OE,^ Genotyped^CM^ |
| 16:56990716 | rs247617 | C/A | 0.32 | 0.29 | 617/529/152 | 730/584/120 | 5.83E-03 | 8.47E-01 | 0.73 | 1 | Imputed^OE,^ Genotyped^CM^ |
| 16:56989590 | rs247616 | C/T | 0.32 | 0.29 | 618/530/150 | 730/584/120 | 8.53E-03 | 8.47E-01 | 0.73 | 1 | Imputed^OE,CM^ |
| 16:56988502 | rs2399597 | G/A | 0.18 | 0.2 | 1006/260/32 | 950/415/69 | 6.90E-03 | 8.54E-03 | 0.60 | 0.99 | Imputed^OE,CM^ |
| 16:56988044 | rs173539 | C/T | 0.34 | 0.32 | 592/535/171 | 661/630/143 | 1.37E-03 | 7.16E-01 | 0.68 | 1 | Imputed^OE,^ Genotyped^CM^ |
| 16:56987765 | rs56228609 | C/T | 0.28 | 0.27 | 710/474/114 | 772/557/105 | 4.62E-03 | 7.36E-01 | 0.68 | 1 | Imputed^OE,CM^ |
| 16:56987369 | rs56156922 | T/C | 0.3 | 0.29 | 677/497/124 | 741/579/114 | 9.79E-03 | 9.48E-01 | 0.68 | 1 | Imputed^OE,^ Genotyped^CM^ |
| 16:56987015 | rs12446515 | C/T | 0.3 | 0.28 | 680/494/124 | 754/570/110 | 7.69E-03 | 8.95E-01 | 0.68 | 1 | Imputed^OE,^ Genotyped^CM^ |
| 16:56986914 | rs7205692 | A/G | 0.19 | 0.2 | 984/278/36 | 939/424/71 | 7.82E-03 | 1.24E-02 | 0.58 | 1 | Imputed^OE,^ Genotyped^CM^ |
| 16:56986762 | rs7203286 | G/T | 0.47 | 0.5 | 439/590/269 | 395/672/367 | 8.99E-03 | 2.00E-02 | 0.54 | 0.93 | Imputed^OE,CM^ |
| 16:56985805 | rs11862052 | C/T | 0.18 | 0.19 | 1016/254/28 | 987/384/63 | 1.59E-02 | 2.04E-03 | 0.55 | 0.9 | Imputed^OE,CM^ |
| 21:38906071 | rs2835788 | C/G | 0.12 | 0.12 | 1038/195/13 | 1189/218/23 | 2.80E-01 | 1.42E-03 | 0.638 | 0.731 | Imputed^OE,CM^ |
|  |  |  |  |  |  |  |  |  |  |  |  |

**Supplementary Table S4: The 72 associations observed at genome-wide significance involving the top 70 markers (as listed in Supplementary Table S3) among the Kuwaiti population.**

| **SNP_Effect allele, Position,** | **Associated trait^&^** | **Source** | **EAF** | **Sample Size** | **Effect Size^$^** | **P-value** | **Gene, Function consequence** | **Trait published in GWAS Catalog** |
| --- | --- | --- | --- | --- | --- | --- | --- | --- |
| rs112861901_C,  8:19750951 | HDL | OE^imputed^ | 0.11 | 1230 | 0.2824 | 1.33E-04 | INTS10, LPL;  Intergenic |  |
|  |  | CM^imputed^ | 0.107 | 1434 | 0.2434 | 8.54E-05 |  |  |
|  |  | Meta | 0.109 | 2664 | 5.4790 | 4.28E-08 |  |  |
| rs11132637_G,  4:190208378 | HDL | OE^imputed^ | 0.219 | 1230 | -0.1898 | 1.09E-03 | LOC105377613, LOC105377614;  downstream |  |
|  |  | CM^imputed^ | 0.222 | 1434 | -0.2303 | 7.65E-06 |  |  |
|  |  | Meta | 0.22 | 2664 | -5.5020 | 3.76E-08 |  |  |
| rs35387314_C,  4:190206771 | HDL | OE^imputed^ | 0.227 | 1230 | -0.1877 | 1.16E-03 | LOC105377613, LOC105377614;  downstream |  |
|  |  | CM^imputed^ | 0.238 | 1434 | -0.2165 | 6.65E-06 |  |  |
|  |  | Meta | 0.232 | 2664 | -5.5130 | 3.53E-08 |  |  |
| rs6846011_A,  4:190202004 | HDL | OE^imputed^ | 0.225 | 1230 | -0.1932 | 7.99E-04 | LOC105377613, LOC105377614;  intergenic |  |
|  |  | CM^imputed^ | 0.237 | 1434 | -0.2207 | 9.12E-06 |  |  |
|  |  | Meta | 0.231 | 2664 | -5.5340 | 3.13E-08 |  |  |
| rs34453299_A,  4:190201161 | HDL | OE^imputed^ | 0.218 | 1230 | -0.1966 | 7.04E-04 | LOC105377613, LOC105377614;  intergenic |  |
|  |  | CM^imputed^ | 0.221 | 1434 | -0.2350 | 1.05E-05 |  |  |
|  |  | Meta | 0.219 | 2664 | -5.5350 | 3.12E-08 |  |  |
| rs561984720_G,  4:190198217 | HDL | OE^imputed^ | 0.211 | 1230 | -0.2008 | 1.08E-03 | LOC105377613, LOC105377614;  intergenic |  |
|  |  | CM^imputed^ | 0.224 | 1434 | -0.2412 | 8.00E-06 |  |  |
|  |  | Meta | 0.217 | 2664 | -5.4970 | 3.85E-08 |  |  |
| rs9790417_A,  4:190191046 | HDL | OE^imputed^ | 0.227 | 1230 | -0.1863 | 1.09E-03 | LOC105377613, LOC105377614;  intergenic |  |
|  |  | CM^imputed^ | 0.238 | 1434 | -0.2307 | 9.06E-06 |  |  |
|  |  | Meta | 0.232 | 2664 | -5.4760 | 4.35E-08 |  |  |
| rs11942078_T,  4:190188001 | HDL | OE^imputed^ | 0.226 | 1230 | -0.1932 | 6.72E-04 | LOC105377613, LOC105377614;  intergenic |  |
|  |  | CM^imputed^ | 0.238 | 1434 | -0.2340 | 9.01E-06 |  |  |
|  |  | Meta | 0.232 | 2664 | -5.5680 | 2.58E-08 |  |  |
| rs35668405_A,  4:190184168 | HDL | OE^imputed^ | 0.226 | 1230 | -0.1917 | 7.36E-04 | LOC105377613, LOC105377614;  intergenic |  |
|  |  | CM^imputed^ | 0.237 | 1434 | -0.2380 | 8.68E-06 |  |  |
|  |  | Meta | 0.232 | 2664 | -5.5570 | 2.74E-08 |  |  |
| rs35602882_G,  4:190184043 | HDL | OE^imputed^ | 0.22 | 1230 | -0.1971 | 5.67E-04 | LOC105377613, LOC105377614;  intergenic |  |
|  |  | CM^imputed^ | 0.222 | 1434 | -0.2518 | 9.95E-06 |  |  |
|  |  | Meta | 0.221 | 2664 | -5.5840 | 2.35E-08 |  |  |
| rs35266160_C,  4:190179626 | HDL | OE^imputed^ | 0.221 | 1230 | -0.2011 | 4.61E-04 | LOC105377613, LOC105377614;  Intergenic |  |
|  |  | CM^imputed^ | 0.222 | 1434 | -0.2584 | 9.10E-06 |  |  |
|  |  | Meta | 0.221 | 2664 | -5.6360 | 1.74E-08 |  |  |
| rs11721957_A  4:190178544 | HDL | OE^imputed^ | 0.227 | 1230 | -0.1968 | 5.44E-04 | LOC105377613, LOC105377614;  Intergenic |  |
|  |  | CM^imputed^ | 0.237 | 1434 | -0.2446 | 8.04E-06 |  |  |
|  |  | Meta | 0.232 | 2664 | -5.6250 | 1.86E-08 |  |  |
| rs35466621_A,  4:190172499 | HDL | OE^imputed^ | 0.227 | 1230 | -0.2016 | 4.59E-04 | LOC105377613, LOC105377614;  Intergenic |  |
|  |  | CM^imputed^ | 0.237 | 1434 | -0.2514 | 7.86E-06 |  |  |
|  |  | Meta | 0.232 | 2664 | -5.6600 | 1.52E-08 |  |  |
| rs35699925_T,  4:190170843 | HDL | OE^imputed^ | 0.227 | 1230 | -0.2020 | 4.59E-04 | LOC105377613, LOC105377614;  Intergenic |  |
|  |  | CM^imputed^ | 0.236 | 1434 | -0.2529 | 7.75E-06 |  |  |
|  |  | Meta | 0.232 | 2664 | -5.6620 | 1.50E-08 |  |  |
| rs34329457_C,  4:190169159 | HDL | OE^imputed^ | 0.227 | 1230 | -0.2028 | 4.47E-04 | LOC105377613, LOC105377614;  Intergenic |  |
|  |  | CM^imputed^ | 0.236 | 1434 | -0.2541 | 7.74E-06 |  |  |
|  |  | Meta | 0.232 | 2664 | -5.6670 | 1.46E-08 |  |  |
| rs13147669_A,  4:190167174 | HDL | OE^imputed^ | 0.227 | 1230 | -0.2042 | 4.26E-04 | LOC105377613, LOC105377614;  intergenic |  |
|  |  | CM^imputed^ | 0.236 | 1434 | -0.2560 | 7.66E-06 |  |  |
|  |  | Meta | 0.232 | 2664 | -5.6770 | 1.37E-08 |  |  |
| rs76018028_C,  4:190161531 | HDL | OE^imputed^ | 0.227 | 1230 | -0.2082 | 3.65E-04 | LOC105377613, LOC105377614;  intergenic |  |
|  |  | CM^imputed^ | 0.236 | 1434 | -0.2605 | 7.69E-06 |  |  |
|  |  | Meta | 0.232 | 2664 | -5.7040 | 1.17E-08 |  |  |
| rs35192598_T,  4:190160826 | HDL | OE^imputed^ | 0.22 | 1230 | -0.2132 | 3.05E-04 | LOC105377613, LOC105377614;  intergenic |  |
|  |  | CM^imputed^ | 0.22 | 1434 | -0.2766 | 8.72E-06 |  |  |
|  |  | Meta | 0.22 | 2664 | -5.7160 | 1.09E-08 |  |  |
| rs55944922_T,  4:190159637 | HDL | OE^imputed^ | 0.227 | 1230 | -0.2090 | 3.59E-04 | LOC105377613, LOC105377614;  intergenic |  |
|  |  | CM^imputed^ | 0.236 | 1434 | -0.2620 | 7.74E-06 |  |  |
|  |  | Meta | 0.232 | 2664 | -5.7060 | 1.16E-08 |  |  |
| rs13141596_A,  4:190159385 | HDL | OE^imputed^ | 0.22 | 1230 | -0.2134 | 3.20E-04 | LOC105377613, LOC105377614  intergenic |  |
|  |  | CM^imputed^ | 0.22 | 1434 | -0.2781 | 8.99E-06 |  |  |
|  |  | Meta | 0.22 | 2664 | -5.7030 | 1.18E-08 |  |  |
| rs13120111_A,  4:190157839 | HDL | OE^imputed^ | 0.225 | 1230 | -0.2097 | 3.82E-04 | LOC105377613, LOC105377614;  intergenic |  |
|  |  | CM^imputed^ | 0.235 | 1434 | -0.2664 | 7.63E-06 |  |  |
|  |  | Meta | 0.23 | 2664 | -5.6970 | 1.22E-08 |  |  |
| rs13119288_G,  4:190157713  G | HDL | OE^imputed^ | 0.226 | 1230 | -0.2069 | 4.51E-04 | LOC105377613, LOC105377614;  intergenic |  |
|  |  | CM^imputed^ | 0.236 | 1434 | -0.2671 | 7.57E-06 |  |  |
|  |  | Meta | 0.231 | 2664 | -5.6680 | 1.44E-08 |  |  |
| rs36021514_A,  4:190157576 | HDL | OE^imputed^ | 0.225 | 1230 | -0.2105 | 3.73E-04 | LOC105377613, LOC105377614;  intergenic |  |
|  |  | CM^imputed^ | 0.235 | 1434 | -0.2674 | 7.53E-06 |  |  |
|  |  | Meta | 0.23 | 2664 | -5.7030 | 1.17E-08 |  |  |
| rs34728227_A,  4:190157486 | HDL | OE^imputed^ | 0.225 | 1230 | -0.2106 | 3.70E-04 | LOC105377613, LOC105377614;  intergenic |  |
|  |  | CM^imputed^ | 0.235 | 1434 | -0.2674 | 7.68E-06 |  |  |
|  |  | Meta | 0.23 | 2664 | -5.7020 | 1.18E-08 |  |  |
| rs35127507_A,  4:190157056 | HDL | OE^imputed^ | 0.225 | 1230 | -0.2104 | 3.83E-04 | LOC105377613, LOC105377614;  intergenic |  |
|  |  | CM^imputed^ | 0.234 | 1434 | -0.2679 | 7.82E-06 |  |  |
|  |  | Meta | 0.23 | 2664 | -5.6930 | 1.25E-08 |  |  |
| rs34706863_T,  4:190156646 | HDL | OE^imputed^ | 0.224 | 1230 | -0.2114 | 3.76E-04 | LOC105377613, LOC105377614;  intergenic |  |
|  |  | CM^imputed^ | 0.232 | 1434 | -0.2711 | 7.67E-06 |  |  |
|  |  | Meta | 0.228 | 2664 | -5.6990 | 1.20E-08 |  |  |
| rs289713_A,  16:57006829 | HDL | OE^imputed^ | 0.78 | 1230 | 0.2095 | 8.08E-05 | CETP  intronic |  |
|  |  | CM^imputed^ | 0.772 | 1434 | 0.2072 | 5.68E-06 |  |  |
|  |  | Meta | 0.776 | 2664 | 6.0080 | 1.88E-09 |  |  |
| rs7499892_T,  16:57006590^#^ | HDL | OE^imputed^ | 0.185 | 1230 | -0.2412 | 2.68E-05 | CETP;  intronic | HDL cholesterol |
|  |  | CM^genotyped^ | 0.191 | 1434 | -0.2310 | 1.14E-06 |  |  |
|  |  | Meta | 0.188 | 2664 | -6.4240 | 1.33E-10 |  |  |
| rs11076175_G,  16:57006378 | HDL | OE^imputed^ | 0.167 | 1230 | -0.2563 | 8.65E-06 | CETP;  intronic |  |
|  |  | CM^imputed^ | 0.178 | 1434 | -0.2523 | 3.02E-07 |  |  |
|  |  | Meta | 0.173 | 2664 | -6.7810 | 1.20E-11 |  |  |
| rs200751500_A,  16:57001274 | HDL | OE^imputed^ | 0.281 | 1230 | 0.1996 | 2.19E-04 | CETP;  intronic |  |
|  |  | CM^imputed^ | 0.278 | 1434 | 0.1782 | 1.61E-05 |  |  |
|  |  | Meta | 0.28 | 2664 | 5.6760 | 1.38E-08 |  |  |
| rs12720908_TCACA  16:57001254 | HDL | OE^imputed^ | 0.185 | 1230 | -0.1976 | 2.15E-04 | CETP;  Intronic |  |
|  |  | CM^imputed^ | 0.196 | 1434 | -0.1994 | 3.00E-05 |  |  |
|  |  | Meta | 0.19 | 2664 | -5.5770 | 2.45E-08 |  |  |
| rs12720922_A,  16:57000885 | HDL | OE^imputed^ | 0.185 | 1230 | -0.1983 | 2.13E-04 | CETP;  Intronic |  |
|  |  | CM^genotyped^ | 0.196 | 1434 | -0.1987 | 3.12E-05 |  |  |
|  |  | Meta | 0.19 | 2664 | -5.5720 | 2.52E-08 |  |  |
| rs17231569_C,  16:56999778 | HDL | OE^imputed^ | 0.187 | 1230 | -0.1962 | 2.67E-04 | CETP  Intronic |  |
|  |  | CM^imputed^ | 0.2 | 1434 | -0.1983 | 2.68E-05 |  |  |
|  |  | Meta | 0.193 | 2664 | -5.5580 | 2.73E-08 |  |  |
| rs11508026_T,  16:56999328^#^ | HDL | OE^imputed^ | 0.388 | 1230 | 0.2211 | 5.12E-06 | CETP  Intronic | C-reactive protein levels or triglyceride levels (pleiotropy) |
|  |  | CM^imputed^ | 0.381 | 1434 | 0.1299 | 6.28E-04 |  |  |
|  |  | Meta | 0.385 | 2664 | 5.6070 | 2.06E-08 |  |  |
| rs7203984_C,  16:56999258 | HDL | OE^imputed^ | 0.232 | 1230 | -0.1917 | 2.90E-04 | CETP  Intronic |  |
|  |  | CM^genotyped^ | 0.227 | 1434 | -0.1835 | 3.91E-05 |  |  |
|  |  | Meta | 0.23 | 2664 | -5.4800 | 4.25E-08 |  |  |
| rs12720926_G,  16:56998918 | HDL | OE^imputed^ | 0.389 | 1230 | 0.2180 | 6.43E-06 | CETP  intronic |  |
|  |  | CM^imputed^ | 0.378 | 1434 | 0.1390 | 2.74E-04 |  |  |
|  |  | Meta | 0.384 | 2664 | 5.7350 | 9.75E-09 |  |  |
| rs5817082_CA,  16:56997349 | HDL | OE^imputed^ | 0.285 | 1230 | -0.2246 | 2.25E-05 | CETP  intronic |  |
|  |  | CM^imputed^ | 0.32 | 1434 | -0.2148 | 6.56E-08 |  |  |
|  |  | Meta | 0.302 | 2664 | -6.8440 | 7.69E-12 |  |  |
| rs1864163_A,  16:56997233^#^ | HDL | OE^imputed^ | 0.25 | 1230 | -0.2494 | 2.63E-06 | CETP  intronic | HDL cholesterol; Total cholesterol levels; High density lipoprotein cholesterol levels |
|  |  | CM^genotyped^ | 0.286 | 1434 | -0.2285 | 1.92E-08 |  |  |
|  |  | Meta | 0.268 | 2664 | -7.3150 | 2.58E-13 |  |  |
| rs34620476_A,  16:56996649 | HDL | OE^imputed^ | 0.404 | 1230 | 0.1993 | 1.87E-05 | CETP  intronic |  |
|  |  | CM^genotyped^ | 0.383 | 1434 | 0.1509 | 6.37E-05 |  |  |
|  |  | Meta | 0.394 | 2664 | 5.8420 | 5.17E-09 |  |  |
| rs34145065_G,  16:56996645 | HDL | OE^imputed^ | 0.404 | 1230 | 0.2004 | 1.79E-05 | CETP;  Intronic |  |
|  |  | CM^imputed^ | 0.378 | 1434 | 0.1594 | 2.85E-05 |  |  |
|  |  | Meta | 0.391 | 2664 | 5.9850 | 2.16E-09 |  |  |
| rs708272_A,  16:56996288 | HDL | OE^imputed^ | 0.414 | 1230 | 0.2056 | 6.64E-06 | CETP;  Intronic |  |
|  |  | CM^imputed^ | 0.392 | 1434 | 0.1654 | 1.11E-05 |  |  |
|  |  | Meta | 0.403 | 2664 | 6.2850 | 3.28E-10 |  |  |
| rs711752_A,  16:56996211^#^ | HDL | OE^imputed^ | 0.415 | 1230 | 0.2057 | 6.67E-06 | CETP;  Intronic | C-reactive protein levels or HDL-cholesterol levels (pleiotropy) |
|  |  | CM^genotyped^ | 0.392 | 1434 | 0.1650 | 1.18E-05 |  |  |
|  |  | Meta | 0.403 | 2664 | 6.2760 | 3.49E-10 |  |  |
| rs3816117_C,  16:56996158 | HDL | OE^imputed^ | 0.52 | 1230 | 0.1876 | 2.74E-06 | CETP;  Intronic |  |
|  |  | CM^genotyped^ | 0.511 | 1434 | 0.1716 | 4.81E-06 |  |  |
|  |  | Meta | 0.515 | 2664 | 6.5410 | 6.09E-11 |  |  |
| rs1800775_A,  16:56995236^#^ | HDL | OE^genotyped^ | 0.519 | 1230 | 0.1869 | 2.05E-06 | CETP;  Upstream | HDL cholesterol; Lipid traits; HDL cholesterol levels; Coronary artery disease |
|  |  | CM^genotyped^ | 0.51 | 1434 | 0.1692 | 6.46E-06 |  |  |
|  |  | Meta | 0.515 | 2664 | 6.5360 | 6.32E-11 |  |  |
| rs17231506_T,  16:56994528^#^ | HDL | OE^imputed^ | 0.295 | 1230 | 0.1738 | 5.52E-04 | CETP;  Upstream | HDL cholesterol |
|  |  | CM^genotyped^ | 0.282 | 1434 | 0.1929 | 3.25E-06 |  |  |
|  |  | Meta | 0.288 | 2664 | 5.7620 | 8.32E-09 |  |  |
| rs36229491_TA,  16:56994244 | HDL | OE^imputed^ | 0.295 | 1230 | 0.1741 | 5.55E-04 | CETP;  Upstream |  |
|  |  | CM^imputed^ | 0.282 | 1434 | 0.1931 | 3.21E-06 |  |  |
|  |  | Meta | 0.288 | 2664 | 5.7630 | 8.28E-09 |  |  |
| rs821840_G,  16:56993886^#^ | HDL | OE^imputed^ | 0.314 | 1230 | 0.1842 | 1.60E-04 | CETP;  Upstream | Triglycerides |
|  |  | CM^imputed^ | 0.292 | 1434 | 0.2115 | 2.44E-07 |  |  |
|  |  | Meta | 0.303 | 2664 | 6.3530 | 2.12E-10 |  |  |
| rs3764261_A,  16:56993324^#^ | HDL | OE^imputed^ | 0.321 | 1230 | 0.1879 | 1.25E-04 | CETP  Upstream | HDL cholesterol; LDL cholesterol; Triglycerides; Waist circumference and related phenotypes; Lipid traits; Total cholesterol levels |
|  |  | CM^genotyped^ | 0.305 | 1434 | 0.1880 | 3.15E-06 |  |  |
|  |  | Meta | 0.313 | 2664 | 6.0260 | 1.68E-09 |  |  |
| rs12149545_A,  16:56993161^#^ | HDL | OE^imputed^ | 0.279 | 1230 | 0.1946 | 1.96E-04 | CETP  Upstream | Coronary artery disease |
|  |  | CM^genotyped^ | 0.27 | 1434 | 0.2115 | 4.49E-07 |  |  |
|  |  | Meta | 0.274 | 2664 | 6.2330 | 4.57E-10 |  |  |
| rs201825234_A,  16:56991948 | HDL | OE^imputed^ | 0.17 | 1230 | -0.2475 | 2.70E-04 | CETP  Upstream |  |
|  |  | CM^imputed^ | 0.194 | 1434 | -0.1967 | 2.14E-05 |  |  |
|  |  | Meta | 0.182 | 2664 | -5.5930 | 2.23E-08 |  |  |
| rs6499862_A,  16:56991524 | HDL | OE^imputed^ | 0.179 | 1230 | -0.2214 | 8.66E-04 | CETP  Upstream |  |
|  |  | CM^genotyped^ | 0.207 | 1434 | -0.1983 | 8.64E-06 |  |  |
|  |  | Meta | 0.193 | 2664 | -5.5270 | 3.25E-08 |  |  |
| rs6499861_G,  16:56991495 | HDL | OE^imputed^ | 0.172 | 1230 | -0.2403 | 3.84E-04 | CETP  Upstream |  |
|  |  | CM^imputed^ | 0.194 | 1434 | -0.2000 | 1.25E-05 |  |  |
|  |  | Meta | 0.183 | 2664 | -5.6180 | 1.94E-08 |  |  |
| rs183130_T  16:56991363^#^ | HDL | OE^imputed^ | 0.312 | 1230 | 0.1801 | 3.31E-04 | CETP  Upstream | HDL cholesterol |
|  |  | CM^genotyped^ | 0.289 | 1434 | 0.1966 | 1.69E-06 |  |  |
|  |  | Meta | 0.3 | 2664 | 5.9520 | 2.66E-09 |  |  |
| rs247617_A,  16:56990716^#^ | HDL | OE^imputed^ | 0.311 | 1230 | 0.1802 | 3.66E-04 | HERPUD1, CETP  Upstream | HDL cholesterol; LDL cholesterol |
|  |  | CM^genotyped^ | 0.288 | 1434 | 0.1954 | 2.02E-06 |  |  |
|  |  | Meta | 0.299 | 2664 | 5.9080 | 3.46E-09 |  |  |
| rs247616_T,  16:56989590^#^ | HDL | OE^imputed^ | 0.311 | 1230 | 0.1812 | 3.63E-04 | HERPUD1, CETP  Upstream | Coronary artery disease (myocardial infarction, percutaneous transluminal coronary angioplasty, coronary artery bypass grafting, angina or chromic ischemic heart disease); Cardiovascular risk factors; LDL cholesterol levels |
|  |  | CM^imputed^ | 0.289 | 1434 | 0.1946 | 2.37E-06 |  |  |
|  |  | Meta | 0.3 | 2664 | 5.8850 | 3.98E-09 |  |  |
| rs2399597_A,  16:56988502 | HDL | OE^imputed^ | 0.171 | 1230 | -0.2398 | 4.96E-04 | RPS24P17 Upstream |  |
|  |  | CM^imputed^ | 0.193 | 1434 | -0.1952 | 2.40E-05 |  |  |
|  |  | Meta | 0.182 | 2664 | -5.4660 | 4.61E-08 |  |  |
| rs173539_T,  16:56988044^#^ | HDL | OE^imputed^ | 0.334 | 1230 | 0.2108 | 3.42E-05 | HERPUD1, CETP  Upstream | Cardiovascular disease risk factors; HDL cholesterol; HDL Cholesterol - Triglycerides (HDLC-TG) |
|  |  | CM^genotyped^ | 0.32 | 1434 | 0.1701 | 2.39E-05 |  |  |
|  |  | Meta | 0.327 | 2664 | 5.9150 | 3.32E-09 |  |  |
| rs56228609_T,  16:56987765^#^ | HDL | OE^imputed^ | 0.28 | 1230 | 0.1992 | 2.44E-04 | HERPUD1, CETP  Upstream | Coronary artery disease |
|  |  | CM^imputed^ | 0.268 | 1434 | 0.2068 | 9.66E-07 |  |  |
|  |  | Meta | 0.274 | 2664 | 6.0870 | 1.15E-09 |  |  |
| rs56156922_C,  16:56987369^#^ | HDL | OE^imputed^ | 0.294 | 1230 | 0.1819 | 6.57E-04 | HERPUD1, CETP  upstream | Total cholesterol levels; HDL cholesterol levels; Triglyceride levels |
|  |  | CM^genotyped^ | 0.281 | 1434 | 0.1930 | 3.67E-06 |  |  |
|  |  | Meta | 0.287 | 2664 | 5.7120 | 1.12E-08 |  |  |
| rs12446515_T,  16:56987015 | HDL | OE^imputed^ | 0.293 | 1230 | 0.1822 | 6.54E-04 | HERPUD1, CETP  upstream |  |
|  |  | CM^genotyped^ | 0.276 | 1434 | 0.1868 | 7.38E-06 |  |  |
|  |  | Meta | 0.285 | 2664 | 5.6040 | 2.09E-08 |  |  |
| rs7205692_G,  16:56986914 | HDL | OE^imputed^ | 0.185 | 1230 | -0.2490 | 2.28E-04 | HERPUD1, CETP  upstream |  |
|  |  | CM^genotyped^ | 0.198 | 1434 | -0.1907 | 2.68E-05 |  |  |
|  |  | Meta | 0.191 | 2664 | -5.5850 | 2.34E-08 |  |  |
| rs7203286  16:56986762 | HDL | OE^imputed^ | 0.463 | 1230 | -0.1578 | 3.77E-03 | HERPUD1, CETP;  upstream |  |
|  |  | CM^imputed^ | 0.493 | 1434 | -0.1842 | 1.21E-06 |  |  |
|  |  | Meta | 0.478 | 2664 | -5.5300 | 3.21E-08 |  |  |
| rs11862052_T,  16:56985805 | HDL | OE^imputed^ | 0.174 | 1230 | -0.2709 | 1.34E-04 | HERPUD1, CETP;  regulatory region |  |
|  |  | CM^imputed^ | 0.182 | 1434 | -0.1954 | 7.93E-05 |  |  |
|  |  | Meta | 0.178 | 2664 | -5.4900 | 4.02E-08 |  |  |
| rs10635970_TAA,  8:19745039 | LDL | OE^imputed^ | 0.497 | 1165 | -0.1675 | 1.29E-04 | INTS10, LPL;  intergenic |  |
|  |  | CM^imputed^ | 0.504 | 1415 | -0.1604 | 4.31E-05 |  |  |
|  |  | Meta | 0.5 | 2580 | -5.6020 | 2.12E-08 |  |  |
| rs66505542_T,  11:116623213 | TG | OE^imputed^ | 0.746 | 1234 | -0.1751 | 3.27E-04 | BUD13;  Intronic |  |
|  |  | CM^imputed^ | 0.717 | 1434 | -0.1832 | 9.41E-06 |  |  |
|  |  | Meta | 0.731 | 2668 | -5.6920 | 1.26E-08 |  |  |
| rs78686130_T, 8:4128311 | FPG | OE^imputed^ | 0.306 | 1277 | -0.177 | 2.20E-03 | CSMD1, intronic |  |
|  |  | CM^imputed^ | 0.282 | 1434 | -0.267 | 2.67E-06 |  |  |
|  |  | Meta | 0.294 | 2711 | 5.509 | 3.61E-08 |  |  |
|  |  |  |  |  |  |  |  |  |
| rs7838666_C, 8:4126701 | FPG | OE^imputed^ | 0.689 | 1277 | 0.236 | 3.92E-05 | CSMD1, intronic |  |
|  |  | CM^imputed^ | 0.685 | 1434 | 0.254 | 4.47E-06 |  |  |
|  |  | Meta | 0.68 | 2711 | 6.159 | 7.31E-10 |  |  |
|  |  |  |  |  |  |  |  |  |
| rs2920844_T, 2:55341367 indexed | DBP | OE^imputed^ | 0.860 | 1246 | 0.184 | 2.08E-03 | RTN4, upstream |  |
|  |  | CM^imputed^ | 0.846 | 1430 | 0.272 | 1.50E-06 |  |  |
|  |  | Meta | 0.852 | 2676 | 5.617 | 1.94E-08 |  |  |
|  |  |  |  |  |  |  |  |  |
| rs2968781_G, 2:55341952 | DBP | OE^imputed^ | 0.860 | 1246 | 0.184 | 2.09e-03 | RTN4, upstream |  |
|  |  | CM^imputed^ | 0.847 | 1430 | 0.271 | 1.48E-06 |  |  |
|  |  | Meta | 0.852 | 2676 | 5.618 | 1.93E-08 |  |  |
|  |  |  |  |  |  |  |  |  |
| rs2920844_T, 2:55341367 | SBP | OE^imputed^ | 0.860 | 1246 | 0.202 | 5.32E-04 | RTN4, upstream |  |
|  |  | CM^imputed^ | 0.846 | 1430 | 0.257 | 5.37E-06 |  |  |
|  |  | Meta | 0.852 | 2676 | 5.69 | 1.27E-08 |  |  |
| rs2968781_G, 2:55341952 | SBP | OE^imputed^ | 0.860 | 1246 | 0.202 | 5.42E-04 | RTN4, upstream |  |
|  |  | CM^imputed^ | 0.847 | 1430 | 0.257 | 5.27E-06 |  |  |
|  |  | Meta | 0.852 | 2676 | 5.688 | 1.29E-08 |  |  |
|  |  |  |  |  |  |  |  |  |
| rs2835788_G, 21:38906071 | SBP | OE^imputed^ | 0.120 | 1246 | 0.241 | 1.91E-03 | DYRK1A, LOC105372798, intergenic |  |
|  |  | CM^imputed^ | 0.118 | 1430 | 0.316 | 2.51E-06 |  |  |
|  |  | Meta | 0.119 | 2676 | 5.559 | 2.72E-08 |  |  |
|  |  |  |  |  |  |  |  |  |

**^&^,** the traits are in inverse normal transformed form**.**

**^$^,** The effect size in the cases of CM and OM association studies denotes the resulting regression coefficient (beta) from the fit; and in the case of Meta-analysis denotes Z-score from Z-statistics.

**^#^,** For these variants, association with phenotype traits are seen in GWAS Catalog**.**
